# Integration of Transcriptome, Proteome and Metabolism Data Reveals the Alkaloids Biosynthesis in *Macleaya cordata* and *Macleaya microcarpa*

**DOI:** 10.1371/journal.pone.0053409

**Published:** 2013-01-09

**Authors:** Jianguo Zeng, Yisong Liu, Wei Liu, Xiubing Liu, Fuqing Liu, Peng Huang, Pengcheng Zhu, Jinjun Chen, Mingming Shi, Fang Guo, Pi Cheng, Jing Zeng, Yifang Liao, Jing Gong, Hong-Mei Zhang, Depeng Wang, An-Yuan Guo, Xingyao Xiong

**Affiliations:** 1 Hunan Provincial Key Laboratory of Crop Germplasm Innovation and Utilization and National Chinese Medicinal Herbs (Hunan) Technology Center, Hunan Agricultural University, Changsha, China; 2 Department of Biomedical Engineering, College of Life Science and Technology, Huazhong University of Science and Technology, Wuhan, China; 3 Herbal Extract Engineering Research Center (HerbEx), Hunan, China; 4 Nextomics Biosciences Co., Ltd. Wuhan, China; 5 Micolta BioResource Inc., Hunan, China; 6 BGI–Shenzhen, Shenzhen, China; Centro de Investigación y de Estudios Avanzados del IPN, Mexico

## Abstract

**Background:**

The *Macleaya* spp., including *Macleaya cordata* and *Macleaya microcarpa*, are traditional anti-virus, inflammation eliminating, and insecticide herb medicines for their isoquinoline alkaloids. They are also known as the basis of the popular natural animal food addictive in Europe. However, few studies especially at genomics level were conducted on them. Hence, we performed the *Macleaya* spp. transcriptome and integrated it with iTRAQ proteome analysis in order to identify potential genes involved in alkaloids biosynthesis.

**Methodology and Principal Findings:**

We elaborately designed the transcriptome, proteome and metabolism profiling for 10 samples of both species to explore their alkaloids biosynthesis. From the transcriptome data, we obtained 69367 and 78255 unigenes for *M. cordata* and *M. microcarpa*, in which about two thirds of them were similar to sequences in public databases. By metabolism profiling, reverse patterns for alkaloids sanguinarine, chelerythrine, protopine, and allocryptopine were observed in different organs of two species. We characterized the expressions of enzymes in alkaloid biosynthesis pathways. We also identified more than 1000 proteins from iTRAQ proteome data. Our results strongly suggest that the root maybe the organ for major alkaloids biosynthesis of *Macleaya* spp. Except for biosynthesis, the alkaloids storage and transport were also important for their accumulation. The ultrastructure of laticifers by SEM helps us to prove the alkaloids maybe accumulated in the mature roots.

**Conclusions/Significance:**

To our knowledge this is the first study to elucidate the genetic makeup of *Macleaya* spp. This work provides clues to the identification of the potential modulate genes involved in alkaloids biosynthesis in *Macleaya* spp., and sheds light on researches for non-model medicinal plants by integrating different high-throughput technologies.

## Introduction

*Macleaya* spp. is a genus of the Papaveraceae family and includes two species: *Macleaya cordata* (Willd.) R. Br. and *Macleaya microcarpa* (Maxim.) Fedde, which are perennial herb plants. The herbs are mainly distributed in China, Southeast Asia, North America, and Europe and were utilized as traditional medicines for a long time [1]. They are called ‘Boluohui’ in the famous Chinese book “Compendium of Materia Medica” which was written 300 years ago.

During the past decade or so, the *Macleaya* spp. has been famous for its application in the zootechny. In Germany, the *Macleaya cordata* (*M. cordata*) has been planted for animal food additive and biogas additive production, and was recorded on the European Food Safety Authority (EFSA) list of plants exploited as a component in feed additives in animal production [2], [3]. Many benzylisoquinoline alkaloids (BIAs), including sanguinarine (SA), chelerythrine (CHE), protopine (PRO), allocryptopine (ALL), dihydrochelerythrine (DHCHE) and dihydrosanguinarine (DHSA) were extracted from *Macleaya* spp. Pharmaceutical researches showed that these alkaloids were its major bio-active components [4], [5], [6], [7]. Recently, it has been found that SA and CHE had antiseptic and antitumor activities [8], [9], [10]. Sanguiritrin, a mixture of SA and CHE extracted from *M. cordata* was approved as a new antimicrobial drug by E. V. Arzamastsev et al. [1], [11]. Although the alkaloids from *Macleaya* spp. exhibited a great variety of pharmacological activities and have been applied widely, the molecular genetics study of it is rare. Currently, there are only less than 20 sequences from *M. cordata* in NCBI GenBank. Considering this, it is essential to apply high-throughput technologies, especially the prevalent deep sequencing technology, to study its molecular genetics and alkaloid biosynthesis.

**Figure 1:**
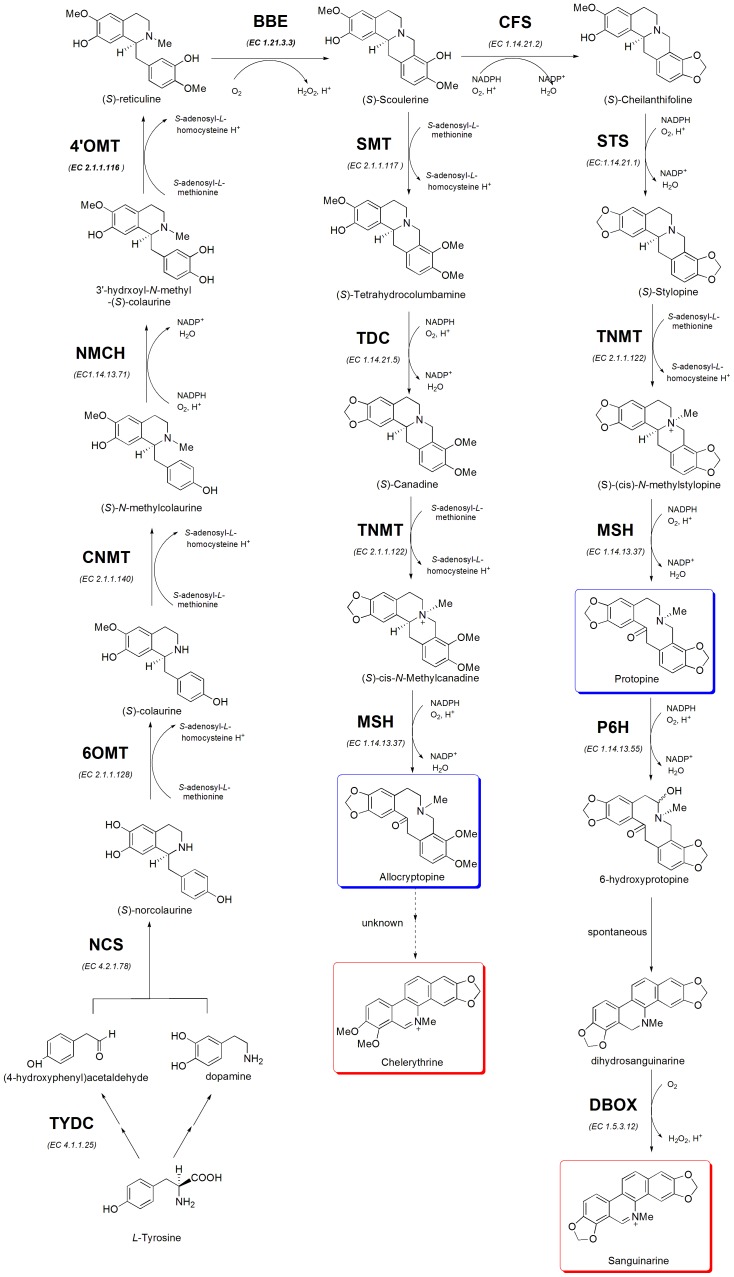
Biosynthetic pathway from tyrosine to SA, CHE, PRO and ALL. The biosynthesis of BIAs starts with the condensation of two tyrosine derivatives, followed by serial reactions to form *(S)*-reticuline. **Abbreviations:** TYDC, tyrosine/dopa decarboxylase; NCS, norcoclaurine synthase; 6OMT, *(S)*-norcoclaurine 6-O-methyltransferase;CNMT, *(S)*-coclaurine N-methyltransferase; NMCH, *(S)*-N-methylcoclaurine 3′-hydroxylase; 4′OMT, *(S)*-3′-hdroxy-Nmethylcoclaurine 4′-O-methyltransferase; BBE, berberine bridge enzyme; CFS, cheilanthifoline synthase; STS, stylopine synthase; SMT, *(S)*-scoulerine 9-O-methyltransferase; TDC, *(S)*-tetrahydroberberine synthase;TNMT, tetrahydroprotoberberine N-methyltransferase; MSH, methylstylopine hydroxylase; P6H, protopine 6-hydroxylase; DBOX, dihydrobenzophenanthridine oxidase.

The biosynthesis of BIAs starts with the condensation of two tyrosine derivatives, followed by serial reactions to form *(S)*-reticuline, which was summarized in many review papers [12]. *(S)*-reticuline is the central intermediate and common precursor of most BIAs. Here, we focus on reviewing the pathway from *(S)*-reticuline to SA (Figure 1). The reaction catalyzed by berberine bridge enzyme (BBE) represents the first committed step in the branch pathway leading to the benzophenanthridine alkaloid SA. The *(S)*-scoulerine then being catalyzed the formation of two methylenedioxy bridges and yield *(S)*-stylopine with the help of two P450-dependent enzymes, cheilanthifoline synthase (CFS) and stylopine synthase (STS) [13], [14]. Thereafter, the tetrahydroprotoberberine cis-N-methyltransferase (TNMT) converts *(S)*-stylopine to *(S)*-cis-N-methylstylopine [15]. N-methylstylopine 14-hydroxylase (MSH) and protopine 6-hydroxylase (P6H) both belongs to the P450 enzymes are responsible for the conversion of *(S)*-cis-N-methylstylopine to 6-hydroxyprotopine [16]. Last but not least, dihydrosanguinarine is oxidized to SA by the oxidoreductase dihydrobenzophenanthridine oxidase (DBOX) [17], [18]. The alkaloids ALL and CHE was biosynthesized from another pathway started from the *(S)*-scoulerine, which is similar to PRO and SA [19] (see Figure 1). Most of the cognate cDNAs and protein sequences have been reported for the aforementioned enzymes with the exception of MSH and DBOX though they were already purified by several researchers [18], [20].

Our previous survey of *Macleaya* spp. found that the accumulation levels of different alkaloids were significantly various for different organs (roots, leaves and fruits) and species (*M. cordata* and *M. microcarpa*). For example, the level of SA varied from 0.5% to 4% in different samples [10]. The alkaloids may be biosynthesized in the sieve elements and companion cells, and then accumulated in the adjacent laticifers based on the studies in opium poppy [12], [21], [22], [23]. Through extensive intra- and intercellular transport by transporters including ABC transporters, alkaloids were transported and distributed in different organs [24]. Desgagne-Penix et al., studied secondary metabolism of alkaloids in the way of integrating 454 GS-FLX pyrosequencing and LC-MS/MS-based protein profiling technologies in elicitor-treated opium poppy cell cultures and revealed enzymes catalyzing steps in BIAs biosynthesis [25].

RNA-Seq technology has the potential to provide deep coverage and unbiased representation of transcript abundance, which is very important in non-model plants lacking genome sequence information [26]. However, the frequent incongruity between protein levels and the abundance of cognate gene transcripts [27] is crucial to the interpretation of relative gene expression profiles. Complementary analysis of the proteome combined with a comprehensive transcriptome provides an important validation tool for the expression of key genes. Metabolite profiling is essential in the study of alkaloid metabolism. With the combination of transcriptome, proteome and metabolite profiling, we can reveal the correlation between the expression of alkaloid biosynthetic genes and corresponding metabolic products which will help to explore the application research of alkaloids biosynthesis mechanism [12], [28].

The *M. cordata* and *M. microcarpa* are the only two natural species in Macleaya genus with many differences on the fruits shape and alkaloid accumulation. The alkaloids extracted from them with various pharmacological activities and were utilized in different products. Dissecting the relationship between gene expression, regulation and alkaloids synthesis will potentially develop molecular approaches to improve the alkaloid production, which have great economical and social values.

Here, we report the systematic identification and characterization of *Macleaya* spp. transcriptome and proteome. We elaborately chose 10 samples from different phases (before flowering and after fruits matured) and organs (roots, leaves and fruits) of both species (*M. cordata* and *M. microcarpa*) to perform transcriptome, proteome and metabolism analysis. We got more than 60000 unigenes for both species from the transcriptome data, including almost all of the known enzymes in the alkaloids synthesis. Through LC-MS/MS peptide analysis, we facilitated the match of about 4, 000 peptides and identified more than 1000 non-redundant proteins. Combined the transcriptome and proteome data with metabolism data of alkaloids accumulation, we identified the alkaloids biosynthesis tissues in different phases and the expression of those enzymes. These results will be helpful for further molecular breeding to improve the alkaloids production in *Macleaya* spp. The integration of transcriptome, proteome and metabolism data for functional genomics research in this study also shed light on researches of non-model species without genome sequence available especially medicinal plants.

## Results

### Sample Selection

**Figure 2:**
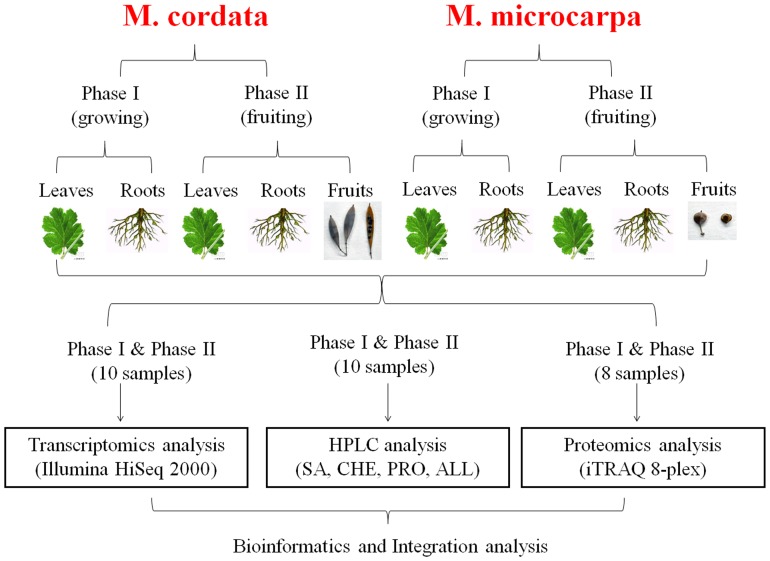
The strategy overview. Ten special samples of different time and organs to perform transcriptome, metabolism and proteome analysis were selected to explore their alkaloids biosynthesis. These samples are from Phase I (roots, leaves) and Phase II (roots, leaves and fruit shells) of both *M. cordata* and *M. microcarpa*.

Since *M. cordata* and *M. microcarpa* are two species of Macleaya genus with different fruits shape and alkaloids accumulation organs, we study the alkaloids biosynthesis at different times for both species. We elaborately selected 10 special samples to perform transcriptome, metabolism and proteome analysis. These samples are from Phase I (roots, leaves) and Phase II (roots, leaves and fruit shells) of both *M. cordata* and *M. microcarpa*. Phase I is chosen at the time point which the whole plant just grows up and completes the vegetative growth. We took the roots and leaves as the representatives of this phase. Phase II is chosen at the time point in which the fruits are mature and the alkaloids are at a relatively high level. We took the roots, leaves and fruits as the representatives of this phase. As a summary, these samples and their identifiers are shown in Figure 2.

### The Alkaloids Accumulation Organs at Different Phases of both Macleaya Plants

Since we aim to explore the alkaloids biosynthesis, firstly, we investigated the four major alkaloids as the model of metabolite profile for the 10 samples of both species described above. We found various alkaloids accumulation in different organs of different species (Table 1). In *M. cordata*, the SA and CHE in fruits were significantly higher than those in leaves, while the PRO and ALL accumulations were inverse in fruits and leaves. On the contrary, it is different in the *M. microcarpa* (Table 1). The PRO and ALL in *M. microcarpa* fruits were significantly higher than in leaves, while the SA and CHE in leaves were significantly higher than in fruits. These indicated that the SA and CHE were accumulated in the fruits of *M. cordata* and leaves of *M. microcarpa*, while the PRO and ALL were accumulated in the leaves of *M. cordata* and fruits of *M. microcarpa* (Table 1). The roots of both species at both phases always had low SA and CHE, but high PRO and ALL accumulation. We noticed that all the 4 alkaloids had the similar accumulation levels in the two phases of the same organ of the same species (e.g. DGY9801 and DGY1501). The SA and CHE are always at the similar level as a group in all samples, while PRO and ALL are similar as another group in all samples.

**Table 1:** The alkaloids accumulation in different parts and different phase of *Macleaya* plant.

	Sample	Number	SA(mg/g)	CHE(mg/g)	PRO(mg/g)	ALL(mg/g)
*M.* *cordata*	Leaves	DGY9801	2.5	2.3	30.6	14.3
		DGY1501	0.7	0.5	14	9.3
	Roots	DGG9803	0.2	0.4	20.6	27.5
		DGG1501	1	0.8	18.6	27.4
	Fruits	DGU1501	8.1	2.9	5.3	3.8
*M.* *microcarpa*	Leaves	XGY9802	8.7	9.2	2.7	3.8
		XGY1502	7.1	8.3	2.7	4.8
	Roots	XGG9804	0.3	0.3	17.6	53.9
		XGG1502	0.9	0.8	20	68.2
	Fruits	XGU1502	0.3	0.5	1.1	40.7

SA: sanguinarine; CHE: chelerythrine; PRO: protopine; ALL: allocryptopine.

### Transcriptome Data de Novo Assembly

To obtain an overview of the gene expression of both *M. cordata* and *M. microcarpa*, transcriptome for the same samples used in the metabolite profile analysis were sequenced using the Illumina platform (Figure 2). After removal of adapter sequences, duplication sequences, ambiguous reads and low-quality reads, we got 66.7 million high-quality clean reads (6.01 Gbp) for *M. cordata* and 66.8 million high-quality clean reads (6.02 Gbp) for *M. microcarpa* (Table S1). All high-quality reads of each sample were de novo assembled individually by the Trinity software [29]. The summary of the assembled results was shown in Table 2. Averagely, we obtained assembled contigs for each sample of *M. cordata* and *M. microcarpa* with N50 from 629 to 837 bp, respectively (Table 2). About 13.50% of these contigs are longer than 1000 bp. Then, in order to get the full length transcripts and gene sets of *M. cordata* and *M. microcarpa*, we further clustered all the assembled contigs from *M. cordata* and *M. microcarpa* into two unigene pools for *M. cordata* and *M. microcarpa* (Table 2). As a result, we obtained 69367 unigenes for *M. cordata* with an average length of 796 bp and an N50 of 1286 bp. We also got 78255 unigenes for *M. microcarpa* with an average length of 740 bp and an N50 of 1208 bp. The size distributions for these unigenes were shown in Figure S1. In order to evaluate the quality of the assembled unigenes, we also sequenced and obtained 395 EST sequences from *M. cordata*. After BLAST (e value<1e−10), we found that more than 83% EST sequences could align to our assembled unigenes with identity >90%. These suggested that our assembled unigenes from transcriptome data were reliable as a non-model species without genome. To identify the orthologous genes between *M. cordata* and *M. microcarpa*, using the double best hit approach (see methods), we identified 27055 pairs of unigenes in these two species.

**Table 2:** Summary of Macleaya spp. sequence assembly.

	Sequences(n)	Base pairs (Mbp)	Mean length (bp)	N50 (bp)	Samples	Max(bp)	Min(bp)	Mean length(bp)	Total seq	N50(bp)
***M.cordata***	66,777,790	6,010.00	90	–	DGG1501	9,912	201	628	77,491	837
Singltons*	29575	–	–	–	DGG9803	6,319	201	564	76,719	716
Clusters**	39792	–	–	–	DGU1501	9,995	201	625	82,996	837
Total unigenes	69367	55.20	796	1286	DGY1501	11,020	201	596	77,696	788
					DGY9801	8,249	201	531	88,384	651
***M. microcarpa***	66,844,460	6,016.00	90	–	XGG1502	7,366	201	524	99,906	629
Singletons	37202	–	–	–	XGG9804	6,820	201	554	73,286	696
Clusters	41053	–	–	–	XGU1502	9,874	201	577	90,899	758
Total unigenes	78255	57.87	740	1208	XGY1502	5,753	201	566	78,110	719
					XGY9802	9,050	201	579	91,590	764

* Singletons of TGICL cluster results.

** Contigs of TGICL cluster results.

### Functional Annotation and Classification of the Assembled Unigenes

**Figure 3:**
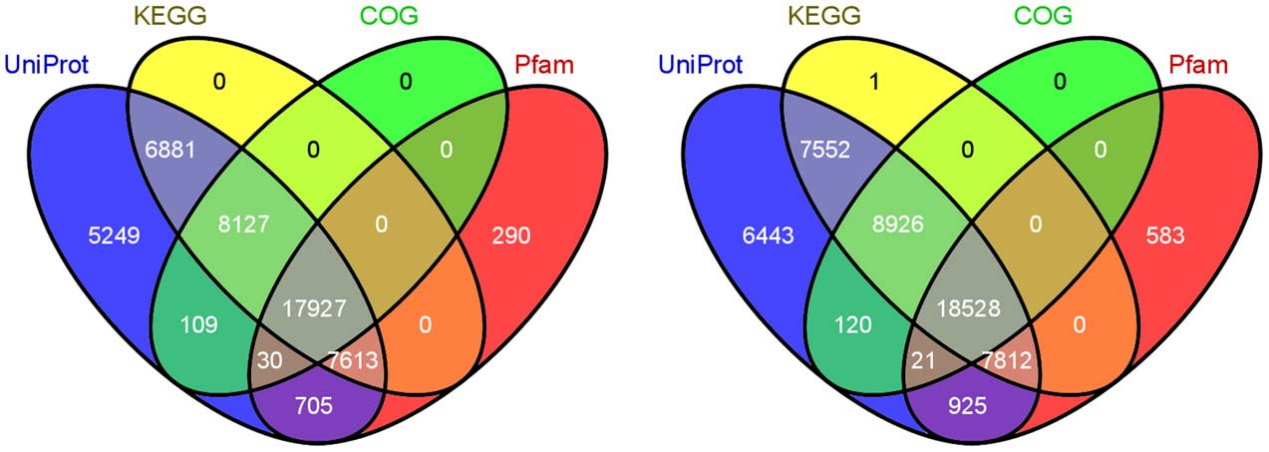
Venn diagram shows distribution of similarity search results. The unigenes annotations of both *M. cordata* and *M. microcarpa* were performed by alignment to public databases, including UniProt(Blue), KEGG (the Kyoto Encyclopedia of Genes and Genomes database, Yellow), COGs (Clusters of Orthologous Groups of proteins, Green) and Pfam(Red).

Annotations of the unigenes of both *M. cordata* and *M. microcarpa* were performed by alignment to public databases, including UniProt, the Kyoto Encyclopedia of Genes and Genomes database (KEGG), Gene Ontology (GO), COGs (Clusters of Orthologous Groups of proteins) and Pfam. Annotations of the best BLASTX hits and domain hits are summarized in Table 3 and their overlaps were shown in Figure 3. Totally, there are 46931 unigenes (67.66%) of *M. cordata* and 50912 unigenes (65.06%) of *M. microcarpa* significantly similar to entries of these public databases. Thus, remain more than 30% of these unigenes without annotation. UniProt is the most comprehensive protein sequence database and our unigenes have the most hits in it. The hit COG clusters were grouped into 24 function categories and the distributions of genes in two species were closely similar to each other (Table 3 and Figure S2). Furthermore, 17927 and 18528 unigenes of both species had hits in four public databases with relatively defined functional annotations (Figure 3). These are the first genome-wide geneset for *Macleaya* spp. and provide valuable resources for its further studies.

**Table 3:** Functional annotation and classification of the unigenes.

	*M. cordata*		*M. microcarpa*	
	number	percentage	number	percentage
**COG**	26193	37.76%	27595	35.26%
**GO**	33181	47.83%	35629	45.53%
**KEGG**	40548	58.45%	42819	54.72%
**Pfam**	26565	38.30%	27869	35.61%
**UniProt**	46641	67.24%	50327	64.31%
**Total alignments**	46931	67.66%	50912	65.06%

Pfam is comprehensive database for protein functional domain and family annotations. To get the functional domain distribution of proteins encoded by our assembled unigenes from *Macleaya* spp., we used the ESTscan software to translate the unigenes to proteins and used HMMER software to search the Pfam domain profiles. The results showed that there were 26565 and 27869 proteins aligned to 3270 and 3421 Pfam domains/families in *M. cordata* and *M. microcarpa*, respectively. The top 10 most frequently detected domains were identical in both species. They were “Protein kinase”, “RNA recognition motif”, “Cytochrome P450”, “Tyrosine-protein kinase”, “Pentatricopeptide repeat”, “WD40-repeat”, “Myb-like DNA-binding domain”, “Reverse transcriptase (RNA-dependent DNA polymerase)”, “Zinc finger C3HC4 type” and “Helicase conserved C-terminal domain” (see Figure S3).

GO is a common database to annotate genes into three major categories, namely biological process, molecular function, and cellular component [30]. As a result, 33178 unigenes from *M. cordata* (43.87%) and 35629 unigenes from *M. microcarpa* (45.53%) were respectively assigned to 84379 and 90455 GO terms. These GO terms were summarized into the three main GO categories and 43 sub-categories (Table S2 and Figure S4). For *M. cordata*, molecular function made up the majority of the GO annotations (29,772, 35.2%), followed by cellular component (15,459, 18.3%) and molecular function (12,293, 14.6%). While for *M. microcarpa*, the hit GO terms in cellular component, biological process and molecular function are 32.9%, 13.6% and 14.8%, respectively. However, this result showed only a small proportion of unigenes with GO annotation, possibly due to large number of uninformative gene descriptions of these plant protein hits.

In order to identify the active biological pathways in *M. cordata* and *M. microcarpa*, the assembled unigenes were annotated with corresponding Enzyme numbers (EC numbers) in the KEGG database [31]. By mapping EC numbers to the reference canonical pathways, a total of 40,548 unigenes in *M. cordata* and 42,819 unigenes in *M. microcarpa* were assigned to 264 KEGG pathways (Table 4). The most enriched pathways in *M. cordata* were Oxidative phosphorylation [PATH: ko00190] (291 members), Purine metabolism [PATH: ko00230] (290 members), and Starch and sucrose metabolism [PATH: ko00500] (261 members). It was interesting that the Methane metabolism [PATH: ko00680] (235 members) is located at the 6^th^ position of pathway enrichment. The pathways enriched mostly in *M. microcarpa* represented similar to *M. cordata*. The Isoquinoline alkaloid biosynthesis [PATH: ko00950] were out of the top 10 in both species but still relatively enrichment (55 and 59 members).

**Table 4:** The top 10 KEGG pathways of Macleaya spp.

M. cordata		M. microcarpa	
Pathway	number	Pathway	number
Oxidative phosphorylation [PATH:ko00190]	291	Oxidative phosphorylation [PATH:ko00190]	307
Purine metabolism [PATH:ko00230]	290	Purine metabolism [PATH:ko00230]	295
Starch and sucrose metabolism [PATH:ko00500]	261	Glycolysis/Gluconeogenesis [PATH:ko00010]	279
Amino sugar and nucleotide sugar metabolism [PATH:ko00520]	237	Starch and sucrose metabolism [PATH:ko00500]	271
Glycolysis/Gluconeogenesis [PATH:ko00010]	236	Amino sugar and nucleotide sugar metabolism [PATH:ko00520]	248
Methane metabolism [PATH:ko00680]	235	Ubiquitin mediated proteolysis [PATH:ko04120]	239
Ubiquitin mediated proteolysis [PATH:ko04120]	201	Methane metabolism [PATH:ko00680]	206
Pyrimidine metabolism [PATH:ko00240]	194	Pyrimidine metabolism [PATH:ko00240]	205
Phenylpropanoid biosynthesis [PATH:ko00940]	192	Pyruvate metabolism [PATH:ko00620]	200
Cysteine and methionine metabolism [PATH:ko00270]	167	Carbon fixation in photosynthetic organisms [PATH:ko00710]	196
*Isoquinoline alkaloid biosynthesis [PATH:ko00950]	55	Isoquinoline alkaloid biosynthesis [PATH:ko00950]	59

* Isoquinoline alkaloid biosynthesis is out of TOP20, so we list it separately for our main intersected in it.

### Cytochrome P450 Genes in Macleaya spp

Cytochrome P450 is a large group of enzymes involved in many important metabolism pathways and many enzymes in the benzylisoquinoline alkaloid biosynthesis belong to the P450 family, such as the stylopine synthase (CYP719A). Here we identified and classified the P450 unigenes according to the P450 protein sequences collected from Arabidopsis, rice, opium poppy, and *Coptis japonica*. We defined the opium poppy and *Coptis japonica* specific P450 sequences as the “addition” group, which may be Papaveraceae family specific P450 proteins. As a result, we got 155 and 159 unigenes for *M. cordata* and *M. microcarpa* respectively (Figure S5A). The CYP71 family has the most unigenes in both species. We also obtained 12 and 8 unigenes for the “addition” group in *M. cordata* and *M. microcarpa*. The expression levels of the unigenes in the “addition” group were relatively higher than other subfamilies in both species and their expressions in roots were much higher than in other organs. The expression patterns for P450 subfamilies in different organs were different, which was shown in Figure S5B.

### ABC Transporters

Since many studies reported that the alkaloids and other secondary metabolites were transported and accumulated by ABC transporters [24]. Here, we identified the ABC transporters in both *M. cordata* and *M. microcarpa* unigenes using the Arabidopsis ABC transporters as queries [32]. Finally, we identified 190 and 205 ABC transporter like unigenes in *M. cordata* and *M. microcarpa*, which distributed in subfamilies A-G, I and the “others” subfamily according to the classification in Arabidopsis. Among all the subfamilies, the ABCB, ABCC, and ABCG subfamilies contained more than 30 unigenes in both species (Figure S6A). For Arabidopsis ABC transporter genes AT3G55320, AT3G59140, and AT4G19210 in subfamily B, C, and E, we found more than 10 orthologous unigenes for each of them in both *M. cordata* and *M. microcarpa*. Further,we checked the expression of those ABC transporter unigenes in both species, and found that most of them were expressed lowly. As exceptions, the DaGuo_32944 (AT3G62700) expressed very highly in phase II fruit shells and leaves of *M. cordata*; DaGuo_50738, DaGuo_52196 and XiaoGuo_54834, XiaoGuo_59665 (AT4G04770) expressed highly in all samples of both species. The XiaoGuo_26389, XiaoGuo_43491, XiaoGuo_43492, XiaoGuo_55138 (AT5G60790) expressed very highly in all samples of *M. microcarpa* (Supporting File S1). Totally, the fruits of *M. cordata* have higher expression for ABC transporter genes, while the leaves of phase I of *M. microcarpa* have relatively high in most of ABC subfamilies (Figure S6B).

### Differential Gene Expression in Different Tissues and Species

**Figure 4:**
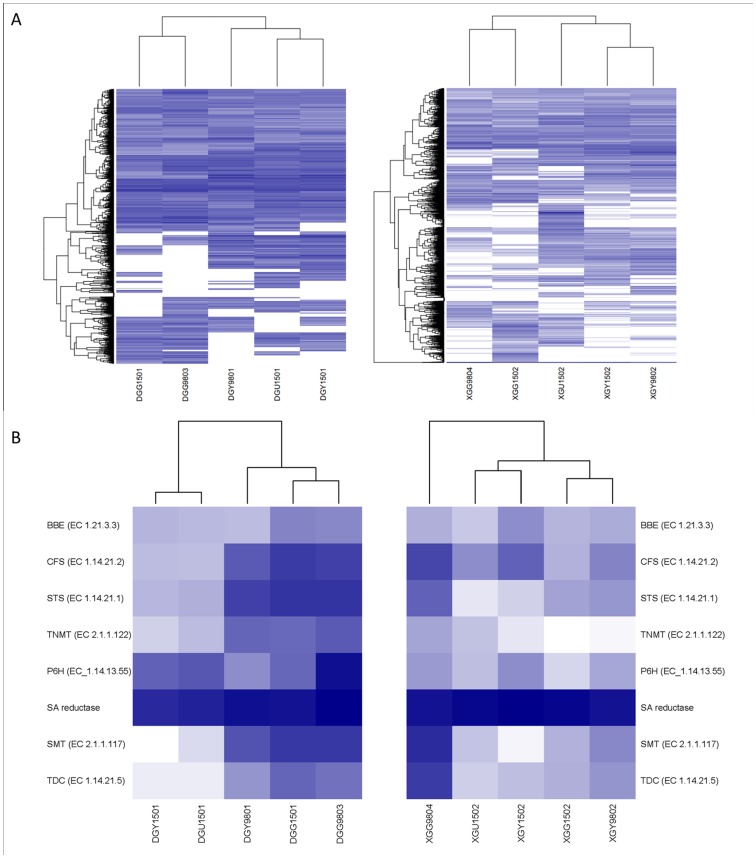
Gene expression cluster for P450 and enzymes in alkaloids pathway. A) Gene expression analysis of P450. RNA-Seq read counts for representative transcripts (rows), expressed as log2 RPKM, were subjected to hierarchical agglomerative clustering based on their expression pattern across *Macleaya* spp.(columns). **B)** BIAs pathway key enzymes differential expression. The expression levels for genes in the pathways and precursor pathways (rows) across the *Macleaya* spp. assayed tissues (columns). The majority of the genes encoding and precursor pathway enzymes are most highly expressed in the stages of roots and fruits. Gene and pathway names correspond to those used in Fig. 1.

Since we sequenced 10 samples for different phases and tissues of *M. cordata* and *M. microcarpa*, we further checked the gene expression in these samples. In both *M. cordata* and *M. microcarpa*, there were several hundred genes displaying differential expression among the sample comparisons (Table 5). Totally, there were 5309 and 5015 unigenes differentially expressed in all these comparisons in *M. cordata* and *M. microcarpa*, respectively. We also compared the gene expression of the same tissues between these two species and found several hundreds of up-regulated and down-regulated genes. Among the more than 27000 common unigenes in both *M. cordata* and *M. microcarpa*, 3895 of them were differentially expressed in these comparisons. After clustering the differentially expressed genes (DEGs) in both species by their expression values, we found that two samples from the same tissues of different times, such as DGG9803 and DGG1501, DGY9801 and DGY1501, XGG9804 and XGG1502, XGY9802 and XGY1502, have similar DEGs (Figure 4A).

**Table 5:** Differential expression Genes(DEGs) statistical result in different organs and species.

Group	Category	Samples	Up-regulated genes	Down-regulated genes
MC vs. MC	Phase I vs. Phase I	DGY9801_vs_DGG9803	501	339
	Phase II vs. Phase II	DGY1501_vs_DGU1501	277	479
		DGG1501_vs_DGY1501	936	166
		DGG1501_vs_DGU1501	795	455
	Phase I vs. Phase II	DGG9803_vs_DGG1501	207	899
		DGY9801_vs_DGY1501	492	175
MM vs. MM	Phase I vs. Phase I	XGY9802_vs_XGG9804	297	514
	Phase II vs. Phase II	XGY1502_vs_XGU1502	732	355
		XGG1502_vs_XGY1502	526	255
		XGG1502_vs_XGU1502	768	189
	Phase I vs. Phase II	XGG9804_vs_XGG1502	158	250
		XGY9802_vs_XGY1502	410	371
MC vs. MM	Phase I vs. Phase I	DGG9803_vs_XGG9804	312	534
		DGY9801_vs_XGY9802	449	613
	Phase II vs. Phase II	DGG1501_vs_XGG1502	331	346
		DGY1501_vs_XGY1502	245	485
		DGU1501_vs_XGU1502	466	142
*M. cordata* vs. *M. microcarpa*			26	55
Phase I vs. Phase II in *M. cordata*			32	23
Phase I vs. Phase II in *M. microcarpa*			13	5
Leaves vs. roots in *M. cordata*			186	67
Leaves vs. roots in *M. microcarpa*			161	19

To compare the results from the 10 samples of different tissues, phases and species, we summarized the common DEGs for the following comparisons: 1) *M. cordata* vs. *M. microcarpa*, including 5 comparisons; 2) Phase I vs. Phase II in both species, each including 2 comparisons; 3) Leaves vs. roots in both species, each including 2 comparisons. The numbers of common DEGs of these comparisons were listed in Table 5. We have checked the annotations of these genes (Supporting File S2). Although most of them had significantly similar proteins in UniProt, most of these hit proteins were “Putative uncharacterized protein” without further function annotations. The 26 highly expressed genes in *M. cordata* than in *M. microcarpa* includes genes encoded glutathione peroxidase, ribulose-phosphate 3-epimerase, disulfide-isomerase like protein, and ubiquitin fusion protein. Genes highly expressed in *M. microcarpa* than in *M. cordata* contains several heat shock proteins and some enzymes (aldolase, kinase, aminotransferase, and phosphatase). In *M. cordata*, we found that several P450 genes were highly expressed in phase II than in phase I. Also most of these genes with annotations highly expressed in phase I of *M. cordata* were enzymes including oxidase, kinase, reductase, and geranylgeranyl pyrophosphate synthase (GGPPS), which is an important enzyme in the mevalonate pathway of Terpenoid backbone biosynthesis.

### Expression of Genes in Alkaloid Biosynthesis Pathways

Isoquinoline alkaloids are a class of important secondary metabolites in *Macleaya* spp. In our annotated *M. cordata* and *M. microcarpa* transcriptome datasets, multiple transcripts encoding almost all known enzymes involved in these alkaloid biosynthesis pathways were identified. Here, we paid attention to the pathways of 4 alkaloids including SA, CHE, PRO and ALL. Since *(S)*-reticuline is the common precursor of these 4 alkaloids, we focused on the pathways from *(S)*-reticuline to downstream alkaloids, which include 7 enzymes. The enzyme EC numbers and their expression values in different samples were shown in Table 6. We clustered and analyzed the expression of the enzyme genes in the pathway by samples in both species (Figure 4B). In *M. cordata*, the enzyme genes were highly expressed in roots of both phases (DGG9803 and DGG1501), among which the RPKM values of several enzymes were more than 500. Enzymes in the leaves of phase I (DGY9801) also had relatively high expression. However, Enzymes in the fruits and leaves of phase II (DGU1501 and DGY1501) were expressed at very low levels. The RPKM values of most enzymes in these two samples were about 10, which were much lower than those of other samples. There was a different situation in the *M. microcarpa*. In *M. microcarpa*, the phase I root had the highest expression, followed by the two leaves samples. The phase II root and fruit shells had the lowest enzyme expression, but they were also higher than those of *M. cordata*. These results suggested that *M. cordata* and *M. microcarpa* may synthesize alkaloids in different tissues at different times. Since the alkaloid SA will be deoxidated into DHSA by the SA reductase in organism, we also checked its expression. We found that in all the 10 samples of both species, its expression values were always much higher than other enzymes. Among them, the phase I root of *M. cordata* had the highest expression and the phase II leaves and fruits expressed lowest.

**Table 6:** The key enzymes of Isoquinoline alkaloid biosynthesis pathway expression in different samples.

Alkaloids				Enz^*^	DGG9803	DGY9801	DGG1501	DGY1501	DGU1501	XGG9804	XGY9802	XGG1502	XGY1502	XGU1502
				**SAR**	4302.22	2401.74	2213.43	1059.51	1368.02	1424.32	1502.96	1956.62	2339.33	1890.29
				**P6H**	2451.47	37.94	135.81	162.43	219.13	52.37	38.92	12.77	71.01	22.41
				**STS**	736.12	485.2	738.15	8.87	11.67	205.49	55.44	44.12	14.33	8.84
			**SA**	**CFS**	463.17	205.06	566.91	7.67	6.94	419.34	93.55	30.77	216.45	72.02
**ALL**	**CHE**	**PRO**		**BBE**	43.55	7.84	52.41	10.47	8.73	31.22	34.83	28.81	70.96	17.61
				**TNMT**	208.09	146.93	126.78	3.85	7.65	40.47	5.73	4.48	8.58	21.04
				**SMT**	614.72	257.61	634.6	0.8	2.84	760.41	81.82	30.12	6.11	19.82
				**TDC**	90.84	29.29	145.67	1.49	1.54	535.02	58.75	32.24	21.47	15.62

Enz: Enzyme; SAR: SA reductase; other enzymes see figure 1.

### iTRAQ Proteome Analysis of Macleaya spp

The proteome of 8 of the 10 samples used in RNA-Seq were analyzed by LTQ Orbitrap Velos (Thermo Fischer Scientific), which includes Phase I (leaves) and Phase II (roots, leaves and fruit shells). Using a stringent cut-off, 4293 peptides and polypeptides were identified using the public plant protein database, of which 475 non-redundant proteins were represented by two or more peptides. In contrast, 1742/1782 peptides and polypeptides were identified using the *M. cordata* and *M. microcarpa* unigenes assembled from RNA-Seq results, of which 1031/1031 non-redundant proteins were represented by two more peptides, respectively. Using the concatenated target-decoy database search strategy as detailed by Elias and Gygi [33], a 0% rate of false positives was estimated after searching the concatenated decoy database and within the limits set by the Paris guidelines for publication of proteomic data (http://www.mcponline.org/misc/ParisReport_Final.dtl). The complete list of proteins identified by proteome analysis was listed in Supporting File S3.

**Figure 5:**
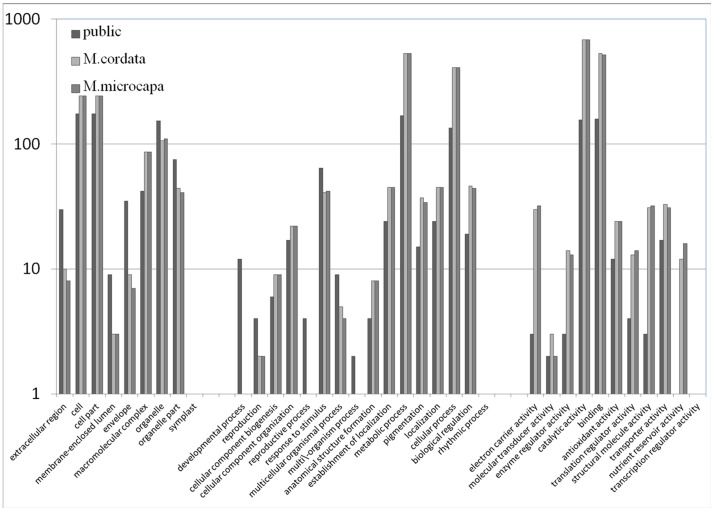
The GO analysis of the iTRAQ identified proteins from different databases. The results from three databases: the public databases and the assembled *M. cordata*, *M. microcarpa* database by BLAST.

Proteins searched by LC-MS/MS peptides in unigenes encoded proteins and public plant proteins were classified into functional categories based on their putative roles in Gene Ontology separately (Figure 5). We divided our results into 3 parts, which were proteins hit in public plant protein database, proteins identified from the unigenes of *M. cordata* and *M. microcarpa*. Based on the results, we found that the GO annotations were quite similar between *M. cordata* and *M. microcarpa*. In the biological process category, the most abundant sub-category is the metabolic process, which represented more than 50% of all identified proteins and included enzymes involved in the isoquinoline alkaloid biosynthesis pathways. The cellular component category of identified proteins includes the cell, cell part and some other organelles. The molecular function category of identified proteins was classified into several main parts. The most enriched sub-categories were the “catalytic activity”, “binding proteins” and “transporters”, which are quite important to our main focus of the alkaloids biosynthesis and accumulation.

We also used the SignalP 4.0 Server (http://www.cbs.dtu.dk/services/SignalP) [34] to predict the signal peptide in the identified proteins. We obtained 18 proteins with predicted signal peptide among proteins identified in public database and 199/184 proteins with signal peptide among proteins identified in *M. cordata* and *M. microcarpa* unigenes. Mapping of the transmembrane domains (TMD) for the identified proteins was conducted using the TMHMM 2.0 program based on transmembrane hidden Markov model (http://www.cbs.dtu.dk/services/TMHMM). Through the prediction, only 240 proteins have more than 1 TMDs. These suggest that our identified proteins are mainly hydrophilic proteins without TMDs which is consistent with the results of SignalP (see Supporting File S4 and S5).

### Differentially Expressed Proteins and their Integration with Transcriptome Data

Furthermore, the differentially expressed proteins (DEPs) by sample comparisons were checked. We are interested in the following four pairs of comparisons: leaves and fruits in phase II of both species (DGU1501 vs. DGY1501 and XGU1502 vs. XGY1502), and leaves in both phases of the same species (DGY1501 vs. DGY9801 and XGY1502 vs. XGY9802), which were chosen considering the time and tissue. According to the public plant database, there were 128 DEPs between leaves of phase I and phase II in *M. cordata* and 109 DEPs between leaves of phase I and phase II in *M. microcarpa*. According to the assembled unigene databases of two species, there were 243 DEPs between leaves of phase I and phase II in *M. cordata* and 240 DEPs between leaves of phase I and phase II in *M. microcarpa*. Comparison between fruits and leaves was based on the relatively special phenomenon of the alkaloids accumulation levels. According to the public plant database, there are 122 DEPs and 117 DEPs between phase II leaves and fruits in *M. microcarpa* and *M. cordata*, respectively. According to the assembled unigene databases of two species, there were 296 DEPs and 186 DEPs between leaves and fruits in *M. microcarpa* and *M. cordata*, respectively (see Table 7). Summarily, there were 1083 proteins differentially expressed among different groups of sample comparisons (overlap in different groups and the detailed differential expression can see in Supporting File S3). Among them we identified the vacuolar protein sorting 29 [Zea Mays], tubulin alpha chain [Ricinus communis], secreted alpha-amylase [Malus × domestica], latex plastidic aldolase-like protein [Hevea brasiliensis] and seed storage protein [Juglans regia] etc. The latex plastidic aldolase-like protein which is mainly for the storage of latex in *Hevea brasiliensis* is similar to the laticifers special proteins (MLP) and we found that it was significantly up-regulated in the roots and the leaves of Phase I. These results show that the two organs maybe possible alkaloids storage places. According to the two phase comparison, the secreted proteins such as the secreted alpha-amylase is significantly up-regulated in phase I and the storage related proteins such as seed storage protein and vacuolar protein sorting 29 are relatively higher in phase II. These proteins may help the accumulation and transporter of the characteristic alkaloids and their relatively higher expression partly describe the function of the whole plant.

**Table 7:** Integrated expressions of unigenes and proteins in two databases.

Unigene database					
	Identified Proteins			Unigene	Integrated
	Total difference	Up-regulated	Down-regulated	Total difference	Co-expression
XGU1502-XGY1502	296	190	106	103	76
XGY9802-XGY1502	240	115	125	61	51
DGU1501-DGY1501	186	67	119	89	69
DGY9801-DGY1501	243	64	179	102	42
**Public database**					
	**Identified Proteins**			**Unigene**	**Integrated**
	**Total difference**	**Up-regulated**	**Down-regulated**	**Total difference**	**Co-expression**
XGU1502-XGY1502	122	84	38	48	36
XGY9802-XGY1502	109	40	69	26	17
DGU1501-DGY1501	117	22	95	55	40
DGY9801-DGY1501	128	30	98	47	27

Since we performed both the transcriptome and proteome analyses for samples of *M. cordata* and *M. microcarpa*, it is necessary for integrating these data for combined analysis. For the integration analysis, we focused on the above four pairs of comparisons. There were more than 300 proteins identified as DEPs in each sample comparisons including results from the public plant database and unigene database. Since DEGs were selected more strictly, we defined only less half of the DEPs differentially expressed on gene level (Table 7). For those genes differentially expressed on both gene and protein levels, we found that more than 2/3 of them differentially expressed on the same trend on transcript and protein levels. The remaining one third genes differentially expressed in opposite trend, which means high expression on gene level and low expression on protein level or reversely. Similar to the DEGs in transcriptome data, more than half of them were putative proteins without function annotation and many of those with annotations were different kinds of enzymes including synthase, adenylyltransferase, dehydrogenase, peroxidase, kinase, and reductase etc.

### The Ultrastructure of Tissues for Alkaloids Accumulation

**Figure 6:**
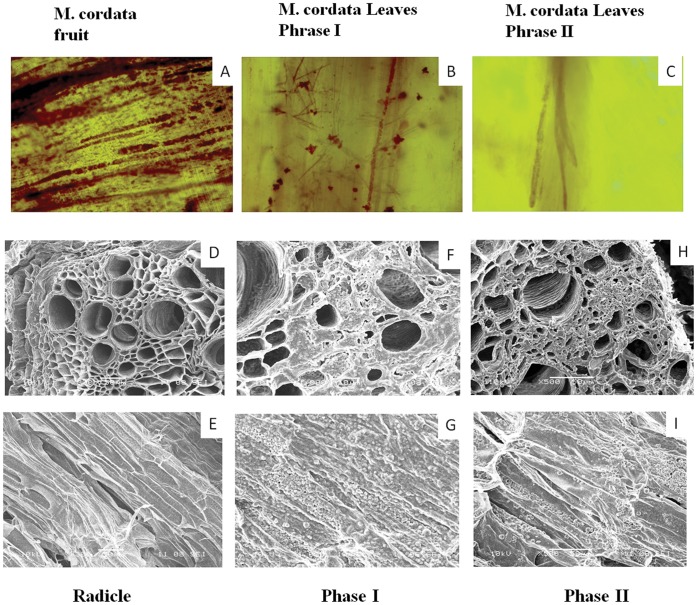
Ultrastructure of *M. cordata* in different developmental stages. A) The results of optical microscope show the alkaloids distribution in different organs in *M. cordata*. **B)** The different stages of roots scan. The radicle stage(D,E), Phase I(F,G) and Phase II(H,I); The ultrastructure is taken by SEM which D,F,H are transverse section and E, G, longitudinal section.

The optical microscope was after Dragendorff staining which could show the alkaloids in salmon pink. The Figure 6 shows the different organs of the *M. cordata*. From the results we can see that: A) fruits were full of the red grains (containing alkaloids); B) the leaves of phase I had some red grains and some similar points, the red points maybe are the laticifers; C) the phase II of the leaves almost had no red grains which suggest no alkaloids at this stage. The SEM result was shown in the Figure 6D–I. The roots from different stages were shown in both transverse and longitudinal section. The phloem of radicle roots (D and E) was quite clear and nothing inside it. However, there were a lot of crystal in the roots of phase I and phase II (F–I), which showed the positive reaction of alkaloids.

### Validation of Expression Pattern by Real-time RT-PCR

**Figure 7:**
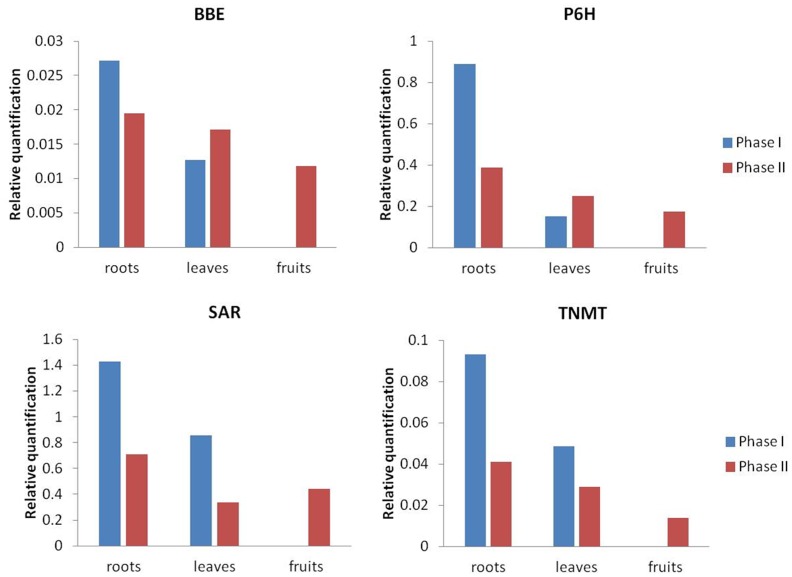
Quantitative RT-PCR validataion. Four candidate unigenes (BBE, P6H, SAR and TNMT) are shown for involved in the BIAs metabolic pathway show differential expression patterns by RT-PCR in three organs were carried out on cDNA prepared from roots (phases I and II), leaves (phases I and II), and fruits shells (phase II) as described in the Material and Methods (phase I show in blue bars and phase II show in red bars). Expression level is relative to ubiquitin and all results represent the mean (± SD) of three experiments.

To validate the changes of gene expression identified by RNA-Seq, qPCR analysis of four enzymes in the BIAs pathway was performed in *M. cordata* (Figure 7). Surprisingly, the relative expression levels of these 4 enzymes in *M. cordata* samples were highly consistent with our transcriptome results. The results showed that P6H expressed highest in phase I roots and lowest in phase I leaves, which were consistent with the transcriptome results. BBE, another key enzyme of BIAs pathway, the expressions were significantly higher in roots of both phases and lower in leaves and fruits. The leaves and fruits of phase II have the lowest TNMT expression, while the phase I roots have the highest expression. The SAR were expression much higher than other enzymes in all organs and also the phase I roots have the highest expression. It was found that the transcription levels of the four enzymes were well related to their translation products during the two developmental stages and different organs. Thus, overall the mRNA and transcripts levels were well correlated.

## Discussion

*Macleaya* spp., a genus of the Papaveraceae family, includes *M. cordata* and *M. microcarpa* is widely spreading around west Europe and Asia. There is a close genus in America called Bocconia spp. which contains about 10 species. Similar to *Macleaya* spp., species in Bocconia spp. also contains alkaloids and have been used medicinally to control mange, lice, and intestinal worms, and to treat ulcers, wounds, and even Leishmaniasis [35]. Both of them have been used more and more wildly not only in human medicinal areas, but also in the stockbreeding and agriculture. The *M. cordata* extract is an exemplification that made of injection which has antibacterial and antipyretic effect of pigs, chickens and fish [3], [4], [6], [8]. We have also developed a new Chinese herbal medicinal veterinary drugs based on the *M. cordata* extract, which is already in the category II new Chinese herbal medicinal veterinary drugs in China (2011 Novel Veterinary Drugs Certificate NO. 33& 34). At the same time, the alkaloids of *M. cordata* have been wildly used as a high efficient, safe, and economical biological pesticide in crop pest control [9], [36]. Furthermore, the *M. cordata* is also a potential energy plant since it has been used as a biogas additive in Germany. Because the alkaloids are the major bio-active components of *Macleaya* spp. plants, improving the alkaloids accumulation in *M. cordata* and *M. microcarpa* will be beneficial to the society. Investigating the gene expression and molecular genetics under the alkaloids biosynthesis is essential for the molecular breeding of *M. cordata*.

### The First and Most Comprehensive Gene Set of Macleaya spp

Although *M. cordata* has been used as a traditional medicine for a long time in the world [1], there are very few molecular studies and sequences for it. Up to date, there are only less than 20 sequences in the GenBank database. In this work, through the RNA-seq deep sequencing technology, we got millions of reads and assembled them into about 70,000 unigenes in both *M. cordata* and *M. microcarpa*. It is the first and most comprehensive gene set of *Macleaya* spp. plant. It will be a useful gene resource for the molecular and genetic studies of plants of Macleaya and Papaveraceae. The EST sequences comparison with the assembled sequences also proved as a proof of our assembly results and gave us more information about the expression. The total of 395 EST sequences of *M. cordata* leaves were already submitted to Genbank with the accession number from JK752513 to JK752907. The transcriptome data and unigenes are necessary basic resources for further genome sequencing, annotation and the discovery and application of key enzymes in the alkaloid biosynthesis.

### The Gene Expression in *M. cordata* and *M. microcarpa*

In this study, we studied both of *M. cordata* and *M. microcarpa* on transcriptome, proteome and alkaloids metabolism profiling. We found that *M. cordata* and *M. microcarpa* were two species of *Macleaya* spp. plants with differences in the patterns of gene and protein expression, alkaloids biosynthesis and accumulation. Although we sampled and sequenced the same organs at the same time and also assembled the RNA-Seq reads using the same strategy for both species, we got small difference in the unigene numbers (69367 for *M. cordata* vs. 78255 for *M. microcarpa*). Since the unigenes assembled from transcriptome data included some alternative splicing transcripts for genes, the more unigenes obtained from transcriptome data of *M. microcarpa* may suggest it has more alternative splicing transcripts. The closed genetic relationship of these two species indicates that they should be similar at the genome gene pools level. The COG, KEGG and GO annotations for unigenes of both species were highly similar (Figure S2, Table 3, Figure S4).

There were different gene expression patterns for different organs and time phases. By comparing, we found several hundred DEGs between two samples of the same species or the same organ of different species (Table 5). We also checked and found some common DEGs for 3 kinds of comparisons between species, phases and organs. Although most of these common DEGs encoded unknown proteins, we still observed that P450 and other enzymes were rich in them. We focused on the 4 alkaloids biosynthesis and found that different organs had different accumulation patterns for the 4 alkaloids. However, we also found hundreds of DEGs in the comparison between same organs of phase I and II in the same species, which have similar pattern for the 4 alkaloid levels. These suggest that genes affected the alkaloids accumulation maybe not in the DEG lists or our strategies for DEGs were too strict.

According to the KEGG pathway results, we noticed the Methane metabolism [PATH: ko00680] pathway as a significantly enriched pathway in both species. This suggested *M. cordata* and *M. microcarpa* have functions in the methane production. In fact, the *M. cordata* has been used as a biogas addictive in Germany as sensoPower® (www.sensopower.com). As described in the description of the production, with *M. cordata* extract, the complete organic degradation is speeding with less transformation losses and the efficiency of the system is significantly increased.

### The Correlation of Alkaloids Accumulation and the Gene Expression of their Biosynthesis Enzymes

Using the analytical method we proposed previously [10], we analyzed the alkaloids in the *Macleaya* spp. plants. We found interesting opposite alkaloids accumulation in the same organs in *M. cordata* and *M. microcarpa*, which SA and CHE were high in *M. cordata* fruits and *M. microcarpa* leaves, while PRO and ALL were high in *M. cordata* leaves and *M. microcarpa* fruits (Table 1). The PRO and ALL are the precursors of the SA and CHE, respectively (Figure 1) and the accumulation levels of alkaloids SA and CHE in all samples of both species were similar. These suggested that the activities of enzymes from *(S)*-Reticuline to PRO (BBE, CFS, STS, TNMT, and MSH) were similar to the enzymes from *(S)*-Reticuline to ALL (BBE, SMT, TDC, TNMT, and MSH) and activities of enzymes from PRO to SA were similar to those from ALL to CHE (Figure 1). In fact, 3 of the 5 enzymes are identical in these two pathways to PRO and ALL. We also observed similar gene expression pattern of the other two enzymes in each pathway. Although most of the enzymes in the steps from ALL to CHE and from PRO to SA were not identified currently, we inferred that the enzyme expression of these two pathways should be similar since their start and end production have similar trend. Combining with the gene expression of those alkaloids biosynthesis enzymes, we found some similar trends and different features in *M. cordata* and *M. microcarpa*. Furthermore, we made some inferences for the time and tissues of these alkaloids biosynthesis.

*M. cordata* had some special features by combining the enzyme expressions and alkaloids accumulations. From the enzyme gene expression results in Table 6, we noticed that all these enzyme genes in the 4 alkaloids biosynthesis except for P6H were expressed very low in the fruits and leaves of *M. cordata* in phase II (DGU1501 and DGY1501). These indicated that there were only few PRO and ALL biosynthesis in leaves and fruits after the fruit mature (phase II). The analytical results also show that there was lower PRO and ALL accumulation in fruits (Table 1). P6H is an enzyme of the first and key step in the pathway from PRO to SA. The protopine-type alkaloids and the benzophenanthridine total quantity is up to 5–7% of plant biological quantity. The protopine-type alkaloids are also the benzophenanthridine precursor and the substrate of P6H. The high expression of P6H in the phase I roots and phase II fruits indicated the high synthesis from PRO to SA. Meanwhile, the expression of SA reductase was lower in fruits than in other samples, which also benefits for the SA accumulation. The hydroxylation of Protopine generate the 6-hydroxyprotopine is easy to be hydrolysed into hypone intermediate product 1 and then with the intermolecular cascade condensation reaction reset to the dihydrosanguinarine. Thus, we detected the highest levels of SA in the fruits of phase II *M. cordata*.

All enzymes for PRO and ALL biosynthesis were highly expressed in the roots of both phases in *M. cordata* (Table 1), which suggested substantial biosynthesis of PRO and ALL in roots. The high levels of PRO and ALL detected in roots also supported this. We concluded that the root was the main organ for the biosynthesis of these two alkaloids. The expressions of these genes in leaves in phase I (DGY9801) were higher than fruit shells and phase II leaves, which indicated that leaves before flowering also synthesized some of PRO and ALL. We also detected high levels of PRO and ALL in leaves of both phases, and we deduced that some of these two alkaloids were transported from roots to leaves.

It is surprising that we detected very high expression of SA reductase. Since we used the SA reductase protein sequence of *Eschscholzia californica* (GenBank: ADE41047) to identify this gene in *Macleaya* plants, we found several unigenes were highly similar to the query sequence and they may be alternative splicing transcripts of this gene or duplicated genes. We merged the expression levels of these highly similar hits as the expression of SA reductase in *M. cordata*. This may explain its high expression partly. Another reason may be that the SA reductase is ineffective in catalyzing SA to DHSA, so it needs large amount of gene expression to make the balance. In fact, in our previous study, we detected that the DHSA is much lower than SA in all organs of samples before flowering [10].

The enzyme expression and alkaloid levels in *M. microcarpa* were different from those in *M. cordata* (Table 6). The gene expressions of enzymes in *M. microcarpa* were always lower than those in *M. cordata*. The gene expressions in 3 samples of phase II were similar and generally lower than phase I. These indicated that there were fewer alkaloids biosynthesis after fruits mature than the time of growing in *M. microcarpa*. The P6H gene expression was relatively high in phase I roots and phase II leaves, while it was relatively lower in fruits compared to other tissues, which is much different from *M. cordata*. Meanwhile, the level of SA was very low in fruits and high in leaves of both phases. This suggested that in phase I (before flowering) the SA may be synthesized in roots and leaves, and then be transported to leaves. After fruits mature, the *M. microcarpa* roots had very low P6H expression and SA biosynthesis, with the SA maybe being mainly synthesized in leaves in phase II. In the *M. microcarpa* roots, all the enzymes in the 4 alkaloids biosynthesis expressed higher in phase I than the roots of phase II. This means the events of all the alkaloids biosynthesis were more active in the phase before flowering than fruits mature. The low detectable SA and CHE in phase I roots maybe because they were transported from roots to leaves.

**Figure 8:**
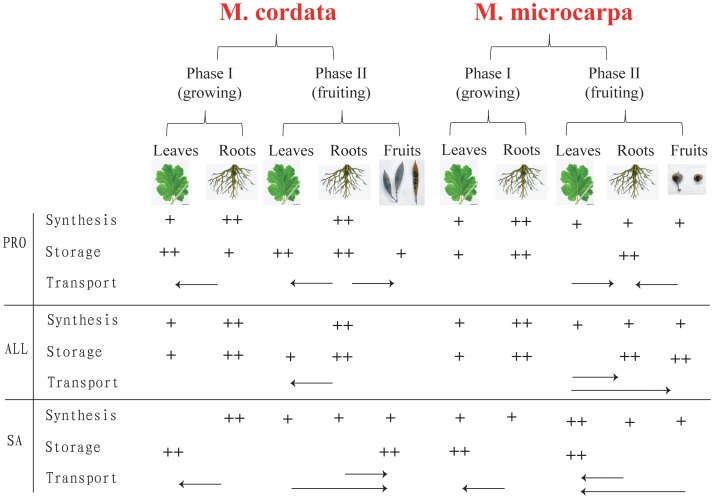
The biosynthesis, storage and transport of three main alkaloids (PRO, ALL and SA) in different samples. The number of plus means the relative abundance and the arrow shows the possible transport direction. All the results are based on the transcriptome and metabolism profiling results.

Based on our results, we inferred there were differences for the alkaloids biosynthesis, storage and transport in *M. cordata* and *M. microcarpa*. In both *M. cordata* and *M. microcarpa*, the root was the main biosynthesis organ and leaf was the minor organ before flowering in phase I. After fruits mature, root was almost the only one organ for alkaloids biosynthesis in *M. cordata*. While in *M. microcarpa*, leaf was the major organ for alkaloids biosynthesis, roots and fruits were the minor organs. The alkaloids transport was also different in *M. cordata* and *M. microcarpa* since their different accumulations. Finally, we summarized the biosynthesis, storage and transport of PRO, ALL and SA of both species in the Figure 8. Since it is still unknown for the steps from ALL to CHE yet, we can’t infer the biosynthesis and transport of CHE and didn’t summarize it in this figure.

Relatively little attention has been focused on the relationship between benzylisoquinoline alkaloid and primary metabolic pathways. Certainly, an important consideration for any metabolic engineering strategy is the availability of primary metabolic precursors. The role of Tyr as the precursor to the benzylisoquinoline alkaloid pathway suggests that the regulation of the shikimate and aromatic amino acid pathways might contribute significantly to the ability of *Macleaya* spp. plant to produce substantial quantities of products, such as SA.

### A Comparison of the DEGs in Transcriptome to the DEPs in Proteome

We combined the DEGs from transcriptome data and DEPs from proteome data for samples from different phases, species (*M. cordata* or *M. microcarpa*) and crossover to better understand the differences. The interest lies with those mRNAs and proteins that share a common direction. The results (Table 7) show directional correlation for some mRNAs and proteins (for example, latex plastidic aldolase-like protein, calmodulin, catharanthus roseus peroxidase 2a and ATP synthase beta subunit), while others are inversely correlated (for example, immunophilin, seed storage protein, DHAR class glutathione transferase DHAR3 and histone h2b ). The mRNAs and their respective proteins exhibiting discordant directional change following different phases indicate that particular pathways and processes are regulated. Differences in the direction of change between transcript and protein are likely related to our single sampling, as the growth cycle need to divided more carefully and meticulous. Furthermore, the regulatory mechanisms of the genome and proteome are complex, which both turnover and stability of mRNA levels are important for the translation of mRNA into protein [37]. For example, if the mRNA is decreased, but the protein is increased, it is possible that the mRNA has already begun to be degraded. Conversely, if the mRNA is increased, but the protein is decreased, there may be regulation of translational pathways, or increased protein degradation leading to induced transcription. The SA reductase,which reduces SA and related benzophenanthridine alkaloid plays a major role in the detoxification of these alkaloids [38], [39]. Latex plastidic aldolase-like protein which is related to the function of the laticifer was found in our results [40]. The NADPH associated proteins such as the NADPH-cytochrome P450 oxidoreductase (CPR) were very important in our alkaloids biosynthesis. They serve as the electron donor to almost all eukaryotic P450s such as the NMCH, CFS, STS, MSH and P6H (Figure 1). They belong to a small family of diflavin proteins with high structural homology to those of bacterial flavodoxins and to ferredoxin-NADP(+) oxidoreductases. CPR shuttles electrons from NADPH through the FAD and FMN-cofactors into the central heme-group of the P450s [41]. Furthermore, this study included the development of a TRIzol based method to analyze both the transcriptome and the proteome from the same sample, which should reduce disagreements in the gene-protein correlation. Future studies aimed at examining the temporal correlation between the transcriptome and proteome should further provide insights into the processes governing biosynthesis.

### Alkaloids Accumulation and Transport in Macleaya spp

It has been reported that benzylisoquinoline alkaloids were biosynthesized in sieve elements in Opium Poppy since several enzymes were identified in the sieve elements [42], [43] and those alkaloids accumulation were restricted to laticifers, which were found adjacent or proximal to sieve elements of the phloem [44]. In this study, we observed the laticifers being stained in the optical microscope in the leaves of phase I (Figure 6B), which indicated that leaves may be also an important place for alkaloid synthesizes before flowering. Though the fruits have high level of SA alkaloid, they almost have no laticifers, which suggested that the alkaloids may not be synthesized in fruits. In the radicle, the phloem structures which contain the vessels, sieve elements and laticifers, were all empty (Figure 6D, 6E). However, in the roots of adult plants (phase I and II), we observed full of the crystalline particle in laticifers and confirmed that it contained SA. We inferred that those alkaloids crystals were located in laticifers, which we analyzed much higher gene expression of the major latex protein (MLP) genes in the roots of *M. cordata* of phase II. The MLP gene is a laticifer specific gene usually as a marker of laticifer [45]. The difference of alkaloid levels in *M. cordata* and *M. microcarpa* indicates that the biosynthesis and transport of alkaloids in these two species were different. ABC transporters are a kind of transporters involved in the transport of plant secondary metabolites [46]. CjMDR1 in *Coptis japonica* is an ABCB-type ABC transporter and currently it is the only one proved transporter transporting alkaloids. Our analysis of its orthologous genes in both *M. cordata* and *M. microcarpa* showed that the expression of this gene in phase I leaves and phase II fruits have much higher expression than other samples (Table S3). This also suggested it may be responsible for the transport of SA and other alkaloids. The total expression of subtype A, B and C of ABC transporters in fruits of *M. cordata* were much higher than in other organs also suggested that the high alkaloids level in fruits of *M. cordata* were transported, not locally synthesized. In summary, we concluded that although the biosynthesis was an essential step for the alkaloids, their storage and transport were also very important for alkaloids accumulation and increasing production. It is also a direction to improve the alkaloids production in the future studies.

### The Integration of Transcriptome, Proteome and Metabolome Analysis for the Non-model Plants

As the decreasing cost of the deep sequencing, transcriptome analysis is becoming a common analysis for gene expression. Since the genome sequence of *Macleaya* spp. plant is unavailable, we performed transcriptome, proteome and metabolism analysis to study the alkaloids biosynthesis in this study. The induction of SA biosynthesis and supporting metabolism has been reported using a variety of technologies including EST and species-specific microarray analyses to analyze the transcriptome in elicitor-treated opium poppy tissues [25], [47] and the profile of the metabolome [10]. Although these studies provided valuable insights into the *Macleaya* spp. plant molecular biology, the technologies used to generate the various data were limited in terms of the depth of penetration into the transcriptome, proteome and metabolome of *Macleaya* spp. plant. More extensive genomics resources for *Macleaya* spp. plant would improve the downstream identification and discovery of enzymes involved in alkaloid biosynthesis. New sequencing technologies such as Illumina sequencing, and advances in LC MS/MS-based proteomics and bioinformatics, will expand the application of genomics methodologies to a vast array of non-model plants that produce interesting and valuable metabolites.

The *Macleaya cordata* and *Macleaya microcarpa* are traditional anti-virus, inflammation elimination, insecticide herb medicines in China. In recent years, more and more scientists focused on the researches of isoquinoline alkaloid application in the anticancer and rubbing medicine, veterinary drugs, animal feed additive, and biogas energy regulator. Improving the alkaloid levels in *Macleaya* spp. is a key step in the further extensive application of the species. In this study, we carried out transcriptome, proteome and metabolism analysis for 10 samples in both species of different time and organs. We revealed the relationship between the gene expression of alkaloid biosynthesis enzymes and the alkaloids accumulation levels. We conclude that root is the major organ for alkaloids biosynthesis and summarized the alkaloids biosynthesis, storage, and transport for both species. This transcriptome dataset can serve as an important public information platform for molecular breeding, functional genomics, synthetic biology and proteomics studies in *Macleaya* spp. plant and shed light on those none-model plant research using the *de novo* sequence research and focus on the application of their secondary metabolites.

## Materials and Methods

### RNA Sequencing Reads Assembly and Unigene Annotation

Total RNA was extracted from the 10 samples of both species was sequenced by Illumina HiSeq2000 platform and 90 bp paired-end reads were generated. Raw sequence reads data have been deposited in the NCBI Sequence Read Archive with the following identifiers: *M. cordata* transcriptome-[SRA: SRA048772] and *M. microcarpa* transcriptome-[SRA: SRA048780]. We used the Trinity program to assembly high quality reads for each samples [29]. To reduce the sequence redundancy, the transcripts of *M. cordata* and *M. microcarpa* were clustered using TGICL individually. Functional annotations for the assembled unigenes were performed by BLAST similarity search against NCBI nr, COG, GO, KEGG (E-value: 10^−5^) or domain search against Pfam.

### Analysis of Differential Gene Expression

We used the RSEM software [48] to calculate each unigene’s corresponding raw reads number, which is expected by DESeq. With unigene’s reads counts, DEseq was used to generate statistical information such as expression level, fold change, p value and FDR, thus to differentially expressed genes (DEGs). We defined the DEGs with following conditions: 1) fold change greater than 2; 2) Expression level (“baseMean” in terms of DESeq) for one of the two samples greater than 50; 3) p values lower than 0.01 and FDR lower than 0.5. We analyzed differentially expressed genes of the same tissue at different phases, and the different tissues at the same phase. We also compared the gene expression of the same tissues between two species. To analyze DEGs of different species, the double best hit in *M. cordata* and *M. microcarpa* were identified and the expression was compared between these double best hit pairs.

### Identification of Isoquinoline Alkaloid Biosynthetic Enzymes

The sequences of isoquinoline alkaloid biosynthetic enzymes from other plants were downloaded from UniProt database. The assembled unigenes of *M. cordata* and *M. microcarpa* were compared to these sequences respectively using BLASTX with a significant threshold of E-value <10^−10^ and identity >50%. The most similar unigenes were considered as each enzyme’s corresponding unigenes. The sum of these unigenes’ RPKM values was considered as the expression level of these enzymes, then hierarchical clustering of log-transformed expression data was carried out using R. Four selected unigenes of key enzymes with potential roles in Isoquinoline alkaloid biosynthesis pathway were chosen for validation using real time qPCR with gene specific primers designed with Primer3 software (see primer list in Table S4).

### Proteome Analysis

Eight of the 10 samples used in RNA-Seq were used to performed proteome analysis. The sample labeling with iTRAQ reagents was performed exactly as described previously [49]. Labeling was as follows: DGY1501 (113), DGG1501 (114), DGU1501 (115), XGY1502 (116), XGG1502 (117), XGU1502 (118), DGY9801 (119) and XGY9802 (121). The fractionation was performed as described previously [50]. All LC-MS MS experiments were performed on an LTQ Orbitrap Velos (Thermo Fischer Scientific) equipped with a Famos autosampler (LC Packings) and an Agilent 1100 binary high-pressure liquid chromatography (HPLC) pump (Agilent Technologies). Protein identification and quantification for iTRAQ was carried out using the Scaffold Q+ [50]. The search was performed against three databases (NCBI nr whole plant database and proteins encoded by our assembled unigenes in two species). A concatenated target-decoy database search strategy was also employed to estimate the rate of false positives [33].

### HPLC-UV Analysis

A Waters 5125 HPLC system (Waters Corporation, USA) coupled with UV detector was used for quantitative determination of four alkaloids (SA, CHE, PRO, ALL) as our former work describes.

### Scanning Electron Microscopic (SEM) and Optical Microscope Analysis

After sample preparation, the sections were examined and photographed with a JSM-6380LV microscope (JEOL, Akishima, and Tokyo, Japan). For optical microscope sample preparation, bismuth potassium iodide and sulfuric acid were used to show the alkaloids.

Full methods can be seen in Supporting Methods S1 file.

### Data Availability and Accession Numbers

The RNA-seq data are available at NCBI Sequence Read Archive (SRA) with the following identifiers: *M. cordata* transcriptome-[SRA048772] and *M. microcarpa* transcriptome-[SRA048780]. The EST sequences of *M. cordata* are available at Genbank with the accession number from JK752513 to JK752907.

## Supporting Information

Figure S1 **Overview of the length distribution of** ***M. cordata*** **geneset and** ***M. microcarpa*** **geneset.** There are 69367 unigenes for *M. cordata* with an average length of 796 bp and an N50 of 1286 bp. There are 78255 unigenes for *M. microcarpa* with an average length of 740 bp and an N50 of 1208 bp. The size distributions for these two gene sets are similar with each other as show in histogram.
(PDF)

Figure S2 **COG Function Classification of the** ***M. cordata*** **and** ***M. microcarpa*** **transcriptome.** The hit COG clusters were grouped into 24 function categories and the distributions of genes in two species were closely similar to each other.
(PDF)

Figure S3 **Pfam family classification of** ***M. cordata*** **and** ***M. microcarpa*** **transcriptome.** Pfam is comprehensive database for protein functional domain and family annotations. ESTscan software was used to translate the unigenes to proteins and we also used HMMER software to search the Pfam domain profiles. 26565 and 27869 proteins were aligned to 3270 and 3421 Pfam domains/families in *M. cordata* and *M. microcarpa*, respectively. The top 10 most frequently detected domains were identical in both species.
(PDF)

Figure S4 **Gene Ontology (GO) Classification of** ***M. cordata*** **and** ***M. microcarpa*** **transcriptome.** Gene ontology (GO) term assignments to *M. cordata* and *M. microcarpa* unigenes based on significant plant species hits against the UniProt database are summarized into three main GO categories (biological process, cellular component, molecular function).
(PDF)

Figure S5 **P450 gene family distribution and expression cluster.** A) P450 family classification of *M. cordata* and *M. microcarpa* transcriptome; B) P450 family expression level cluster analysis. P450 unigenes classification according to the P450 protein sequences collected from *Arabidopsis*, rice, opium poppy, and *Coptis japonica*. The opium poppy and *Coptis japonica* specific P450 sequences were defined as the “addition” group, which may be Papaveraceae family specific P450 proteins.
(PDF)

Figure S6 **ABC transporter gene family distribution and expression cluster.** A) ABC transporter family classification of *M. cordata* and *M. microcarpa* transcriptome; B) ABC transporter family expression level cluster analysis. 190 and 205 ABC transporter like unigenes were identified in *M. cordata* and *M. microcarpa*, which were distributed in subfamilies A-G, I and the “others” subfamily according to the classification in Arabidopsis.
(PDF)

Table S1 Overview of ten samples transcriptome clean reads after assembly.
(PDF)

Table S2 GO term distribution of unigenes of *M. cordata* and *M. microcarpa.*
(PDF)

Table S3 The ABC Transporter expression level.
(PDF)

Table S4 Primer sequences used in qPCR.
(PDF)

File S1 **The unigenes corresponding to ABC transporters (the expression level is the summation of unigenes RPKM values).**
(XLSX)

File S2 **The co-expression genes between different samples (including up-and down-regulated) and their annotations.** It includes the comparison between 1) *M. cordata* vs. *M. microcarpa* 2) *M. cordata* phase I vs. phase II 3) *M. microcarpa* phase I vs. phase II 4) *M. cordata* roots vs. leaves 5) *M. microcarpa* roots vs. leaves.
(XLSX)

File S3 **Complete list of all proteins identified in** ***M. cordata*** **and** ***M. microcarpa.***
(XLSX)

File S4 **The signal peptide prediction results of all proteins identified in** ***M. cordata*** **and** ***M. microcarpa.***
(DOCX)

File S5 **The transmembrane domains (TMD) results for protein identified by proteome.** The identified proteins were conducted using the TMHMM 2.0 program based on transmembrane hidden Markov model (http://www.cbs.dtu.dk/services/TMHMM).
(DOCX)

Methods S1 **Full methods description.**
(PDF)
