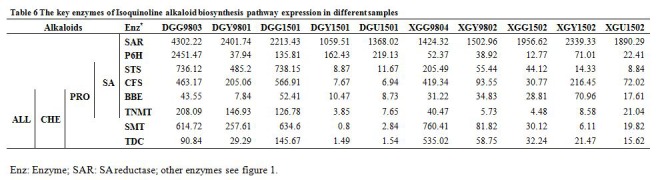# Correction: Integration of Transcriptome, Proteome and Metabolism Data Reveals the Alkaloids Biosynthesis in *Macleaya cordata* and *Macleaya microcarpa*

**DOI:** 10.1371/annotation/6848d2aa-d15f-4632-9074-727b25958da3

**Published:** 2013-09-10

**Authors:** Jianguo Zeng, Yisong Liu, Wei Liu, Xiubing Liu, Fuqing Liu, Peng Huang, Pengcheng Zhu, Jinjun Chen, Mingming Shi, Fang Guo, Pi Cheng, Jing Zeng, Yifang Liao, Jing Gong, Hong-Mei Zhang, Depeng Wang, An-Yuan Guo, Xingyao Xiong

In Table 6, the alkaloid names have been placed incorrectly. Please see the corrected Table 6 here: 

**Figure:**