# Supplementary figures and images for: Integration of Transcriptome, Proteome and Metabolism Data Reveals the Alkaloids Biosynthesis in Macleaya cordata and Macleaya microcarpa
Source: PLoS One. 2013 Jan 9;8(1):e53409. doi: 10.1371/journal.pone.0053409 (PMC3541140; doi:10.1371/journal.pone.0053409)

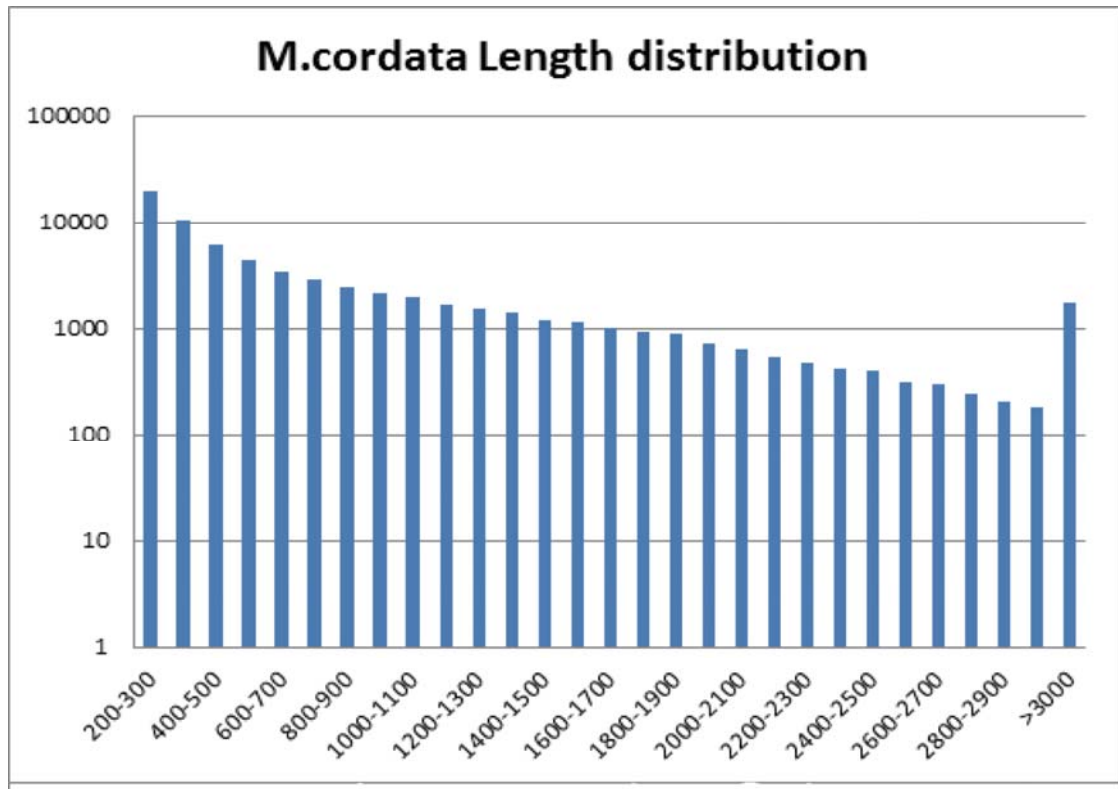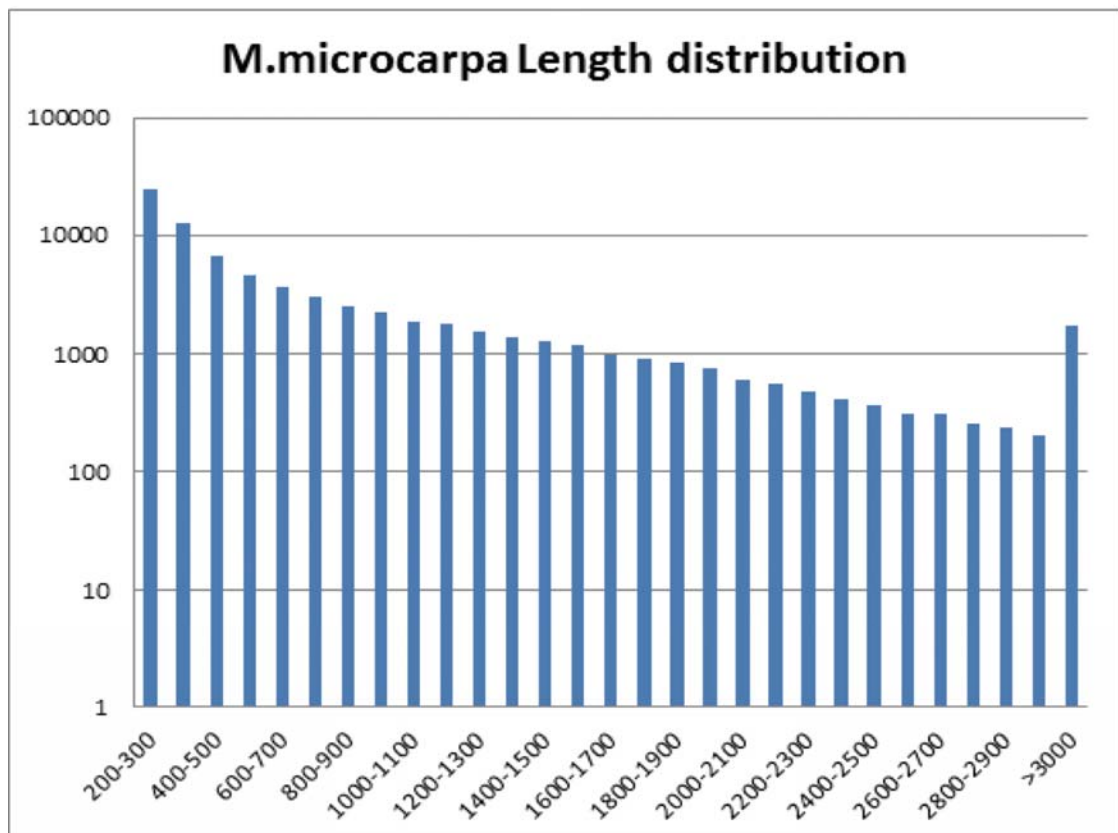

**Figure S1. Overview of the length distribution of M. cordata geneset and M. microcarpa geneset.**

Supplement: Figure S1 — Overview of the length distribution of M. cordata geneset and M. microcarpa geneset. There are 69367 unigenes for M. cordata with an average length of 796 bp and an N50 of 1286 bp. There are 78255 unigenes for M. microcarpa with an average length of 740 bp and an N50 of 1208 bp. The size distributions for these two gene sets are similar with each other as show in histogram. (PDF) [file pone.0053409.s001.pdf]

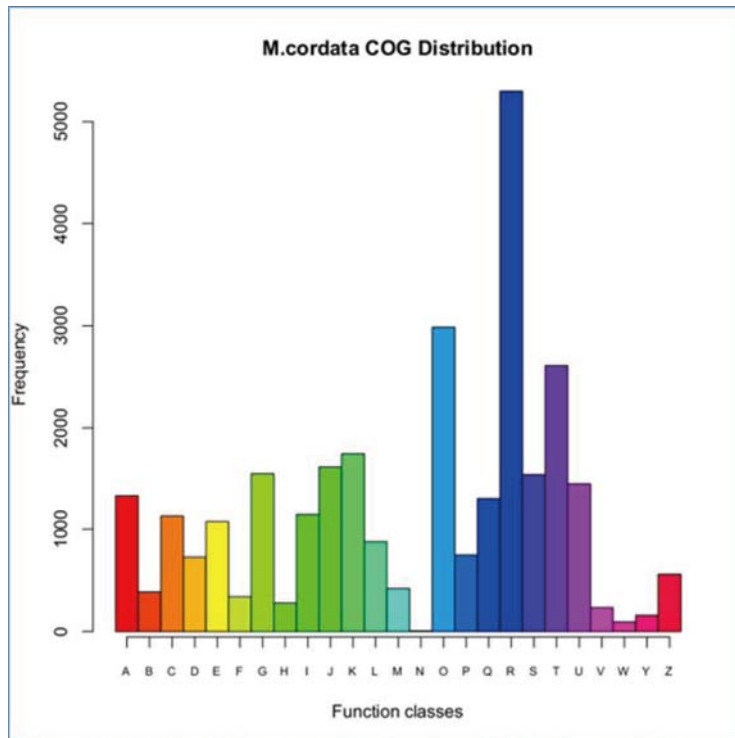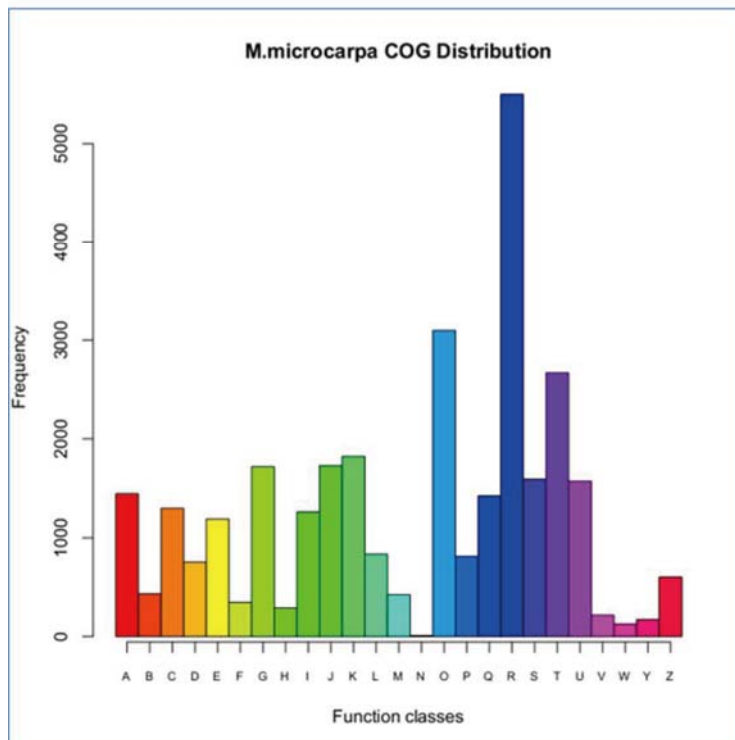

**Figure S2. COG Function Classification of the *M. cordata* and *M. microcarpa* transcriptome.**

Supplement: Figure S2 — COG Function Classification of the M. cordata and M. microcarpa transcriptome. The hit COG clusters were grouped into 24 function categories and the distributions of genes in two species were closely similar to each other. (PDF) [file pone.0053409.s002.pdf]

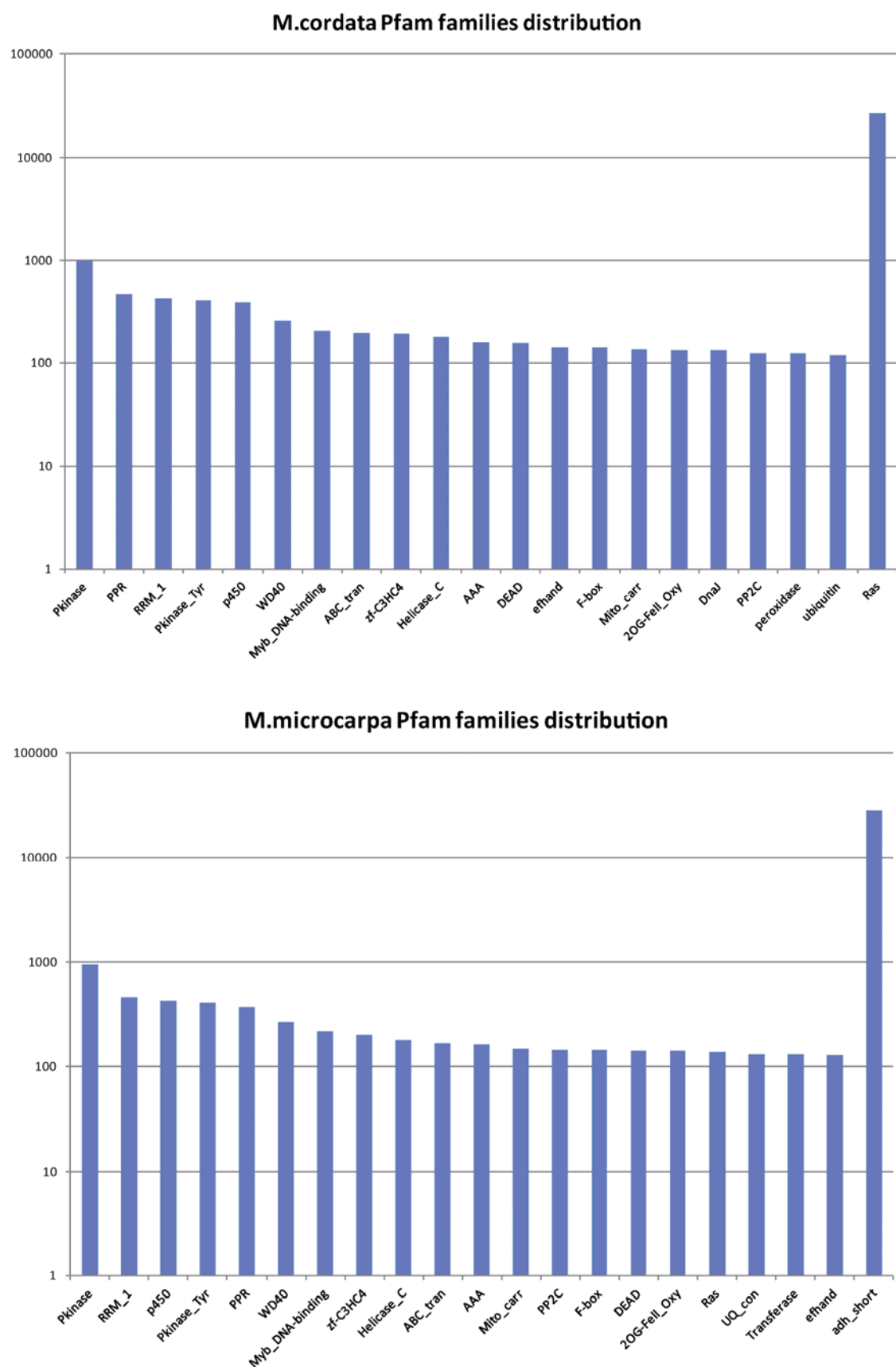

**Figure S3. Pfam family classification of *M. cordata* and *M. microcarpa* transcriptome.**

Supplement: Figure S3 — Pfam family classification of M. cordata and M. microcarpa transcriptome. Pfam is comprehensive database for protein functional domain and family annotations. ESTscan software was used to translate the unigenes to proteins and we also used HMMER software to search the Pfam domain profiles. 26565 and 27869 proteins were aligned to 3270 and 3421 Pfam domains/families in M. cordata and M. microcarpa, respectively. The top 10 most frequently detected domains were identical in both species. (PDF) [file pone.0053409.s003.pdf]

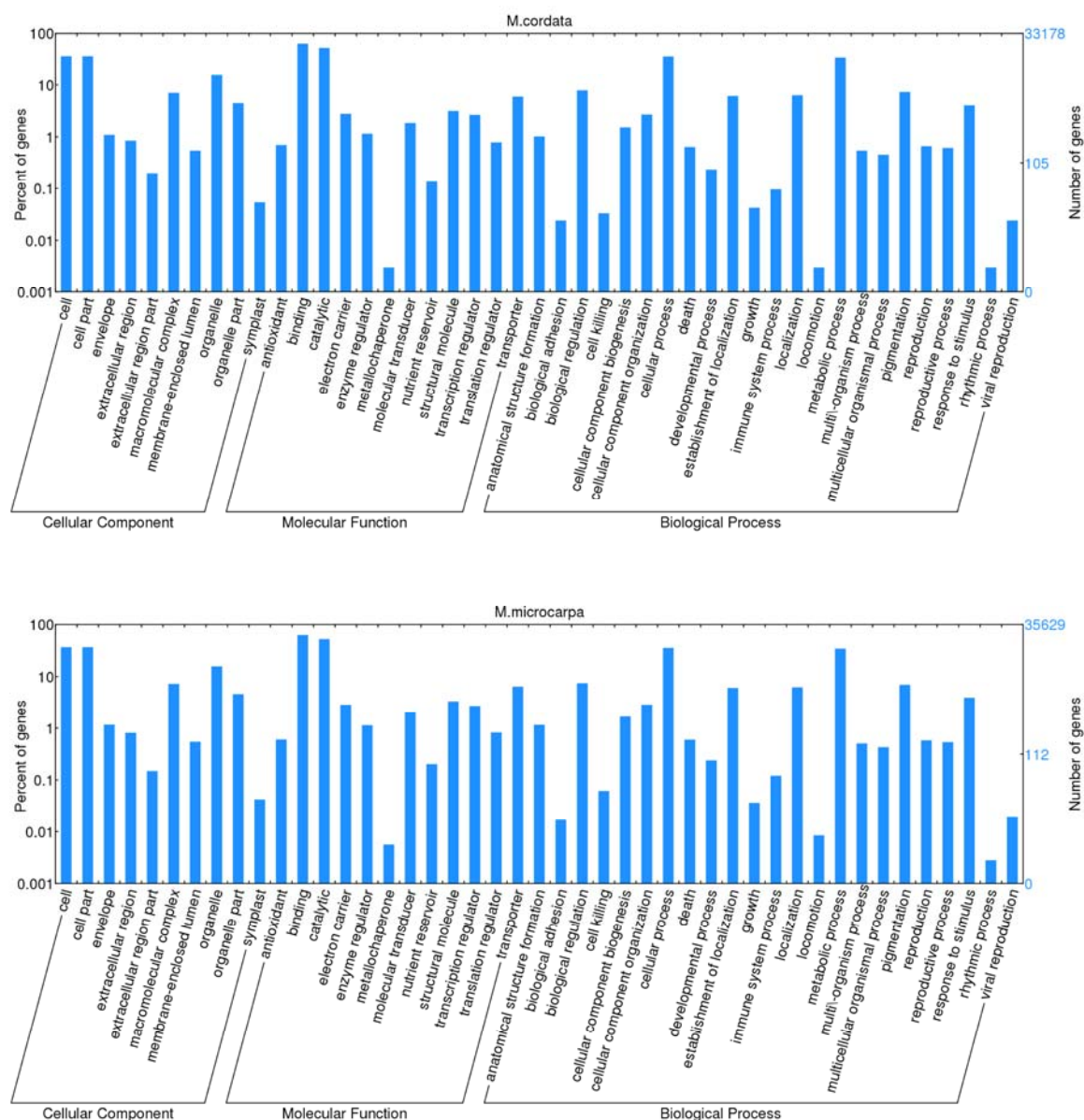

**Figure S4. Gene Ontology (GO) Classification of *M.cordata* and *M.microcarpa* transcriptome.**

Supplement: Figure S4 — Gene Ontology (GO) Classification of M. cordata and M. microcarpa transcriptome. Gene ontology (GO) term assignments to M. cordata and M. microcarpa unigenes based on significant plant species hits against the UniProt database are summarized into three main GO categories (biological process, cellular component, molecular function). (PDF) [file pone.0053409.s004.pdf]

A

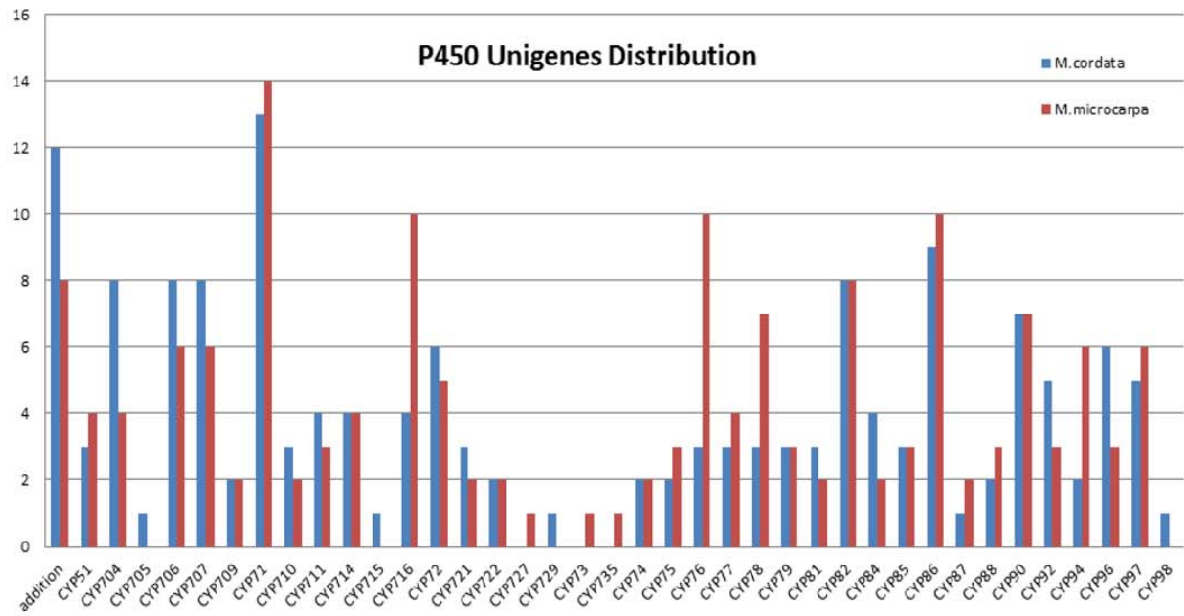

B

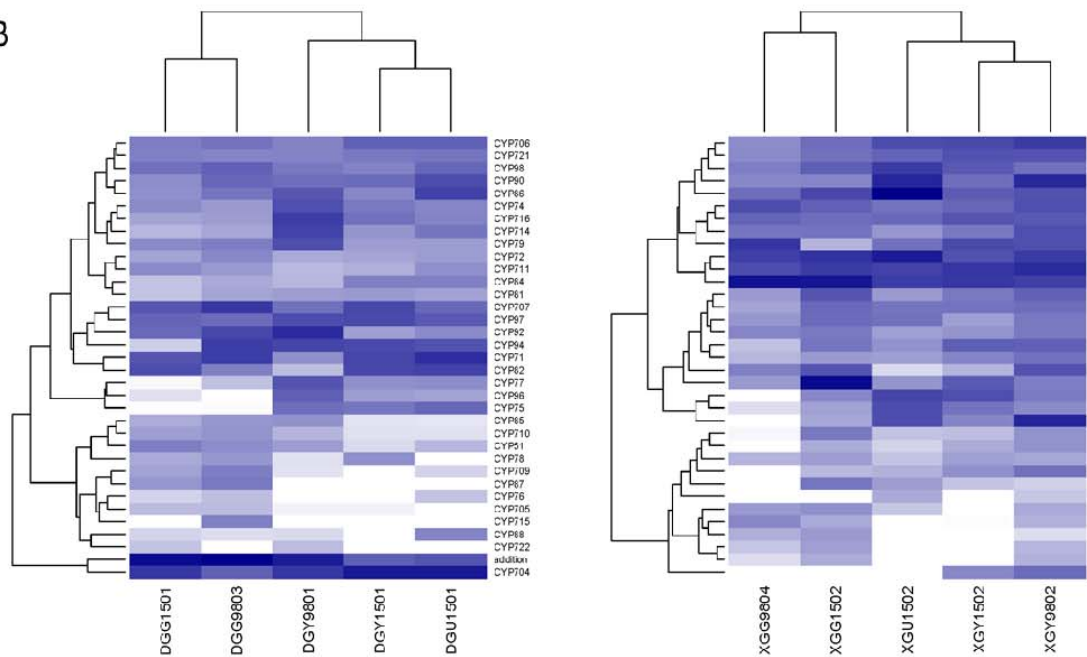

**Figure S5. P450 gene family distribution and expression cluster.**

Supplement: Figure S5 — P450 gene family distribution and expression cluster. A) P450 family classification of M. cordata and M. microcarpa transcriptome; B) P450 family expression level cluster analysis. P450 unigenes classification according to the P450 protein sequences collected from Arabidopsis, rice, opium poppy, and Coptis japonica. The opium poppy and Coptis japonica specific P450 sequences were defined as the “addition” group, which may be Papaveraceae family specific P450 proteins. (PDF) [file pone.0053409.s005.pdf]

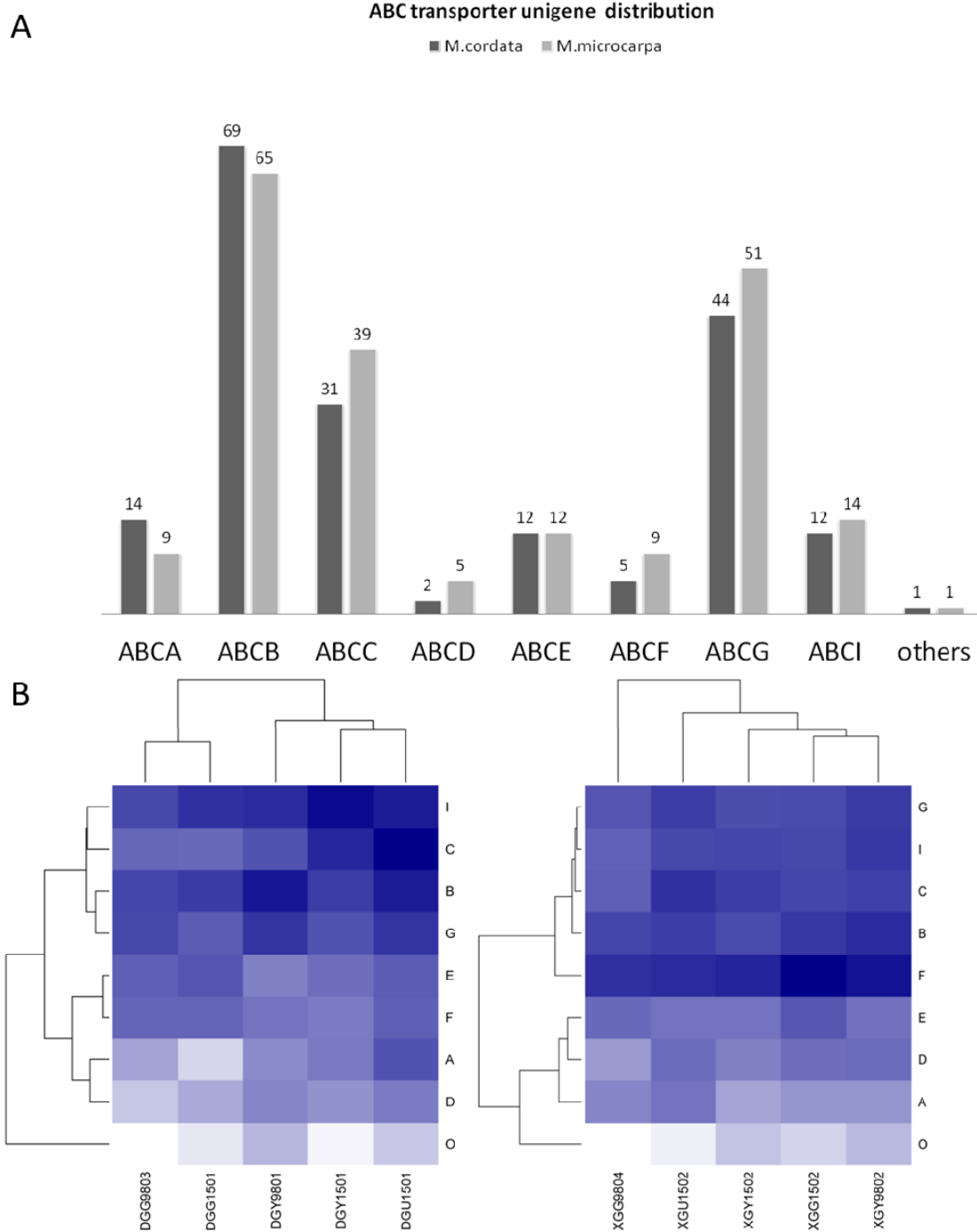

**Figure S6. ABC transporter gene family distribution and expression cluster.**

Supplement: Figure S6 — ABC transporter gene family distribution and expression cluster. A) ABC transporter family classification of M. cordata and M. microcarpa transcriptome; B) ABC transporter family expression level cluster analysis. 190 and 205 ABC transporter like unigenes were identified in M. cordata and M. microcarpa, which were distributed in subfamilies A-G, I and the “others” subfamily according to the classification in Arabidopsis. (PDF) [file pone.0053409.s006.pdf]
