# Supplementary material for: Integration of Transcriptome, Proteome and Metabolism Data Reveals the Alkaloids Biosynthesis in Macleaya cordata and Macleaya microcarpa
Source: PLoS One. 2013 Jan 9;8(1):e53409. doi: 10.1371/journal.pone.0053409 (PMC3541140; doi:10.1371/journal.pone.0053409)
Supplement: Table S1 — Overview of ten samples transcriptome clean reads after assembly. (PDF) [file pone.0053409.s007.pdf]

Table S1 Overview of ten samples transcriptome clean reads after assembly.

Samples	Total Reads	Nucleotides	Q20 percentage	N percentage	GC percentage
DGG1501	13,344,444	1,200,999,960	94.69%	0.01%	44.88%
DGU1501	13,655,556	1,229,000,040	95.23%	0.00%	44.97%
DGY1501	13,511,112	1,216,000,080	94.97%	0.00%	44.28%
DGG9803	13,133,334	1,182,000,060	94.01%	0.00%	45.25%
DGY9801	13,133,334	1,182,000,060	93.99%	0.00%	44.93%
XGG1502	13,844,446	1,246,000,140	94.11%	0.00%	44.97%
XGU1502	13,511,112	1,216,000,080	94.51%	0.00%	44.69%
XGY1502	13,222,224	1,190,000,160	95.07%	0.00%	44.71%
XGG9804	13,133,334	1,182,000,060	93.25%	0.00%	46.60%
XGY9802	13,133,334	1,182,000,060	94.21%	0.00%	44.72%
