# Supplementary material for: Integration of Transcriptome, Proteome and Metabolism Data Reveals the Alkaloids Biosynthesis in Macleaya cordata and Macleaya microcarpa
Source: PLoS One. 2013 Jan 9;8(1):e53409. doi: 10.1371/journal.pone.0053409 (PMC3541140; doi:10.1371/journal.pone.0053409)
Supplement: Table S2 — GO term distribution of unigenes of M. cordata and M. microcarpa. (PDF) [file pone.0053409.s008.pdf]

Tabel S2 GO term distribution of unigenes of M.cordata and M.microcarpa

**M.cordata GO**			**M.microcarpa GO**		
**# of GO**	**GO identity**	**GO term**	**# of GO**	**GO identity**	**GO term**
**Cellular Component:**		**10**	**Cellular Component:**		**10**
12,080	GO:0005623	Cell	13102	GO:0005623	cell
12,080	GO:0044464	cell part	13102	GO:0044464	cell part
5,139	GO:0043226	organelle	5578	GO:0043226	organelle
2,329	GO:0032991	macromolecular complex	2568	GO:0032991	macromolecular complex
1,479	GO:0044422	organelle part	1640	GO:0044422	organelle part
363	GO:0031975	envelope	414	GO:0031975	envelope
280	GO:0005576	extracellular region	289	GO:0005576	extracellular region
180	GO:0031974	membrane-enclosed lumen	194	GO:0031974	membrane-enclosed lumen
66	GO:0044421	extracellular region part	53	GO:0044421	extracellular region part
18	GO:0055044	symplast	15	GO:0055044	symplast
**Biological Process:**		**23**	**Biological Process:**		**23**
11,980	GO:0009987	cellular process	12727	GO:0009987	cellular process
11,394	GO:0008152	metabolic process	12227	GO:0008152	metabolic process
2,599	GO:0065007	biological regulation	2654	GO:0065007	biological regulation
2,420	GO:0043473	Pigmentation	2470	GO:0043473	pigmentation
2,092	GO:0051179	Localization	2214	GO:0051179	localization
2,023	GO:0051234	establishment of localization	2142	GO:0051234	establishment of localization
1,336	GO:0050896	response to stimulus	1397	GO:0050896	response to stimulus
893	GO:0016043	cellular component organization	1020	GO:0016043	cellular component organization
503	GO:0044085	cellular component biogenesis	597	GO:0044085	cellular component biogenesis
339	GO:0010926	anatomical structure formation	410	GO:0010926	anatomical structure formation
220	GO:0000003	Reproduction	213	GO:0016265	death
212	GO:0016265	Death	207	GO:0000003	reproduction
203	GO:0022414	reproductive process	191	GO:0022414	reproductive process
180	GO:0051704	multi\-organism process	180	GO:0051704	multi\-organism process
150	GO:0032501	multicellular organismal process	153	GO:0032501	multicellular organismal process
78	GO:0032502	developmental process	85	GO:0032502	developmental process
32	GO:0002376	immune system process	43	GO:0002376	immune system process
14	GO:0040007	Growth	22	GO:0001906	cell killing
11	GO:0001906	cell killing	13	GO:0040007	growth
8	GO:0022610	biological adhesion	7	GO:0016032	viral reproduction

8	GO:0016032	viral reproduction	6	GO:0022610	biological adhesion
1	GO:0048511	rhythmic process	3	GO:0040011	locomotion
1	GO:0040011	Locomotion	1	GO:0048511	rhythmic process
**Molecular Function:**		**12**	**Molecular Function:**		**12**
21,182	GO:0005488	Binding	22436	GO:0005488	binding
17,463	GO:0003824	catalytic activity	18774	GO:0003824	catalytic activity
1,969	GO:0005215	transporter activity	2275	GO:0005215	transporter activity
1,045	GO:0005198	structural molecule activity	1186	GO:0005198	structural molecule activity
921	GO:0009055	electron carrier activity	1016	GO:0009055	electron carrier activity
881	GO:0030528	transcription regulator activity	965	GO:0030528	transcription regulator activity
613	GO:0060089	molecular transducer activity	733	GO:0060089	molecular transducer activity
380	GO:0030234	enzyme regulator activity	404	GO:0030234	enzyme regulator activity
260	GO:0045182	translation regulator activity	294	GO:0045182	translation regulator activity
231	GO:0016209	antioxidant activity	215	GO:0016209	antioxidant activity
45	GO:0045735	nutrient reservoir activity	72	GO:0045735	nutrient reservoir activity
1	GO:0016530	metallochaperone activity	2	GO:0016530	metallochaperone activity
