# Supplementary material for: Integration of Transcriptome, Proteome and Metabolism Data Reveals the Alkaloids Biosynthesis in Macleaya cordata and Macleaya microcarpa
Source: PLoS One. 2013 Jan 9;8(1):e53409. doi: 10.1371/journal.pone.0053409 (PMC3541140; doi:10.1371/journal.pone.0053409)
Supplement: Table S3 — The ABC Transporter expression level. (PDF) [file pone.0053409.s009.pdf]

Table S3 The ABC Transporter expression level

Protein	DGG9803	DGY9801	DGG1501	DGY1501	DGU1501	XGG9804	XGY9802	XGG1502	XGY1502	XGU1502
CjMDR1	11.37	43.52	3.99	6.42	23.76	0.67	14.88	1.28	4.21	28.36
AtDTX1	15.02	1.48	29.79	0.35	0.55	16.67	7.39	24.84	3.42	12.96
MLP	1041.70	75.62	1073.13	6.38	2229.59	36.57	15.89	111.20	8.54	206.55
