# Supplementary material for: Integration of Transcriptome, Proteome and Metabolism Data Reveals the Alkaloids Biosynthesis in Macleaya cordata and Macleaya microcarpa
Source: PLoS One. 2013 Jan 9;8(1):e53409. doi: 10.1371/journal.pone.0053409 (PMC3541140; doi:10.1371/journal.pone.0053409)
Supplement: Table S4 — Primer sequences used in qPCR. (PDF) [file pone.0053409.s010.pdf]

Table S4 Primer sequences used in qPCR

No.	***gene name***	***Forward primer (5'-3')***	***Reverse primer (5'-3')***
1	BBE	GAAGGGTGTATCAGCGGTGT	TTTCCTCCACGTCTCCATTC
2	P6H	GGAGGCCGTCAATTACAAGA	CCAGCCCTCAAGTATGGTGT
3	SAR	CCTTGAACAGTCTTGGAAACG	GCAACATCTGCCCTAGCAAT
4	TNMT	TGCATTGGAGGGTATGAACA	TGAAACGCAGCAATTTGAAC
