# Supplementary material for: Integration of Transcriptome, Proteome and Metabolism Data Reveals the Alkaloids Biosynthesis in Macleaya cordata and Macleaya microcarpa
Source: PLoS One. 2013 Jan 9;8(1):e53409. doi: 10.1371/journal.pone.0053409 (PMC3541140; doi:10.1371/journal.pone.0053409)
Supplement: File S4 — The signal peptide prediction results of all proteins identified in M. cordata and M. microcarpa. (DOCX) [file pone.0053409.s014.docx]

**Public (Signal peptides 31)**

##gff-version 2

##sequence-name source feature start end score N/A ?

## ---------------------------------------------------------

gi_301176645_gb_ADK66339.1_ SignalP-4.0 SIGNAL 1 24 0.915 . . YES

gi_3913786_sp_Q42434.1_BIP_SPIOL SignalP-4.0 SIGNAL 1 28 0.877 . . YES

gi_187453117_emb_CAP72489.1_ SignalP-4.0 SIGNAL 1 31 0.636 . . YES

gi_225456481_ref_XP_002280858.1_ SignalP-4.0 SIGNAL 1 23 0.891 . . YES

gi_219908399_emb_CAX03474.1_ SignalP-4.0 SIGNAL 1 22 0.882 . . YES

gi_116783314_gb_ABK22887.1_ SignalP-4.0 SIGNAL 1 20 0.499 . . YES

gi_219908431_emb_CAX03490.1_ SignalP-4.0 SIGNAL 1 30 0.836 . . YES

gi_115477084_ref_NP_001062138.1_ SignalP-4.0 SIGNAL 1 28 0.894 . . YES

gi_222850891_gb_EEE88438.1_ SignalP-4.0 SIGNAL 1 20 0.868 . . YES

gi_1223922_gb_AAA92063.1_ SignalP-4.0 SIGNAL 1 22 0.876 . . YES

gi_118482340_gb_ABK93094.1_ SignalP-4.0 SIGNAL 1 22 0.808 . . YES

gi_224115794_ref_XP_002317126.1_ SignalP-4.0 SIGNAL 1 24 0.871 . . YES

gi_161375756_gb_ABX71220.1_ SignalP-4.0 SIGNAL 1 29 0.856 . . YES

gi_126633570_emb_CAM55750.1_ SignalP-4.0 SIGNAL 1 31 0.869 . . YES

gi_147795432_emb_CAN77106.1_ SignalP-4.0 SIGNAL 1 24 0.925 . . YES

gi_85543776_gb_ABC71784.1_ SignalP-4.0 SIGNAL 1 18 0.535 . . YES

gi_115461208_ref_NP_001054204.1_ SignalP-4.0 SIGNAL 1 23 0.672 . . YES

gi_1419088_emb_CAA95999.1_ SignalP-4.0 SIGNAL 1 27 0.899 . . YES

gi_11263719_pir__JC7138 SignalP-4.0 SIGNAL 1 25 0.912 . . YES

gi_158998780_gb_ABW86978.1_ SignalP-4.0 SIGNAL 1 24 0.921 . . YES

gi_255550445_ref_XP_002516273.1_ SignalP-4.0 SIGNAL 1 23 0.880 . . YES

gi_113564263_dbj_BAF14606.1_ SignalP-4.0 SIGNAL 1 22 0.910 . . YES

gi_147799465_emb_CAN70603.1_ SignalP-4.0 SIGNAL 1 20 0.811 . . YES

gi_110742767_dbj_BAE99289.1_ SignalP-4.0 SIGNAL 1 29 0.714 . . YES

gi_115435186_ref_NP_001042351.1_ SignalP-4.0 SIGNAL 1 19 0.878 . . YES

gi_222868651_gb_EEF05782.1_ SignalP-4.0 SIGNAL 1 20 0.868 . . YES

gi_147828306_emb_CAN66483.1_ SignalP-4.0 SIGNAL 1 21 0.869 . . YES

gi_194704686_gb_ACF86427.1_ SignalP-4.0 SIGNAL 1 26 0.837 . . YES

gi_11994450_dbj_BAB02452.1_ SignalP-4.0 SIGNAL 1 20 0.540 . . YES

gi_163930094_dbj_BAD42856.2_ SignalP-4.0 SIGNAL 1 28 0.813 . . YES

gi_218195039_gb_EEC77466.1_ SignalP-4.0 SIGNAL 1 24 0.843 . . YES

**M.cordata (Signal peptides 38)**

##gff-version 2

##sequence-name source feature start end score N/A ?

## ---------------------------------------------------------

DaGuo_2130 SignalP-4.0 SIGNAL 1 21 0.548 . . YES

DaGuo_2218 SignalP-4.0 SIGNAL 1 17 0.606 . . YES

DaGuo_5827 SignalP-4.0 SIGNAL 1 27 0.523 . . YES

DaGuo_7122 SignalP-4.0 SIGNAL 1 18 0.477 . . YES

DaGuo_7352 SignalP-4.0 SIGNAL 1 18 0.576 . . YES

DaGuo_17846 SignalP-4.0 SIGNAL 1 26 0.665 . . YES

DaGuo_26184 SignalP-4.0 SIGNAL 1 27 0.482 . . YES

DaGuo_28433 SignalP-4.0 SIGNAL 1 17 0.471 . . YES

DaGuo_29586 SignalP-4.0 SIGNAL 1 26 0.625 . . YES

DaGuo_29733 SignalP-4.0 SIGNAL 1 24 0.585 . . YES

DaGuo_29735 SignalP-4.0 SIGNAL 1 17 0.606 . . YES

DaGuo_29928 SignalP-4.0 SIGNAL 1 18 0.552 . . YES

DaGuo_30091 SignalP-4.0 SIGNAL 1 23 0.451 . . YES

DaGuo_30314 SignalP-4.0 SIGNAL 1 28 0.566 . . YES

DaGuo_30556 SignalP-4.0 SIGNAL 1 18 0.586 . . YES

DaGuo_30795 SignalP-4.0 SIGNAL 1 20 0.573 . . YES

DaGuo_30802 SignalP-4.0 SIGNAL 1 28 0.566 . . YES

DaGuo_30803 SignalP-4.0 SIGNAL 1 28 0.566 . . YES

DaGuo_30866 SignalP-4.0 SIGNAL 1 19 0.602 . . YES

DaGuo_30896 SignalP-4.0 SIGNAL 1 19 0.570 . . YES

DaGuo_31131 SignalP-4.0 SIGNAL 1 24 0.578 . . YES

DaGuo_31449 SignalP-4.0 SIGNAL 1 18 0.477 . . YES

DaGuo_31450 SignalP-4.0 SIGNAL 1 19 0.581 . . YES

DaGuo_31498 SignalP-4.0 SIGNAL 1 27 0.576 . . YES

DaGuo_32620 SignalP-4.0 SIGNAL 1 20 0.457 . . YES

DaGuo_32625 SignalP-4.0 SIGNAL 1 18 0.672 . . YES

DaGuo_32635 SignalP-4.0 SIGNAL 1 18 0.525 . . YES

DaGuo_37522 SignalP-4.0 SIGNAL 1 18 0.523 . . YES

DaGuo_37525 SignalP-4.0 SIGNAL 1 19 0.496 . . YES

DaGuo_37526 SignalP-4.0 SIGNAL 1 17 0.471 . . YES

DaGuo_38366 SignalP-4.0 SIGNAL 1 28 0.651 . . YES

DaGuo_43511 SignalP-4.0 SIGNAL 1 18 0.458 . . YES

DaGuo_53633 SignalP-4.0 SIGNAL 1 23 0.671 . . YES

DaGuo_53638 SignalP-4.0 SIGNAL 1 24 0.657 . . YES

DaGuo_59482 SignalP-4.0 SIGNAL 1 25 0.459 . . YES

DaGuo_60216 SignalP-4.0 SIGNAL 1 17 0.614 . . YES

DaGuo_61449 SignalP-4.0 SIGNAL 1 19 0.568 . . YES

DaGuo_67271 SignalP-4.0 SIGNAL 1 30 0.475 . . YES

**M.microcarpa (Signal peptides 22)**

##gff-version 2

##sequence-name source feature start end score N/A ?

## ---------------------------------------------------------

XiaoGuo_3055 SignalP-4.0 SIGNAL 1 30 0.607 . . YES

XiaoGuo_3439 SignalP-4.0 SIGNAL 1 28 0.485 . . YES

XiaoGuo_3620 SignalP-4.0 SIGNAL 1 36 0.485 . . YES

XiaoGuo_8047 SignalP-4.0 SIGNAL 1 26 0.514 . . YES

XiaoGuo_18101 SignalP-4.0 SIGNAL 1 19 0.556 . . YES

XiaoGuo_32991 SignalP-4.0 SIGNAL 1 18 0.731 . . YES

XiaoGuo_37650 SignalP-4.0 SIGNAL 1 18 0.634 . . YES

XiaoGuo_37714 SignalP-4.0 SIGNAL 1 22 0.626 . . YES

XiaoGuo_37715 SignalP-4.0 SIGNAL 1 22 0.626 . . YES

XiaoGuo_38788 SignalP-4.0 SIGNAL 1 28 0.560 . . YES

XiaoGuo_39144 SignalP-4.0 SIGNAL 1 19 0.546 . . YES

XiaoGuo_39168 SignalP-4.0 SIGNAL 1 22 0.626 . . YES

XiaoGuo_42573 SignalP-4.0 SIGNAL 1 17 0.669 . . YES

XiaoGuo_42574 SignalP-4.0 SIGNAL 1 20 0.573 . . YES

XiaoGuo_42575 SignalP-4.0 SIGNAL 1 18 0.584 . . YES

XiaoGuo_42579 SignalP-4.0 SIGNAL 1 17 0.500 . . YES

XiaoGuo_45621 SignalP-4.0 SIGNAL 1 18 0.525 . . YES

XiaoGuo_56729 SignalP-4.0 SIGNAL 1 25 0.653 . . YES

XiaoGuo_59406 SignalP-4.0 SIGNAL 1 20 0.583 . . YES

XiaoGuo_63110 SignalP-4.0 SIGNAL 1 25 0.589 . . YES

XiaoGuo_68185 SignalP-4.0 SIGNAL 1 24 0.651 . . YES

XiaoGuo_68597 SignalP-4.0 SIGNAL 1 25 0.458 . . YES
