# Supplementary material for: Integration of Transcriptome, Proteome and Metabolism Data Reveals the Alkaloids Biosynthesis in Macleaya cordata and Macleaya microcarpa
Source: PLoS One. 2013 Jan 9;8(1):e53409. doi: 10.1371/journal.pone.0053409 (PMC3541140; doi:10.1371/journal.pone.0053409)
Supplement: File S5 — The transmembrane domains (TMD) results for protein identified by proteome. The identified proteins were conducted using the TMHMM 2.0 program based on transmembrane hidden Markov model (http://www.cbs.dtu.dk/services/TMHMM). (DOCX) [file pone.0053409.s015.docx]

Public

gi_122166028_sp_Q09G39.1_ATPB_PLAOC len=498 ExpAA=0.14 First60=0.00 PredHel=0 Topology=o

gi_37720941_gb_AAN32473.1_ len=481 ExpAA=0.11 First60=0.01 PredHel=0 Topology=o

gi_13236786_gb_AAK14698.1_ len=498 ExpAA=0.16 First60=0.03 PredHel=0 Topology=o

gi_147838606_emb_CAN69512.1_ len=560 ExpAA=0.03 First60=0.00 PredHel=0 Topology=o

gi_8134570_sp_Q42699.1_METE_CATRO len=765 ExpAA=0.01 First60=0.00 PredHel=0 Topology=o

gi_123325687_gb_ABM74468.1_ len=466 ExpAA=0.05 First60=0.00 PredHel=0 Topology=o

gi_225810599_gb_ACO34814.1_ len=757 ExpAA=0.04 First60=0.00 PredHel=0 Topology=o

gi_14718206_gb_AAK72841.1_ len=482 ExpAA=0.17 First60=0.04 PredHel=0 Topology=o

gi_61968201_gb_AAX57027.1_ len=479 ExpAA=0.15 First60=0.00 PredHel=0 Topology=o

gi_17939849_emb_CAC81058.1_ len=589 ExpAA=0.05 First60=0.00 PredHel=0 Topology=o

gi_9466236_gb_AAF76332.2_ len=375 ExpAA=0.46 First60=0.36 PredHel=0 Topology=o

gi_21389109_gb_AAM50489.1_ len=372 ExpAA=0.46 First60=0.32 PredHel=0 Topology=o

gi_464024_gb_AAA16968.1_ len=475 ExpAA=0.14 First60=0.00 PredHel=0 Topology=o

gi_33312161_gb_AAQ04019.1_ len=440 ExpAA=0.15 First60=0.00 PredHel=0 Topology=o

gi_4038532_emb_CAB02226.1_ len=455 ExpAA=0.18 First60=0.00 PredHel=0 Topology=o

gi_62861063_gb_AAY16643.1_ len=465 ExpAA=0.13 First60=0.00 PredHel=0 Topology=o

gi_7677038_gb_AAF66999.1_AF139877_1 len=449 ExpAA=0.25 First60=0.00 PredHel=0 Topology=o

gi_297828850_ref_XP_002882307.1_ len=765 ExpAA=0.09 First60=0.00 PredHel=0 Topology=o

gi_20257374_gb_AAM15869.1_ len=363 ExpAA=0.13 First60=0.01 PredHel=0 Topology=o

gi_1703107_sp_P53504.1_ACT1_SORBI len=377 ExpAA=1.01 First60=0.00 PredHel=0 Topology=o

gi_119952178_dbj_BAA96365.3_ len=332 ExpAA=0.80 First60=0.00 PredHel=0 Topology=o

gi_257344495_emb_CBC02905.1_ len=652 ExpAA=0.03 First60=0.01 PredHel=0 Topology=o

gi_257344397_emb_CBC02856.1_ len=651 ExpAA=0.02 First60=0.00 PredHel=0 Topology=o

gi_312281705_dbj_BAJ33718.1_ len=437 ExpAA=0.30 First60=0.22 PredHel=0 Topology=o

gi_125555777_gb_EAZ01383.1_ len=527 ExpAA=0.00 First60=0.00 PredHel=0 Topology=o

gi_14594788_emb_CAC43404.1_ len=443 ExpAA=0.08 First60=0.00 PredHel=0 Topology=o

gi_255566442_ref_XP_002524206.1_ len=473 ExpAA=0.22 First60=0.14 PredHel=0 Topology=o

gi_552890_gb_AAA84647.1_ len=443 ExpAA=0.12 First60=0.00 PredHel=0 Topology=o

gi_17224671_gb_AAL37070.1_ len=346 ExpAA=0.46 First60=0.31 PredHel=0 Topology=o

gi_37722344_gb_AAN34911.1_ len=460 ExpAA=0.14 First60=0.00 PredHel=0 Topology=o

gi_1050426_emb_CAA58603.1_ len=469 ExpAA=0.05 First60=0.00 PredHel=0 Topology=o

gi_15187136_gb_AAK91305.1_ len=327 ExpAA=1.30 First60=1.09 PredHel=0 Topology=o

gi_32332099_gb_AAO73531.1_ len=372 ExpAA=0.82 First60=0.57 PredHel=0 Topology=o

gi_114527_sp_P06450.1_ATPA_SPIOL len=507 ExpAA=0.03 First60=0.00 PredHel=0 Topology=o

gi_170516170_gb_ACB15095.1_ len=454 ExpAA=0.07 First60=0.00 PredHel=0 Topology=o

gi_4512327_dbj_BAA75231.1_ len=155 ExpAA=6.87 First60=0.00 PredHel=0 Topology=o

gi_300262872_gb_EFJ47076.1_ len=409 ExpAA=0.01 First60=0.00 PredHel=0 Topology=o

gi_222853777_gb_EEE91324.1_ len=354 ExpAA=15.64 First60=14.94 PredHel=0 Topology=o

gi_301176645_gb_ADK66339.1_ len=1028 ExpAA=19.86 First60=19.85 PredHel=1 Topology=i7-29o

gi_121309841_dbj_BAF44222.1_ len=307 ExpAA=19.93 First60=14.39 PredHel=0 Topology=o

gi_3913786_sp_Q42434.1_BIP_SPIOL len=668 ExpAA=18.64 First60=18.61 PredHel=1 Topology=i9-28o

gi_110740085_dbj_BAF01944.1_ len=393 ExpAA=0.00 First60=0.00 PredHel=0 Topology=o

gi_302809252_ref_XP_002986319.1_ len=517 ExpAA=0.30 First60=0.00 PredHel=0 Topology=o

gi_74053562_sp_Q40665.2_TBB3_ORYSJ len=446 ExpAA=0.04 First60=0.00 PredHel=0 Topology=o

gi_37574478_gb_AAM98365.1_ len=264 ExpAA=0.93 First60=0.79 PredHel=0 Topology=o

gi_61659100_gb_AAX49653.1_ len=166 ExpAA=0.18 First60=0.00 PredHel=0 Topology=o

gi_21105258_gb_AAM34575.1_ len=245 ExpAA=0.25 First60=0.00 PredHel=0 Topology=o

gi_46325913_gb_AAS88137.1_ len=261 ExpAA=0.66 First60=0.00 PredHel=0 Topology=o

gi_223533169_gb_EEF34926.1_ len=373 ExpAA=0.08 First60=0.06 PredHel=0 Topology=o

gi_188526499_gb_ACD62225.1_ len=277 ExpAA=0.44 First60=0.00 PredHel=0 Topology=o

gi_50284513_dbj_BAD29709.1_ len=396 ExpAA=0.00 First60=0.00 PredHel=0 Topology=o

gi_262410515_gb_ACY66804.1_ len=254 ExpAA=0.16 First60=0.00 PredHel=0 Topology=o

gi_21311559_gb_AAM46780.1_AF467803_1 len=396 ExpAA=0.09 First60=0.00 PredHel=0 Topology=o

gi_222836150_gb_EEE74571.1_ len=496 ExpAA=0.44 First60=0.00 PredHel=0 Topology=o

gi_194703312_gb_ACF85740.1_ len=265 ExpAA=0.00 First60=0.00 PredHel=0 Topology=o

gi_188037513_gb_ACD46367.1_ len=202 ExpAA=0.12 First60=0.00 PredHel=0 Topology=o

gi_308742593_gb_ADO33444.1_ len=513 ExpAA=142.68 First60=0.00 PredHel=6 Topology=i89-111o149-171i200-222o273-290i311-333o466-485i

gi_224074105_ref_XP_002304255.1_ len=481 ExpAA=0.08 First60=0.00 PredHel=0 Topology=o

gi_223542730_gb_EEF44267.1_ len=615 ExpAA=3.26 First60=0.00 PredHel=0 Topology=o

gi_224137704_ref_XP_002322623.1_ len=1491 ExpAA=0.21 First60=0.00 PredHel=0 Topology=o

gi_1229138_gb_AAB07749.1_ len=161 ExpAA=0.00 First60=0.00 PredHel=0 Topology=o

gi_22655284_gb_AAM98232.1_ len=447 ExpAA=0.05 First60=0.00 PredHel=0 Topology=o

gi_37959586_gb_AAP42906.1_ len=423 ExpAA=0.33 First60=0.00 PredHel=0 Topology=o

gi_224056853_ref_XP_002299056.1_ len=357 ExpAA=0.03 First60=0.00 PredHel=0 Topology=o

gi_219797738_emb_CAW55669.1_ len=967 ExpAA=0.05 First60=0.00 PredHel=0 Topology=o

gi_223529618_gb_EEF31566.1_ len=249 ExpAA=0.17 First60=0.00 PredHel=0 Topology=o

gi_223546334_gb_EEF47836.1_ len=264 ExpAA=0.08 First60=0.00 PredHel=0 Topology=o

gi_59938793_gb_AAX12161.1_ len=97 ExpAA=0.13 First60=0.08 PredHel=0 Topology=o

gi_257696350_emb_CBC70129.1_ len=250 ExpAA=0.00 First60=0.00 PredHel=0 Topology=o

gi_100526_pir__S24992 len=187 ExpAA=47.95 First60=23.97 PredHel=2 Topology=o31-53i107-129o

gi_171259300_ref_YP_654212.2_ len=507 ExpAA=0.03 First60=0.00 PredHel=0 Topology=o

gi_223533733_gb_EEF35467.1_ len=1632 ExpAA=0.14 First60=0.00 PredHel=0 Topology=o

gi_187453117_emb_CAP72489.1_ len=360 ExpAA=9.07 First60=9.05 PredHel=0 Topology=o

gi_114804260_ref_YP_762256.1_ len=353 ExpAA=132.80 First60=23.05 PredHel=6 Topology=i28-50o109-128i141-163o168-185i197-219o270-292i

gi_162689394_gb_EDQ75766.1_ len=504 ExpAA=0.47 First60=0.32 PredHel=0 Topology=o

gi_297804932_ref_XP_002870350.1_ len=485 ExpAA=0.02 First60=0.00 PredHel=0 Topology=o

gi_225446775_ref_XP_002283048.1_ len=259 ExpAA=0.07 First60=0.04 PredHel=0 Topology=o

gi_255540075_ref_XP_002511102.1_ len=924 ExpAA=0.03 First60=0.00 PredHel=0 Topology=o

gi_1895084_gb_AAB49896.1_ len=364 ExpAA=0.02 First60=0.00 PredHel=0 Topology=o

gi_147801802_emb_CAN74538.1_ len=587 ExpAA=0.01 First60=0.00 PredHel=0 Topology=o

gi_15809970_gb_AAL06912.1_ len=444 ExpAA=9.94 First60=0.00 PredHel=0 Topology=o

gi_13873334_dbj_BAB44155.1_ len=386 ExpAA=0.12 First60=0.00 PredHel=0 Topology=o

gi_222834384_gb_EEE72861.1_ len=472 ExpAA=0.06 First60=0.00 PredHel=0 Topology=o

gi_227467174_emb_CAY37595.1_ len=744 ExpAA=1.37 First60=0.00 PredHel=0 Topology=o

gi_115444503_ref_NP_001046031.1_ len=411 ExpAA=0.00 First60=0.00 PredHel=0 Topology=o

gi_116831754_gb_ABK28851.1_ len=451 ExpAA=1.83 First60=0.00 PredHel=0 Topology=o

gi_146744194_gb_ABQ43266.1_ len=133 ExpAA=0.08 First60=0.07 PredHel=0 Topology=o

gi_147770035_emb_CAN74334.1_ len=372 ExpAA=1.11 First60=0.04 PredHel=0 Topology=o

gi_241941185_gb_EES14330.1_ len=788 ExpAA=0.00 First60=0.00 PredHel=0 Topology=o

gi_147795191_emb_CAN69459.1_ len=453 ExpAA=0.03 First60=0.00 PredHel=0 Topology=o

gi_225466944_ref_XP_002263330.1_ len=142 ExpAA=14.05 First60=14.05 PredHel=0 Topology=o

gi_108710847_gb_ABF98642.1_ len=451 ExpAA=1.90 First60=0.00 PredHel=0 Topology=o

gi_2982322_gb_AAC32146.1_ len=204 ExpAA=0.13 First60=0.11 PredHel=0 Topology=o

gi_1346735_sp_P35493.2_PMGI_RICCO len=556 ExpAA=0.00 First60=0.00 PredHel=0 Topology=o

gi_302399079_gb_ADL36834.1_ len=527 ExpAA=1.38 First60=1.37 PredHel=0 Topology=o

gi_212722842_ref_NP_001131288.1_ len=402 ExpAA=0.18 First60=0.00 PredHel=0 Topology=o

gi_283146497_gb_ADB13189.1_ len=88 ExpAA=0.00 First60=0.00 PredHel=0 Topology=o

gi_226320258_gb_ACO48250.1_ len=966 ExpAA=0.01 First60=0.00 PredHel=0 Topology=o

gi_255638991_gb_ACU19796.1_ len=315 ExpAA=0.16 First60=0.00 PredHel=0 Topology=o

gi_118488483_gb_ABK96055.1_ len=429 ExpAA=3.47 First60=0.50 PredHel=0 Topology=o

gi_105873020_gb_ABF74733.1_ len=181 ExpAA=0.04 First60=0.04 PredHel=0 Topology=o

gi_241919731_gb_EER92875.1_ len=712 ExpAA=0.00 First60=0.00 PredHel=0 Topology=o

gi_106879575_emb_CAJ38371.1_ len=212 ExpAA=22.20 First60=22.20 PredHel=1 Topology=o15-37i

gi_225456481_ref_XP_002280858.1_ len=1009 ExpAA=1.86 First60=1.80 PredHel=0 Topology=o

gi_116780962_gb_ABK21901.1_ len=197 ExpAA=0.00 First60=0.00 PredHel=0 Topology=o

gi_110283023_sp_Q56XG6.2_RH15_ARATH len=427 ExpAA=0.00 First60=0.00 PredHel=0 Topology=o

gi_162673928_gb_EDQ60444.1_ len=358 ExpAA=0.00 First60=0.00 PredHel=0 Topology=o

gi_219908399_emb_CAX03474.1_ len=332 ExpAA=4.23 First60=4.23 PredHel=0 Topology=o

gi_224069046_ref_XP_002326261.1_ len=560 ExpAA=0.01 First60=0.00 PredHel=0 Topology=o

gi_113649478_dbj_BAF29990.1_ len=220 ExpAA=0.11 First60=0.00 PredHel=0 Topology=o

gi_147790711_emb_CAN76515.1_ len=130 ExpAA=0.05 First60=0.02 PredHel=0 Topology=o

gi_297853126_ref_XP_002894444.1_ len=668 ExpAA=4.38 First60=4.36 PredHel=0 Topology=o

gi_147782460_emb_CAN61919.1_ len=802 ExpAA=0.00 First60=0.00 PredHel=0 Topology=o

gi_197916899_gb_ABS42984.2_ len=249 ExpAA=0.08 First60=0.00 PredHel=0 Topology=o

gi_118487642_gb_ABK95646.1_ len=290 ExpAA=16.05 First60=0.02 PredHel=0 Topology=o

gi_298205241_emb_CBI17300.3_ len=244 ExpAA=0.02 First60=0.00 PredHel=0 Topology=o

gi_116783314_gb_ABK22887.1_ len=199 ExpAA=0.03 First60=0.00 PredHel=0 Topology=o

gi_219726082_emb_CAW50713.1_ len=389 ExpAA=0.92 First60=0.69 PredHel=0 Topology=o

gi_2253221_gb_AAB62882.1_ len=336 ExpAA=2.47 First60=0.00 PredHel=0 Topology=o

gi_17368377_sp_P94026.1_RBCMT_TOBAC len=491 ExpAA=0.02 First60=0.00 PredHel=0 Topology=o

gi_113649389_dbj_BAF29901.1_ len=811 ExpAA=0.00 First60=0.00 PredHel=0 Topology=o

gi_147805559_emb_CAN65023.1_ len=386 ExpAA=0.10 First60=0.00 PredHel=0 Topology=o

gi_147854971_emb_CAN80262.1_ len=358 ExpAA=0.00 First60=0.00 PredHel=0 Topology=o

gi_159147191_gb_ABW90990.1_ len=287 ExpAA=16.29 First60=0.00 PredHel=0 Topology=o

gi_115479163_ref_NP_001063175.1_ len=527 ExpAA=0.67 First60=0.00 PredHel=0 Topology=o

gi_149941230_emb_CAO02547.1_ len=201 ExpAA=0.04 First60=0.00 PredHel=0 Topology=o

gi_215692861_dbj_BAG88281.1_ len=153 ExpAA=0.00 First60=0.00 PredHel=0 Topology=o

gi_219908431_emb_CAX03490.1_ len=354 ExpAA=17.82 First60=17.80 PredHel=1 Topology=i5-27o

gi_222842607_gb_EEE80154.1_ len=327 ExpAA=6.56 First60=0.00 PredHel=0 Topology=o

gi_227478251_emb_CAY38949.1_ len=354 ExpAA=0.00 First60=0.00 PredHel=0 Topology=o

gi_219982817_emb_CAW93873.1_ len=1152 ExpAA=5.85 First60=4.79 PredHel=0 Topology=o

gi_283135908_gb_ADB11345.1_ len=212 ExpAA=0.00 First60=0.00 PredHel=0 Topology=o

gi_118481385_gb_ABK92635.1_ len=219 ExpAA=0.00 First60=0.00 PredHel=0 Topology=o

gi_296528874_emb_CBM37989.1_ len=888 ExpAA=0.00 First60=0.00 PredHel=0 Topology=o

gi_115471065_ref_NP_001059131.1_ len=889 ExpAA=0.06 First60=0.00 PredHel=0 Topology=o

gi_147855970_emb_CAN80740.1_ len=251 ExpAA=0.16 First60=0.07 PredHel=0 Topology=o

gi_125600834_gb_EAZ40410.1_ len=263 ExpAA=3.38 First60=0.35 PredHel=0 Topology=o

gi_108860772_sp_Q1S9I9.3_H2B1_MEDTR len=148 ExpAA=0.00 First60=0.00 PredHel=0 Topology=o

gi_17976705_emb_CAC80839.2_ len=571 ExpAA=0.08 First60=0.02 PredHel=0 Topology=o

gi_118481719_gb_ABK92799.1_ len=134 ExpAA=0.00 First60=0.00 PredHel=0 Topology=o

gi_192910862_gb_ACF06539.1_ len=146 ExpAA=0.00 First60=0.00 PredHel=0 Topology=o

gi_284433780_gb_ADB85096.1_ len=234 ExpAA=0.02 First60=0.01 PredHel=0 Topology=o

gi_2196542_gb_AAB61213.1_ len=197 ExpAA=0.30 First60=0.28 PredHel=0 Topology=o

gi_113722137_gb_AAW51923.2_ len=723 ExpAA=0.70 First60=0.01 PredHel=0 Topology=o

gi_116787002_gb_ABK24337.1_ len=394 ExpAA=0.04 First60=0.00 PredHel=0 Topology=o

gi_311698157_gb_ADQ00373.1_ len=261 ExpAA=0.02 First60=0.00 PredHel=0 Topology=o

gi_222864249_gb_EEF01380.1_ len=1377 ExpAA=0.01 First60=0.00 PredHel=0 Topology=o

gi_222844475_gb_EEE82022.1_ len=321 ExpAA=1.73 First60=1.70 PredHel=0 Topology=o

gi_2494073_sp_Q40024.1_BADH_HORVU len=505 ExpAA=0.08 First60=0.00 PredHel=0 Topology=o

gi_113564475_dbj_BAF14818.1_ len=442 ExpAA=12.76 First60=0.16 PredHel=0 Topology=o

gi_122063219_sp_P04464.3_CALM_WHEAT len=149 ExpAA=0.00 First60=0.00 PredHel=0 Topology=o

gi_1079736_gb_AAA82069.1_ len=178 ExpAA=0.25 First60=0.25 PredHel=0 Topology=o

gi_115779_sp_P12471.1_CB21_SOYBN len=245 ExpAA=1.61 First60=0.00 PredHel=0 Topology=o

gi_115477084_ref_NP_001062138.1_ len=661 ExpAA=219.21 First60=12.60 PredHel=9 Topology=o295-317i369-391o396-418i431-453o468-490i518-540o550-572i585-607o629-651i

gi_147866290_emb_CAN82036.1_ len=222 ExpAA=0.49 First60=0.00 PredHel=0 Topology=o

gi_242071867_ref_XP_002451210.1_ len=271 ExpAA=2.70 First60=1.96 PredHel=0 Topology=o

gi_115529229_dbj_BAF34340.1_ len=163 ExpAA=0.00 First60=0.00 PredHel=0 Topology=o

gi_113648813_dbj_BAF29325.1_ len=320 ExpAA=0.06 First60=0.01 PredHel=0 Topology=o

gi_222850891_gb_EEE88438.1_ len=513 ExpAA=3.63 First60=2.66 PredHel=0 Topology=o

gi_2792520_gb_AAB97087.1_ len=466 ExpAA=0.06 First60=0.00 PredHel=0 Topology=o

gi_1223922_gb_AAA92063.1_ len=362 ExpAA=6.74 First60=6.74 PredHel=0 Topology=o

gi_255584961_ref_XP_002533192.1_ len=377 ExpAA=0.63 First60=0.24 PredHel=0 Topology=o

gi_148537202_dbj_BAF63492.1_ len=21 ExpAA=0.00 First60=0.00 PredHel=0 Topology=o

gi_144576895_gb_ABO94962.1_ len=1056 ExpAA=0.08 First60=0.00 PredHel=0 Topology=o

gi_1170508_sp_P41381.1_IF4A8_TOBAC len=413 ExpAA=0.01 First60=0.00 PredHel=0 Topology=o

gi_148283923_gb_ABQ57397.1_ len=153 ExpAA=50.57 First60=18.82 PredHel=2 Topology=i44-66o76-98i

gi_296514470_emb_CBM40093.1_ len=271 ExpAA=1.31 First60=0.00 PredHel=0 Topology=o

gi_162669988_gb_EDQ56565.1_ len=152 ExpAA=0.00 First60=0.00 PredHel=0 Topology=o

gi_224053655_ref_XP_002297914.1_ len=209 ExpAA=0.04 First60=0.00 PredHel=0 Topology=o

gi_225437455_ref_XP_002273033.1_ len=494 ExpAA=0.03 First60=0.03 PredHel=0 Topology=o

gi_116792150_gb_ABK26251.1_ len=239 ExpAA=1.28 First60=0.12 PredHel=0 Topology=o

gi_113533304_dbj_BAF05687.1_ len=367 ExpAA=0.02 First60=0.00 PredHel=0 Topology=o

gi_302786848_ref_XP_002975195.1_ len=460 ExpAA=0.03 First60=0.00 PredHel=0 Topology=o

gi_60593322_gb_AAX28927.1_ len=197 ExpAA=18.22 First60=18.10 PredHel=1 Topology=i7-26o

gi_1449179_dbj_BAA13101.1_ len=739 ExpAA=0.04 First60=0.02 PredHel=0 Topology=o

gi_148906755_gb_ABR16524.1_ len=509 ExpAA=0.06 First60=0.02 PredHel=0 Topology=o

gi_194707148_gb_ACF87658.1_ len=360 ExpAA=5.65 First60=3.63 PredHel=0 Topology=o

gi_115187479_gb_ABI84255.1_ len=168 ExpAA=0.00 First60=0.00 PredHel=0 Topology=o

gi_21536557_gb_AAM60889.1_ len=347 ExpAA=0.13 First60=0.00 PredHel=0 Topology=o

gi_13936812_gb_AAK49947.1_AF335551_1 len=326 ExpAA=0.00 First60=0.00 PredHel=0 Topology=o

gi_222845136_gb_EEE82683.1_ len=690 ExpAA=0.05 First60=0.00 PredHel=0 Topology=o

gi_108773129_ref_YP_635638.1_ len=734 ExpAA=247.39 First60=10.01 PredHel=9 Topology=i135-157o177-199i332-354o374-396i417-439o517-539i575-594o645-667i707-729o

gi_147856546_emb_CAN82488.1_ len=466 ExpAA=0.31 First60=0.00 PredHel=0 Topology=o

gi_222850910_gb_EEE88457.1_ len=609 ExpAA=0.01 First60=0.00 PredHel=0 Topology=o

gi_14597780_emb_CAC43713.1_ len=305 ExpAA=0.03 First60=0.02 PredHel=0 Topology=o

gi_219725680_emb_CAW61265.1_ len=311 ExpAA=0.01 First60=0.00 PredHel=0 Topology=o

gi_255559645_ref_XP_002520842.1_ len=389 ExpAA=0.52 First60=0.00 PredHel=0 Topology=o

gi_118482340_gb_ABK93094.1_ len=358 ExpAA=17.53 First60=17.51 PredHel=1 Topology=i7-25o

gi_222871974_gb_EEF09105.1_ len=172 ExpAA=0.01 First60=0.00 PredHel=0 Topology=o

gi_255559665_ref_XP_002520852.1_ len=360 ExpAA=0.05 First60=0.04 PredHel=0 Topology=o

gi_224134346_ref_XP_002327815.1_ len=398 ExpAA=0.00 First60=0.00 PredHel=0 Topology=o

gi_118481419_gb_ABK92652.1_ len=150 ExpAA=0.01 First60=0.01 PredHel=0 Topology=i

gi_223538100_gb_EEF39711.1_ len=112 ExpAA=0.00 First60=0.00 PredHel=0 Topology=o

gi_114216040_gb_ABI54631.1_ len=236 ExpAA=0.02 First60=0.00 PredHel=0 Topology=o

gi_192913046_gb_ACF06631.1_ len=310 ExpAA=5.37 First60=0.14 PredHel=0 Topology=o

gi_118482644_gb_ABK93241.1_ len=297 ExpAA=1.68 First60=0.00 PredHel=0 Topology=o

gi_118487464_gb_ABK95559.1_ len=539 ExpAA=0.00 First60=0.00 PredHel=0 Topology=o

gi_113595155_dbj_BAF19029.1_ len=368 ExpAA=38.64 First60=0.65 PredHel=0 Topology=o

gi_162461510_ref_NP_001105330.1_ len=167 ExpAA=0.00 First60=0.00 PredHel=0 Topology=o

gi_225464186_ref_XP_002263334.1_ len=445 ExpAA=0.00 First60=0.00 PredHel=0 Topology=o

gi_255290444_emb_CBA12822.1_ len=1010 ExpAA=0.22 First60=0.00 PredHel=0 Topology=o

gi_118486315_gb_ABK94999.1_ len=429 ExpAA=0.09 First60=0.00 PredHel=0 Topology=o

gi_224115794_ref_XP_002317126.1_ len=512 ExpAA=19.21 First60=19.14 PredHel=1 Topology=i7-24o

gi_161375756_gb_ABX71220.1_ len=230 ExpAA=18.99 First60=18.97 PredHel=1 Topology=i7-29o

gi_255626267_gb_ACU13478.1_ len=246 ExpAA=0.57 First60=0.35 PredHel=0 Topology=o

gi_195619012_gb_ACG31336.1_ len=157 ExpAA=0.00 First60=0.00 PredHel=0 Topology=o

gi_10799832_emb_CAC12883.1_ len=181 ExpAA=0.01 First60=0.00 PredHel=0 Topology=o

gi_296511727_emb_CBM39005.1_ len=435 ExpAA=0.17 First60=0.06 PredHel=0 Topology=o

gi_116317926_emb_CAH65949.1_ len=425 ExpAA=2.41 First60=0.00 PredHel=0 Topology=o

gi_12644436_sp_Q43743.2_MDHG1_BRANA len=358 ExpAA=2.96 First60=2.17 PredHel=0 Topology=o

gi_148906385_gb_ABR16347.1_ len=393 ExpAA=1.28 First60=0.00 PredHel=0 Topology=o

gi_222862686_gb_EEF00193.1_ len=403 ExpAA=0.83 First60=0.00 PredHel=0 Topology=o

gi_118486265_gb_ABK94974.1_ len=531 ExpAA=1.12 First60=0.00 PredHel=0 Topology=o

gi_225455902_ref_XP_002276180.1_ len=587 ExpAA=0.21 First60=0.00 PredHel=0 Topology=o

gi_20466173_gb_AAM20404.1_ len=386 ExpAA=0.13 First60=0.00 PredHel=0 Topology=o

gi_113374057_dbj_BAF03589.1_ len=964 ExpAA=208.64 First60=0.00 PredHel=9 Topology=i65-87o97-116i244-266o281-303i649-671o713-735i769-786o801-823i830-852o

gi_225431033_ref_XP_002279911.1_ len=357 ExpAA=0.79 First60=0.02 PredHel=0 Topology=o

gi_193290724_gb_ACF17670.1_ len=408 ExpAA=0.11 First60=0.00 PredHel=0 Topology=o

gi_13430788_gb_AAK26016.1_AF360306_1 len=389 ExpAA=0.28 First60=0.00 PredHel=0 Topology=o

gi_118489486_gb_ABK96545.1_ len=414 ExpAA=9.48 First60=0.00 PredHel=0 Topology=o

gi_10716959_gb_AAG21982.1_ len=100 ExpAA=0.47 First60=0.47 PredHel=0 Topology=o

gi_255551408_ref_XP_002516750.1_ len=535 ExpAA=0.02 First60=0.00 PredHel=0 Topology=o

gi_310689575_gb_ADP03299.1_ len=77 ExpAA=0.23 First60=0.07 PredHel=0 Topology=o

gi_1008445_emb_CAA61945.1_ len=140 ExpAA=0.08 First60=0.00 PredHel=0 Topology=o

gi_168603_gb_AAA33505.1_ len=248 ExpAA=0.03 First60=0.00 PredHel=0 Topology=o

gi_217073648_gb_ACJ85184.1_ len=394 ExpAA=0.05 First60=0.02 PredHel=0 Topology=o

gi_118487306_gb_ABK95481.1_ len=363 ExpAA=0.03 First60=0.00 PredHel=0 Topology=o

gi_118482366_gb_ABK93106.1_ len=539 ExpAA=0.00 First60=0.00 PredHel=0 Topology=o

gi_11131774_sp_Q40406.1_CRTI_NARPS len=570 ExpAA=0.03 First60=0.00 PredHel=0 Topology=o

gi_118483154_gb_ABK93483.1_ len=535 ExpAA=0.00 First60=0.00 PredHel=0 Topology=o

gi_222872806_gb_EEF09937.1_ len=1215 ExpAA=0.11 First60=0.00 PredHel=0 Topology=o

gi_297734385_emb_CBI15632.3_ len=485 ExpAA=0.39 First60=0.00 PredHel=0 Topology=o

gi_20148479_gb_AAM10130.1_ len=231 ExpAA=0.02 First60=0.00 PredHel=0 Topology=o

gi_2465012_emb_CAA04769.1_ len=188 ExpAA=2.44 First60=2.38 PredHel=0 Topology=o

gi_124359133_gb_ABD32492.2_ len=65 ExpAA=0.00 First60=0.00 PredHel=0 Topology=i

gi_1346109_sp_P49027.1_GBLPA_ORYSJ len=334 ExpAA=0.03 First60=0.02 PredHel=0 Topology=o

gi_10946427_gb_AAG24908.1_AF305075_1 len=264 ExpAA=0.00 First60=0.00 PredHel=0 Topology=o

gi_12585325_sp_Q9SMM0.1_PGMP_BRANA len=629 ExpAA=0.04 First60=0.00 PredHel=0 Topology=o

gi_126633570_emb_CAM55750.1_ len=362 ExpAA=20.79 First60=20.74 PredHel=1 Topology=i9-31o

gi_223543915_gb_EEF45441.1_ len=872 ExpAA=0.00 First60=0.00 PredHel=0 Topology=o

gi_118483826_gb_ABK93805.1_ len=212 ExpAA=0.00 First60=0.00 PredHel=0 Topology=o

gi_147820654_emb_CAN65362.1_ len=378 ExpAA=1.16 First60=0.00 PredHel=0 Topology=o

gi_307110069_gb_EFN58306.1_ len=369 ExpAA=0.03 First60=0.00 PredHel=0 Topology=o

gi_118486938_gb_ABK95302.1_ len=498 ExpAA=0.28 First60=0.21 PredHel=0 Topology=o

gi_147795432_emb_CAN77106.1_ len=600 ExpAA=205.17 First60=18.52 PredHel=9 Topology=i7-24o216-238i285-307o317-339i352-374o389-411i490-512o527-549i562-584o

gi_114054410_gb_ABI49804.1_ len=202 ExpAA=8.56 First60=0.08 PredHel=0 Topology=o

gi_125540786_gb_EAY87181.1_ len=166 ExpAA=0.03 First60=0.03 PredHel=0 Topology=o

gi_110738557_dbj_BAF01204.1_ len=250 ExpAA=142.72 First60=28.18 PredHel=6 Topology=i21-43o58-80i101-123o133-155i167-189o214-236i

gi_11022925_gb_AAG26202.1_ len=69 ExpAA=21.91 First60=21.91 PredHel=1 Topology=i7-29o

gi_148908772_gb_ABR17492.1_ len=482 ExpAA=0.80 First60=0.79 PredHel=0 Topology=o

gi_213521174_gb_ACJ50528.1_ len=399 ExpAA=0.00 First60=0.00 PredHel=0 Topology=o

gi_225444889_ref_XP_002281573.1_ len=368 ExpAA=0.00 First60=0.00 PredHel=0 Topology=o

gi_255571417_ref_XP_002526656.1_ len=872 ExpAA=0.03 First60=0.00 PredHel=0 Topology=o

gi_10177452_dbj_BAB10843.1_ len=286 ExpAA=0.01 First60=0.00 PredHel=0 Topology=o

gi_92882356_gb_ABE86687.1_ len=576 ExpAA=0.00 First60=0.00 PredHel=0 Topology=o

gi_11066213_gb_AAG28503.1_AF196966_1 len=498 ExpAA=20.53 First60=19.76 PredHel=1 Topology=i7-24o

gi_108862591_gb_ABA97701.2_ len=542 ExpAA=0.49 First60=0.00 PredHel=0 Topology=o

gi_85543776_gb_ABC71784.1_ len=164 ExpAA=72.61 First60=25.75 PredHel=3 Topology=o30-52i73-95o118-137i

gi_195648210_gb_ACG43573.1_ len=743 ExpAA=0.00 First60=0.00 PredHel=0 Topology=o

gi_2645999_gb_AAB87573.1_ len=266 ExpAA=1.69 First60=0.07 PredHel=0 Topology=o

gi_1009232_gb_AAA79032.1_ len=515 ExpAA=0.02 First60=0.00 PredHel=0 Topology=o

gi_15225009_ref_NP_181435.1_ len=236 ExpAA=0.61 First60=0.00 PredHel=0 Topology=o

gi_22795909_emb_CAD23928.1_ len=132 ExpAA=0.09 First60=0.08 PredHel=0 Topology=o

gi_11131949_sp_Q9ZPP1.1_CALR_BERST len=416 ExpAA=0.00 First60=0.00 PredHel=0 Topology=o

gi_222843894_gb_EEE81441.1_ len=389 ExpAA=0.22 First60=0.00 PredHel=0 Topology=o

gi_302143338_emb_CBI21899.3_ len=890 ExpAA=12.80 First60=0.00 PredHel=0 Topology=o

gi_255645227_gb_ACU23111.1_ len=358 ExpAA=0.01 First60=0.01 PredHel=0 Topology=o

gi_162677964_gb_EDQ64428.1_ len=599 ExpAA=0.01 First60=0.00 PredHel=0 Topology=o

gi_147224163_emb_CAN08801.1_ len=455 ExpAA=0.11 First60=0.08 PredHel=0 Topology=o

gi_113535904_dbj_BAF08287.1_ len=539 ExpAA=0.89 First60=0.01 PredHel=0 Topology=o

gi_115461208_ref_NP_001054204.1_ len=266 ExpAA=19.13 First60=18.75 PredHel=1 Topology=i7-25o

gi_1419088_emb_CAA95999.1_ len=416 ExpAA=18.62 First60=18.61 PredHel=1 Topology=i12-31o

gi_147770754_emb_CAN62470.1_ len=234 ExpAA=0.23 First60=0.21 PredHel=0 Topology=o

gi_15810421_gb_AAL07098.1_ len=375 ExpAA=0.04 First60=0.00 PredHel=0 Topology=o

gi_115478593_ref_NP_001062890.1_ len=319 ExpAA=0.11 First60=0.11 PredHel=0 Topology=o

gi_147797328_emb_CAN67085.1_ len=243 ExpAA=0.02 First60=0.00 PredHel=0 Topology=o

gi_1184989_gb_AAA87886.1_ len=140 ExpAA=0.01 First60=0.01 PredHel=0 Topology=o

gi_116791301_gb_ABK25927.1_ len=187 ExpAA=0.00 First60=0.00 PredHel=0 Topology=o

gi_1346180_sp_P49310.1_GRP1_SINAL len=166 ExpAA=0.00 First60=0.00 PredHel=0 Topology=o

gi_118488569_gb_ABK96097.1_ len=249 ExpAA=0.21 First60=0.00 PredHel=0 Topology=o

gi_110224748_emb_CAL07974.1_ len=167 ExpAA=13.29 First60=0.19 PredHel=0 Topology=o

gi_225464297_ref_XP_002265101.1_ len=761 ExpAA=0.21 First60=0.00 PredHel=0 Topology=o

gi_223551374_gb_EEF52860.1_ len=131 ExpAA=0.00 First60=0.00 PredHel=0 Topology=o

gi_186489067_ref_NP_001117440.1_ len=335 ExpAA=0.01 First60=0.00 PredHel=0 Topology=o

gi_116791660_gb_ABK26061.1_ len=106 ExpAA=1.10 First60=0.25 PredHel=0 Topology=o

gi_11267_emb_CAA40875.1_ len=260 ExpAA=44.57 First60=19.74 PredHel=2 Topology=o40-62i83-105o

gi_217074084_gb_ACJ85402.1_ len=423 ExpAA=0.00 First60=0.00 PredHel=0 Topology=o

gi_108708059_gb_ABF95854.1_ len=642 ExpAA=0.36 First60=0.00 PredHel=0 Topology=o

gi_11263719_pir__JC7138 len=437 ExpAA=20.92 First60=20.90 PredHel=1 Topology=i7-29o

gi_255647108_gb_ACU24022.1_ len=378 ExpAA=1.63 First60=1.00 PredHel=0 Topology=o

gi_5442414_gb_AAD43336.1_ len=250 ExpAA=0.01 First60=0.00 PredHel=0 Topology=o

gi_270383724_dbj_BAI53120.1_ len=179 ExpAA=0.03 First60=0.03 PredHel=0 Topology=o

gi_125525217_gb_EAY73331.1_ len=526 ExpAA=0.01 First60=0.00 PredHel=0 Topology=o

gi_110832726_sp_Q5Z8Z7.2_2ABA_ORYSJ len=581 ExpAA=0.37 First60=0.00 PredHel=0 Topology=o

gi_106879633_emb_CAJ38400.1_ len=186 ExpAA=0.01 First60=0.00 PredHel=0 Topology=o

gi_118483625_gb_ABK93707.1_ len=324 ExpAA=0.00 First60=0.00 PredHel=0 Topology=o

gi_255635880_gb_ACU18287.1_ len=304 ExpAA=11.13 First60=0.37 PredHel=0 Topology=o

gi_113612079_dbj_BAF22457.1_ len=224 ExpAA=0.01 First60=0.00 PredHel=0 Topology=o

gi_223529250_gb_EEF31222.1_ len=212 ExpAA=0.00 First60=0.00 PredHel=0 Topology=o

gi_110741229_dbj_BAF02165.1_ len=727 ExpAA=2.24 First60=0.00 PredHel=0 Topology=o

gi_223545125_gb_EEF46635.1_ len=261 ExpAA=0.03 First60=0.00 PredHel=0 Topology=o

gi_10177019_dbj_BAB10257.1_ len=244 ExpAA=0.00 First60=0.00 PredHel=0 Topology=o

gi_116781065_gb_ABK21951.1_ len=234 ExpAA=0.06 First60=0.06 PredHel=0 Topology=o

gi_17402589_dbj_BAB78715.1_ len=843 ExpAA=0.44 First60=0.27 PredHel=0 Topology=o

gi_168047145_ref_XP_001776032.1_ len=392 ExpAA=0.00 First60=0.00 PredHel=0 Topology=o

gi_118486589_gb_ABK95133.1_ len=201 ExpAA=0.06 First60=0.06 PredHel=0 Topology=o

gi_194693774_gb_ACF80971.1_ len=133 ExpAA=0.03 First60=0.00 PredHel=0 Topology=o

gi_158998780_gb_ABW86978.1_ len=505 ExpAA=21.32 First60=21.25 PredHel=1 Topology=i5-27o

gi_222861857_gb_EEE99399.1_ len=487 ExpAA=0.03 First60=0.00 PredHel=0 Topology=o

gi_118485281_gb_ABK94500.1_ len=112 ExpAA=0.14 First60=0.13 PredHel=0 Topology=o

gi_222855013_gb_EEE92560.1_ len=235 ExpAA=0.08 First60=0.00 PredHel=0 Topology=o

gi_113952525_gb_ABI48955.1_ len=361 ExpAA=0.01 First60=0.00 PredHel=0 Topology=o

gi_147771132_emb_CAN60966.1_ len=102 ExpAA=20.45 First60=20.45 PredHel=1 Topology=o10-27i

gi_147828025_emb_CAN72920.1_ len=1303 ExpAA=25.40 First60=6.16 PredHel=0 Topology=o

gi_116781124_gb_ABK21974.1_ len=300 ExpAA=0.01 First60=0.00 PredHel=0 Topology=o

gi_118488894_gb_ABK96256.1_ len=172 ExpAA=21.66 First60=6.57 PredHel=0 Topology=o

gi_225451968_ref_XP_002279750.1_ len=251 ExpAA=0.04 First60=0.00 PredHel=0 Topology=o

gi_125620176_gb_ABN46980.1_ len=97 ExpAA=0.00 First60=0.00 PredHel=0 Topology=o

gi_255550445_ref_XP_002516273.1_ len=358 ExpAA=11.59 First60=11.58 PredHel=0 Topology=o

gi_224284356_gb_ACN39913.1_ len=306 ExpAA=0.47 First60=0.47 PredHel=0 Topology=o

gi_147809570_emb_CAN62388.1_ len=1069 ExpAA=0.03 First60=0.00 PredHel=0 Topology=o

gi_224112371_ref_XP_002316167.1_ len=414 ExpAA=0.07 First60=0.05 PredHel=0 Topology=o

gi_113564263_dbj_BAF14606.1_ len=537 ExpAA=22.85 First60=0.40 PredHel=1 Topology=o461-483i

gi_219547603_gb_ABR04092.2_ len=134 ExpAA=13.62 First60=0.00 PredHel=0 Topology=o

gi_222836942_gb_EEE75335.1_ len=131 ExpAA=0.05 First60=0.00 PredHel=0 Topology=o

gi_10334991_gb_AAG15839.2_AF055910_1 len=310 ExpAA=0.01 First60=0.01 PredHel=0 Topology=o

gi_147844462_emb_CAN82085.1_ len=473 ExpAA=0.04 First60=0.00 PredHel=0 Topology=o

gi_15277979_gb_AAK94021.1_AF400125_2 len=343 ExpAA=5.09 First60=0.00 PredHel=0 Topology=o

gi_291047848_emb_CBK51437.1_ len=526 ExpAA=0.38 First60=0.00 PredHel=0 Topology=o

gi_255543757_ref_XP_002512941.1_ len=488 ExpAA=0.01 First60=0.00 PredHel=0 Topology=o

gi_189036645_sp_A2CI56.1_EFTU_CHLAT len=410 ExpAA=0.09 First60=0.08 PredHel=0 Topology=o

gi_225436924_ref_XP_002275066.1_ len=397 ExpAA=0.24 First60=0.01 PredHel=0 Topology=o

gi_110739579_dbj_BAF01698.1_ len=196 ExpAA=0.00 First60=0.00 PredHel=0 Topology=o

gi_223547873_gb_EEF49365.1_ len=376 ExpAA=0.14 First60=0.00 PredHel=0 Topology=o

gi_125556268_gb_EAZ01874.1_ len=225 ExpAA=0.09 First60=0.05 PredHel=0 Topology=o

gi_222855622_gb_EEE93169.1_ len=291 ExpAA=0.03 First60=0.00 PredHel=0 Topology=o

gi_116788085_gb_ABK24751.1_ len=594 ExpAA=289.39 First60=0.05 PredHel=13 Topology=o132-151i158-180o195-217i229-251o266-286i293-315o339-361i391-410o414-433i446-468o483-502i514-536o565-587i

gi_225459270_ref_XP_002285780.1_ len=484 ExpAA=0.55 First60=0.00 PredHel=0 Topology=o

gi_219911944_emb_CAX03819.1_ len=259 ExpAA=0.03 First60=0.00 PredHel=0 Topology=o

gi_192911936_gb_ACF06576.1_ len=320 ExpAA=0.00 First60=0.00 PredHel=0 Topology=o

gi_118486293_gb_ABK94988.1_ len=320 ExpAA=2.70 First60=0.00 PredHel=0 Topology=o

gi_147799465_emb_CAN70603.1_ len=493 ExpAA=8.12 First60=8.11 PredHel=0 Topology=o

gi_14250864_emb_CAC39223.1_ len=1076 ExpAA=0.58 First60=0.00 PredHel=0 Topology=o

gi_15231677_ref_NP_190843.1_ len=473 ExpAA=4.80 First60=0.00 PredHel=0 Topology=o

gi_113537464_dbj_BAF09847.1_ len=358 ExpAA=0.01 First60=0.00 PredHel=0 Topology=o

gi_187763117_ref_YP_001876592.1_ len=39 ExpAA=22.25 First60=22.25 PredHel=1 Topology=o15-37i

gi_118486617_gb_ABK95146.1_ len=271 ExpAA=0.00 First60=0.00 PredHel=0 Topology=o

gi_257307001_emb_CBC46243.1_ len=531 ExpAA=0.02 First60=0.00 PredHel=0 Topology=o

gi_115456425_ref_NP_001051813.1_ len=186 ExpAA=0.03 First60=0.00 PredHel=0 Topology=o

gi_168060897_ref_XP_001782429.1_ len=194 ExpAA=0.00 First60=0.00 PredHel=0 Topology=o

gi_222844416_gb_EEE81963.1_ len=333 ExpAA=20.30 First60=19.60 PredHel=1 Topology=o10-32i

gi_110742767_dbj_BAE99289.1_ len=380 ExpAA=19.89 First60=19.77 PredHel=1 Topology=i7-29o

gi_113537419_dbj_BAF09802.1_ len=425 ExpAA=0.46 First60=0.00 PredHel=0 Topology=o

gi_222850772_gb_EEE88319.1_ len=352 ExpAA=0.00 First60=0.00 PredHel=0 Topology=o

gi_223531394_gb_EEF33228.1_ len=409 ExpAA=0.00 First60=0.00 PredHel=0 Topology=o

gi_217073546_gb_ACJ85133.1_ len=276 ExpAA=10.06 First60=3.42 PredHel=0 Topology=o

gi_109715590_emb_CAK95628.1_ len=202 ExpAA=0.23 First60=0.02 PredHel=0 Topology=o

gi_117556532_gb_ABK35494.1_ len=144 ExpAA=0.00 First60=0.00 PredHel=0 Topology=o

gi_118483867_gb_ABK93824.1_ len=464 ExpAA=0.19 First60=0.00 PredHel=0 Topology=o

gi_222837256_gb_EEE75635.1_ len=576 ExpAA=0.25 First60=0.00 PredHel=0 Topology=o

gi_18397283_ref_NP_564338.1_ len=1187 ExpAA=0.73 First60=0.00 PredHel=0 Topology=o

gi_42565381_gb_AAS20967.1_ len=159 ExpAA=0.00 First60=0.00 PredHel=0 Topology=o

gi_192910730_gb_ACF06473.1_ len=115 ExpAA=0.01 First60=0.01 PredHel=0 Topology=o

gi_115447069_ref_NP_001047314.1_ len=378 ExpAA=0.08 First60=0.00 PredHel=0 Topology=o

gi_6735325_emb_CAB68151.1_ len=124 ExpAA=0.05 First60=0.05 PredHel=0 Topology=o

gi_169883405_gb_ACA97595.1_ len=650 ExpAA=0.19 First60=0.00 PredHel=0 Topology=o

gi_222423693_dbj_BAH19813.1_ len=551 ExpAA=0.02 First60=0.00 PredHel=0 Topology=o

gi_225452340_ref_XP_002274098.1_ len=146 ExpAA=0.00 First60=0.00 PredHel=0 Topology=i

gi_147772895_emb_CAN73668.1_ len=880 ExpAA=0.05 First60=0.00 PredHel=0 Topology=o

gi_116782403_gb_ABK22493.1_ len=154 ExpAA=0.00 First60=0.00 PredHel=0 Topology=o

gi_116791005_gb_ABK25819.1_ len=273 ExpAA=0.09 First60=0.00 PredHel=0 Topology=o

gi_102139752_gb_ABF69959.1_ len=597 ExpAA=0.00 First60=0.00 PredHel=0 Topology=o

gi_113547517_dbj_BAF10960.1_ len=198 ExpAA=0.10 First60=0.00 PredHel=0 Topology=o

gi_115435186_ref_NP_001042351.1_ len=612 ExpAA=1.85 First60=1.76 PredHel=0 Topology=o

gi_125526519_gb_EAY74633.1_ len=709 ExpAA=4.10 First60=0.00 PredHel=0 Topology=o

gi_115470887_ref_NP_001059042.1_ len=561 ExpAA=0.02 First60=0.00 PredHel=0 Topology=o

gi_147854540_emb_CAN78578.1_ len=612 ExpAA=0.03 First60=0.00 PredHel=0 Topology=o

gi_124301256_gb_ABN04842.1_ len=546 ExpAA=0.37 First60=0.00 PredHel=0 Topology=o

gi_222868651_gb_EEF05782.1_ len=360 ExpAA=0.73 First60=0.73 PredHel=0 Topology=o

gi_224108343_ref_XP_002314814.1_ len=545 ExpAA=0.03 First60=0.02 PredHel=0 Topology=o

gi_218197117_gb_EEC79544.1_ len=1119 ExpAA=293.90 First60=0.00 PredHel=13 Topology=o221-240i349-371o419-441i448-470o480-502i509-531o546-568i575-597o607-624i637-659o701-723i730-752o851-873i

gi_77551219_gb_ABA94016.1_ len=179 ExpAA=0.01 First60=0.00 PredHel=0 Topology=o

gi_145356755_ref_XP_001422591.1_ len=338 ExpAA=0.00 First60=0.00 PredHel=0 Topology=o

gi_134307089_gb_ABO72542.1_ len=365 ExpAA=0.07 First60=0.00 PredHel=0 Topology=o

gi_1155265_gb_AAA85273.1_ len=206 ExpAA=0.08 First60=0.07 PredHel=0 Topology=o

gi_118481003_gb_ABK92455.1_ len=216 ExpAA=0.00 First60=0.00 PredHel=0 Topology=o

gi_222848774_gb_EEE86321.1_ len=1328 ExpAA=0.25 First60=0.00 PredHel=0 Topology=o

gi_219900659_emb_CAW95810.1_ len=553 ExpAA=2.67 First60=0.00 PredHel=0 Topology=o

gi_218185264_gb_EEC67691.1_ len=550 ExpAA=0.06 First60=0.00 PredHel=0 Topology=o

gi_168051195_ref_XP_001778041.1_ len=161 ExpAA=0.00 First60=0.00 PredHel=0 Topology=o

gi_116779023_gb_ABK21104.1_ len=126 ExpAA=0.00 First60=0.00 PredHel=0 Topology=o

gi_116000720_emb_CAL50400.1_ len=349 ExpAA=0.00 First60=0.00 PredHel=0 Topology=o

gi_116785433_gb_ABK23720.1_ len=207 ExpAA=0.02 First60=0.02 PredHel=0 Topology=o

gi_255560331_ref_XP_002521182.1_ len=214 ExpAA=0.02 First60=0.00 PredHel=0 Topology=o

gi_255640048_gb_ACU20315.1_ len=322 ExpAA=0.22 First60=0.00 PredHel=0 Topology=o

gi_223536744_gb_EEF38385.1_ len=174 ExpAA=0.00 First60=0.00 PredHel=0 Topology=o

gi_222862072_gb_EEE99614.1_ len=374 ExpAA=38.43 First60=0.00 PredHel=2 Topology=i166-185o189-206i

gi_112253858_ref_YP_717114.1_ len=190 ExpAA=0.02 First60=0.01 PredHel=0 Topology=o

gi_116789998_gb_ABK25466.1_ len=207 ExpAA=0.00 First60=0.00 PredHel=0 Topology=o

gi_16612249_gb_AAL27496.1_AF439824_1 len=512 ExpAA=0.00 First60=0.00 PredHel=0 Topology=o

gi_118487430_gb_ABK95543.1_ len=89 ExpAA=0.00 First60=0.00 PredHel=0 Topology=o

gi_147798613_emb_CAN72185.1_ len=740 ExpAA=0.01 First60=0.00 PredHel=0 Topology=o

gi_116786824_gb_ABK24254.1_ len=252 ExpAA=115.13 First60=21.09 PredHel=5 Topology=i38-60o70-90i103-125o140-162i183-205o

gi_116781875_gb_ABK22279.1_ len=314 ExpAA=0.03 First60=0.00 PredHel=0 Topology=o

gi_219912060_emb_CAX03877.1_ len=250 ExpAA=0.06 First60=0.00 PredHel=0 Topology=o

gi_11493393_gb_AAG35472.1_U67064_1 len=424 ExpAA=0.05 First60=0.05 PredHel=0 Topology=o

gi_118488068_gb_ABK95854.1_ len=251 ExpAA=0.05 First60=0.00 PredHel=0 Topology=o

gi_225447290_ref_XP_002279657.1_ len=223 ExpAA=0.05 First60=0.05 PredHel=0 Topology=o

gi_195963550_gb_ACG59943.1_ len=416 ExpAA=0.00 First60=0.00 PredHel=0 Topology=o

gi_148905793_gb_ABR16060.1_ len=476 ExpAA=0.57 First60=0.57 PredHel=0 Topology=o

gi_225429846_ref_XP_002280915.1_ len=1084 ExpAA=1.05 First60=0.79 PredHel=0 Topology=o

gi_300153058_gb_EFJ19698.1_ len=349 ExpAA=0.00 First60=0.00 PredHel=0 Topology=o

gi_113205143_gb_AAT40507.2_ len=165 ExpAA=0.00 First60=0.00 PredHel=0 Topology=o

gi_222864119_gb_EEF01250.1_ len=536 ExpAA=0.11 First60=0.00 PredHel=0 Topology=o

gi_148910064_gb_ABR18115.1_ len=430 ExpAA=0.05 First60=0.01 PredHel=0 Topology=o

gi_153850916_gb_ABS52645.1_ len=284 ExpAA=0.03 First60=0.00 PredHel=0 Topology=o

gi_113533241_dbj_BAF05624.1_ len=642 ExpAA=244.48 First60=0.00 PredHel=12 Topology=o122-139i152-174o189-211i224-243o247-266i279-298o323-345i382-401o421-440i445-467o472-494i556-578o

gi_225425356_ref_XP_002275396.1_ len=287 ExpAA=0.00 First60=0.00 PredHel=0 Topology=o

gi_13447112_gb_AAK26638.1_AF342784_1 len=163 ExpAA=0.00 First60=0.00 PredHel=0 Topology=o

gi_125563525_gb_EAZ08905.1_ len=927 ExpAA=1.53 First60=0.00 PredHel=0 Topology=o

gi_123255384_gb_ABM74179.1_ len=81 ExpAA=0.00 First60=0.00 PredHel=0 Topology=o

gi_108707120_gb_ABF94915.1_ len=402 ExpAA=0.00 First60=0.00 PredHel=0 Topology=o

gi_12321295_gb_AAG50716.1_AC079041_9 len=505 ExpAA=1.08 First60=0.05 PredHel=0 Topology=o

gi_108707343_gb_ABF95138.1_ len=1041 ExpAA=0.03 First60=0.03 PredHel=0 Topology=o

gi_147828306_emb_CAN66483.1_ len=625 ExpAA=38.91 First60=16.50 PredHel=2 Topology=i5-24o565-587i

gi_1066153_dbj_BAA07177.1_ len=368 ExpAA=0.36 First60=0.00 PredHel=0 Topology=o

gi_195656867_gb_ACG47901.1_ len=429 ExpAA=0.00 First60=0.00 PredHel=0 Topology=o

gi_194704686_gb_ACF86427.1_ len=446 ExpAA=14.94 First60=14.87 PredHel=0 Topology=o

gi_11994450_dbj_BAB02452.1_ len=500 ExpAA=45.67 First60=18.03 PredHel=2 Topology=o4-21i293-315o

gi_149242538_pdb_2O01_L len=164 ExpAA=56.95 First60=9.90 PredHel=2 Topology=i79-101o135-157i

gi_297842809_ref_XP_002889286.1_ len=405 ExpAA=0.00 First60=0.00 PredHel=0 Topology=o

gi_108708037_gb_ABF95832.1_ len=208 ExpAA=0.00 First60=0.00 PredHel=0 Topology=o

gi_255576966_ref_XP_002529368.1_ len=350 ExpAA=0.01 First60=0.01 PredHel=0 Topology=o

gi_118485650_gb_ABK94675.1_ len=220 ExpAA=16.36 First60=2.69 PredHel=0 Topology=o

gi_147844134_emb_CAN80568.1_ len=421 ExpAA=0.31 First60=0.00 PredHel=0 Topology=o

gi_148906560_gb_ABR16432.1_ len=432 ExpAA=0.51 First60=0.00 PredHel=0 Topology=o

gi_222847662_gb_EEE85209.1_ len=342 ExpAA=8.17 First60=0.00 PredHel=0 Topology=o

gi_113533478_dbj_BAF05861.1_ len=548 ExpAA=0.19 First60=0.04 PredHel=0 Topology=o

gi_163930094_dbj_BAD42856.2_ len=438 ExpAA=9.92 First60=9.85 PredHel=0 Topology=o

gi_118481245_gb_ABK92572.1_ len=197 ExpAA=0.09 First60=0.03 PredHel=0 Topology=o

gi_218190138_gb_EEC72565.1_ len=1218 ExpAA=0.02 First60=0.00 PredHel=0 Topology=o

gi_125541230_gb_EAY87625.1_ len=168 ExpAA=0.03 First60=0.00 PredHel=0 Topology=o

gi_125586123_gb_EAZ26787.1_ len=505 ExpAA=20.58 First60=16.93 PredHel=1 Topology=i40-62o

gi_194692492_gb_ACF80330.1_ len=436 ExpAA=0.01 First60=0.00 PredHel=0 Topology=o

gi_222862287_gb_EEE99793.1_ len=265 ExpAA=0.18 First60=0.14 PredHel=0 Topology=o

gi_147812668_emb_CAN61857.1_ len=521 ExpAA=1.43 First60=1.20 PredHel=0 Topology=o

gi_225458493_ref_XP_002284126.1_ len=389 ExpAA=0.93 First60=0.83 PredHel=0 Topology=o

gi_147818915_emb_CAN69374.1_ len=137 ExpAA=0.00 First60=0.00 PredHel=0 Topology=o

gi_255626457_gb_ACU13573.1_ len=152 ExpAA=0.00 First60=0.00 PredHel=0 Topology=o

gi_14326485_gb_AAK60288.1_AF385695_1 len=268 ExpAA=21.78 First60=0.08 PredHel=1 Topology=i245-267o

gi_147781802_emb_CAN65444.1_ len=298 ExpAA=0.24 First60=0.00 PredHel=0 Topology=o

gi_336930_gb_AAA65856.1_ len=92 ExpAA=0.08 First60=0.06 PredHel=0 Topology=i

gi_270267971_gb_ACZ65565.1_ len=221 ExpAA=25.03 First60=25.02 PredHel=1 Topology=o10-32i

gi_255089519_ref_XP_002506681.1_ len=364 ExpAA=0.12 First60=0.00 PredHel=0 Topology=o

gi_115477583_ref_NP_001062387.1_ len=261 ExpAA=17.17 First60=0.00 PredHel=1 Topology=i92-111o

gi_223532690_gb_EEF34472.1_ len=589 ExpAA=0.00 First60=0.00 PredHel=0 Topology=o

gi_116785563_gb_ABK23774.1_ len=297 ExpAA=14.93 First60=14.91 PredHel=0 Topology=o

gi_147787283_emb_CAN75763.1_ len=340 ExpAA=23.06 First60=0.01 PredHel=1 Topology=i306-328o

gi_297316876_gb_EFH47298.1_ len=230 ExpAA=0.64 First60=0.00 PredHel=0 Topology=o

gi_147835422_emb_CAN69848.1_ len=166 ExpAA=0.00 First60=0.00 PredHel=0 Topology=o

gi_17473511_gb_AAL38379.1_ len=879 ExpAA=0.28 First60=0.22 PredHel=0 Topology=o

gi_147766788_emb_CAN74160.1_ len=1302 ExpAA=9.52 First60=0.00 PredHel=0 Topology=o

gi_147768650_emb_CAN71664.1_ len=294 ExpAA=0.00 First60=0.00 PredHel=0 Topology=o

gi_118486443_gb_ABK95061.1_ len=297 ExpAA=5.58 First60=0.22 PredHel=0 Topology=o

gi_225461597_ref_XP_002282909.1_ len=831 ExpAA=86.91 First60=0.00 PredHel=4 Topology=o538-555i567-589o599-621i774-796o

gi_147838328_emb_CAN67802.1_ len=301 ExpAA=3.60 First60=1.97 PredHel=0 Topology=o

gi_162679333_gb_EDQ65782.1_ len=480 ExpAA=8.94 First60=8.63 PredHel=0 Topology=o

gi_118483244_gb_ABK93525.1_ len=199 ExpAA=0.00 First60=0.00 PredHel=0 Topology=o

gi_225434634_ref_XP_002279688.1_ len=211 ExpAA=0.20 First60=0.04 PredHel=0 Topology=o

gi_108705946_gb_ABF93741.1_ len=279 ExpAA=0.24 First60=0.00 PredHel=0 Topology=o

gi_218195039_gb_EEC77466.1_ len=510 ExpAA=19.45 First60=19.42 PredHel=1 Topology=i7-29o

gi_225217085_gb_ACN85351.1_ len=136 ExpAA=0.00 First60=0.00 PredHel=0 Topology=o

gi_77554888_gb_ABA97684.1_ len=158 ExpAA=0.92 First60=0.00 PredHel=0 Topology=o

gi_116308832_emb_CAH65970.1_ len=911 ExpAA=0.32 First60=0.00 PredHel=0 Topology=o

gi_294464678_gb_ADE77846.1_ len=108 ExpAA=0.59 First60=0.00 PredHel=0 Topology=o

M. cordata 0

DaGuo_2130 len=283 ExpAA=2.13 First60=0.94 PredHel=0 Topology=o

DaGuo_2217 len=1090 ExpAA=5.98 First60=3.29 PredHel=0 Topology=o

DaGuo_2218 len=1077 ExpAA=22.58 First60=15.92 PredHel=0 Topology=o

DaGuo_2343 len=1817 ExpAA=16.20 First60=6.89 PredHel=0 Topology=o

DaGuo_5104 len=206 ExpAA=1.24 First60=0.86 PredHel=0 Topology=o

DaGuo_5827 len=288 ExpAA=3.14 First60=1.37 PredHel=0 Topology=o

DaGuo_7122 len=767 ExpAA=2.63 First60=1.59 PredHel=0 Topology=o

DaGuo_7352 len=216 ExpAA=0.74 First60=0.27 PredHel=0 Topology=o

DaGuo_11735 len=246 ExpAA=6.04 First60=4.00 PredHel=0 Topology=o

DaGuo_17846 len=1005 ExpAA=4.19 First60=3.64 PredHel=0 Topology=o

DaGuo_26184 len=266 ExpAA=1.47 First60=0.20 PredHel=0 Topology=o

DaGuo_28433 len=990 ExpAA=5.75 First60=3.21 PredHel=0 Topology=o

DaGuo_28517 len=529 ExpAA=1.28 First60=0.62 PredHel=0 Topology=o

DaGuo_29586 len=985 ExpAA=3.82 First60=1.97 PredHel=0 Topology=o

DaGuo_29733 len=1080 ExpAA=6.97 First60=4.67 PredHel=0 Topology=o

DaGuo_29734 len=1271 ExpAA=5.31 First60=2.25 PredHel=0 Topology=o

DaGuo_29735 len=1279 ExpAA=21.82 First60=15.91 PredHel=0 Topology=o

DaGuo_29737 len=1275 ExpAA=5.28 First60=3.29 PredHel=0 Topology=o

DaGuo_29789 len=1262 ExpAA=9.66 First60=4.85 PredHel=0 Topology=o

DaGuo_29928 len=1210 ExpAA=5.73 First60=1.38 PredHel=0 Topology=o

DaGuo_30091 len=674 ExpAA=11.32 First60=7.48 PredHel=0 Topology=o

DaGuo_30103 len=378 ExpAA=1.03 First60=0.58 PredHel=0 Topology=o

DaGuo_30314 len=1286 ExpAA=7.92 First60=5.98 PredHel=0 Topology=o

DaGuo_30556 len=1132 ExpAA=6.45 First60=2.82 PredHel=0 Topology=o

DaGuo_30795 len=876 ExpAA=3.16 First60=1.55 PredHel=0 Topology=o

DaGuo_30796 len=1140 ExpAA=6.56 First60=2.31 PredHel=0 Topology=o

DaGuo_30802 len=1169 ExpAA=7.85 First60=5.97 PredHel=0 Topology=o

DaGuo_30803 len=1286 ExpAA=7.91 First60=5.97 PredHel=0 Topology=o

DaGuo_30820 len=842 ExpAA=1.99 First60=0.64 PredHel=0 Topology=o

DaGuo_30866 len=1887 ExpAA=6.74 First60=2.73 PredHel=0 Topology=o

DaGuo_30868 len=387 ExpAA=1.78 First60=1.01 PredHel=0 Topology=o

DaGuo_30869 len=868 ExpAA=28.92 First60=8.50 PredHel=0 Topology=o

DaGuo_30896 len=698 ExpAA=1.90 First60=0.33 PredHel=0 Topology=o

DaGuo_31043 len=1137 ExpAA=2.35 First60=0.91 PredHel=0 Topology=o

DaGuo_31131 len=648 ExpAA=3.88 First60=2.17 PredHel=0 Topology=o

DaGuo_31449 len=585 ExpAA=2.73 First60=1.60 PredHel=0 Topology=o

DaGuo_31450 len=770 ExpAA=3.87 First60=2.75 PredHel=0 Topology=o

DaGuo_31454 len=1746 ExpAA=7.62 First60=1.82 PredHel=0 Topology=o

DaGuo_31455 len=1765 ExpAA=10.22 First60=4.05 PredHel=0 Topology=o

DaGuo_31498 len=1855 ExpAA=17.99 First60=11.80 PredHel=0 Topology=o

DaGuo_32619 len=896 ExpAA=31.04 First60=19.44 PredHel=0 Topology=o

DaGuo_32620 len=696 ExpAA=2.47 First60=0.84 PredHel=0 Topology=o

DaGuo_32625 len=1364 ExpAA=28.17 First60=0.92 PredHel=0 Topology=o

DaGuo_32630 len=1307 ExpAA=25.37 First60=3.72 PredHel=0 Topology=o

DaGuo_32635 len=1223 ExpAA=12.76 First60=11.08 PredHel=0 Topology=o

DaGuo_37522 len=1346 ExpAA=5.30 First60=1.00 PredHel=0 Topology=o

DaGuo_37525 len=1351 ExpAA=6.00 First60=2.51 PredHel=0 Topology=o

DaGuo_37526 len=1083 ExpAA=5.85 First60=3.21 PredHel=0 Topology=o

DaGuo_38366 len=1451 ExpAA=3.55 First60=2.52 PredHel=0 Topology=o

DaGuo_43511 len=1958 ExpAA=2.77 First60=1.42 PredHel=0 Topology=o

DaGuo_43586 len=1745 ExpAA=3.12 First60=1.71 PredHel=0 Topology=o

DaGuo_46373 len=1632 ExpAA=97.11 First60=7.10 PredHel=0 Topology=o

DaGuo_46374 len=1896 ExpAA=96.79 First60=7.10 PredHel=0 Topology=o

DaGuo_51476 len=2241 ExpAA=35.00 First60=5.92 PredHel=0 Topology=o

DaGuo_53633 len=1458 ExpAA=4.77 First60=3.75 PredHel=0 Topology=o

DaGuo_53638 len=708 ExpAA=3.09 First60=1.35 PredHel=0 Topology=o

DaGuo_55210 len=537 ExpAA=3.05 First60=2.26 PredHel=0 Topology=o

DaGuo_59482 len=756 ExpAA=2.03 First60=1.46 PredHel=0 Topology=o

DaGuo_60216 len=599 ExpAA=1.74 First60=0.82 PredHel=0 Topology=o

DaGuo_61449 len=341 ExpAA=3.02 First60=2.09 PredHel=0 Topology=o

DaGuo_62547 len=360 ExpAA=2.02 First60=1.22 PredHel=0 Topology=o

DaGuo_67271 len=302 ExpAA=1.75 First60=0.99 PredHel=0 Topology=o

**M. microcapa**

XiaoGuo_368 len=223 ExpAA=1.45 First60=0.35 PredHel=0 Topology=o

XiaoGuo_542 len=344 ExpAA=2.29 First60=0.95 PredHel=0 Topology=o

XiaoGuo_3055 len=238 ExpAA=2.42 First60=1.61 PredHel=0 Topology=o

XiaoGuo_3439 len=244 ExpAA=22.41 First60=15.85 PredHel=0 Topology=o

XiaoGuo_3620 len=216 ExpAA=1.98 First60=1.48 PredHel=0 Topology=o

XiaoGuo_3720 len=762 ExpAA=1.93 First60=0.78 PredHel=0 Topology=o

XiaoGuo_3722 len=645 ExpAA=4.54 First60=0.81 PredHel=0 Topology=o

XiaoGuo_4952 len=343 ExpAA=18.00 First60=4.91 PredHel=0 Topology=o

XiaoGuo_7428 len=209 ExpAA=4.26 First60=2.91 PredHel=0 Topology=o

XiaoGuo_8047 len=235 ExpAA=3.77 First60=3.23 PredHel=0 Topology=o

XiaoGuo_8073 len=259 ExpAA=4.39 First60=2.04 PredHel=0 Topology=o

XiaoGuo_8485 len=369 ExpAA=5.33 First60=3.32 PredHel=0 Topology=o

XiaoGuo_11344 len=359 ExpAA=0.95 First60=0.36 PredHel=0 Topology=o

XiaoGuo_18101 len=218 ExpAA=1.58 First60=0.94 PredHel=0 Topology=o

XiaoGuo_18425 len=347 ExpAA=2.11 First60=1.42 PredHel=0 Topology=o

XiaoGuo_23150 len=286 ExpAA=2.92 First60=1.49 PredHel=0 Topology=o

XiaoGuo_28845 len=1235 ExpAA=0.88 First60=0.50 PredHel=0 Topology=o

XiaoGuo_32991 len=880 ExpAA=23.09 First60=9.19 PredHel=0 Topology=o

XiaoGuo_35839 len=231 ExpAA=2.47 First60=1.86 PredHel=0 Topology=o

XiaoGuo_37650 len=542 ExpAA=10.37 First60=6.27 PredHel=0 Topology=o

XiaoGuo_37714 len=1228 ExpAA=46.24 First60=5.20 PredHel=0 Topology=o

XiaoGuo_37715 len=1131 ExpAA=19.98 First60=3.36 PredHel=0 Topology=o

XiaoGuo_37945 len=1019 ExpAA=5.81 First60=2.25 PredHel=0 Topology=o

XiaoGuo_37970 len=1264 ExpAA=25.58 First60=4.09 PredHel=0 Topology=o

XiaoGuo_38419 len=1341 ExpAA=8.95 First60=4.75 PredHel=0 Topology=o

XiaoGuo_38420 len=1169 ExpAA=3.97 First60=0.86 PredHel=0 Topology=o

XiaoGuo_38422 len=1262 ExpAA=5.54 First60=2.06 PredHel=0 Topology=o

XiaoGuo_38788 len=1236 ExpAA=11.72 First60=6.31 PredHel=0 Topology=o

XiaoGuo_39144 len=1123 ExpAA=14.31 First60=2.51 PredHel=0 Topology=o

XiaoGuo_39167 len=1121 ExpAA=1.82 First60=0.88 PredHel=0 Topology=o

XiaoGuo_39168 len=1378 ExpAA=25.95 First60=10.06 PredHel=0 Topology=o

XiaoGuo_42571 len=444 ExpAA=2.38 First60=1.86 PredHel=0 Topology=o

XiaoGuo_42572 len=2075 ExpAA=2.74 First60=0.99 PredHel=0 Topology=o

XiaoGuo_42573 len=1859 ExpAA=2.63 First60=1.65 PredHel=0 Topology=o

XiaoGuo_42574 len=1714 ExpAA=19.38 First60=9.51 PredHel=0 Topology=o

XiaoGuo_42575 len=1846 ExpAA=6.65 First60=3.01 PredHel=0 Topology=o

XiaoGuo_42579 len=1183 ExpAA=2.65 First60=1.69 PredHel=0 Topology=o

XiaoGuo_45620 len=335 ExpAA=1.67 First60=0.60 PredHel=0 Topology=o

XiaoGuo_45621 len=2144 ExpAA=13.97 First60=6.95 PredHel=0 Topology=o

XiaoGuo_45622 len=2011 ExpAA=30.58 First60=5.38 PredHel=0 Topology=o

XiaoGuo_45623 len=1769 ExpAA=26.68 First60=4.95 PredHel=0 Topology=o

XiaoGuo_48859 len=1371 ExpAA=1.17 First60=0.50 PredHel=0 Topology=o

XiaoGuo_50254 len=1683 ExpAA=4.29 First60=1.47 PredHel=0 Topology=o

XiaoGuo_50255 len=1427 ExpAA=7.29 First60=1.18 PredHel=0 Topology=o

XiaoGuo_52325 len=1755 ExpAA=4.29 First60=1.63 PredHel=0 Topology=o

XiaoGuo_53280 len=1932 ExpAA=19.42 First60=10.55 PredHel=0 Topology=o

XiaoGuo_53281 len=1738 ExpAA=8.30 First60=1.47 PredHel=0 Topology=o

XiaoGuo_54802 len=1582 ExpAA=4.63 First60=1.91 PredHel=0 Topology=o

XiaoGuo_56047 len=1588 ExpAA=3.38 First60=0.80 PredHel=0 Topology=o

XiaoGuo_56729 len=1218 ExpAA=3.27 First60=1.34 PredHel=0 Topology=o

XiaoGuo_59406 len=679 ExpAA=9.02 First60=2.83 PredHel=0 Topology=o

XiaoGuo_63110 len=548 ExpAA=5.54 First60=2.77 PredHel=0 Topology=o

XiaoGuo_66384 len=265 ExpAA=1.56 First60=0.68 PredHel=0 Topology=o

XiaoGuo_68185 len=642 ExpAA=15.96 First60=13.84 PredHel=0 Topology=o

XiaoGuo_68597 len=718 ExpAA=1.76 First60=0.26 PredHel=0 Topology=o
