# Supplementary material for: Integration of Transcriptome, Proteome and Metabolism Data Reveals the Alkaloids Biosynthesis in Macleaya cordata and Macleaya microcarpa
Source: PLoS One. 2013 Jan 9;8(1):e53409. doi: 10.1371/journal.pone.0053409 (PMC3541140; doi:10.1371/journal.pone.0053409)
Supplement: Methods S1 — Full methods description. (PDF) [file pone.0053409.s016.pdf]

Methods

Preparation of cDNA library for transcriptome sequencing

Total RNA was extracted by modified CTAB method (Zhao et al. 2002) from the 10 samples of both species which were snap-frozen and stored at -80°C until processing. The RNA integrity was confirmed using the Agilent 2100 Bioanalyzer with a minimum integrity number value of 8. Equal amounts of total RNA from each tissue were prepared for cDNA preparation. The poly (A)+ RNA was isolated from 20 μg of the total RNA pool using Dynal oligo(dT) 25 beads (Enzymatics) according to the manufacturer's protocol. Following purification, the mRNA was fragmented into smaller pieces at 70°C for 2 min in the fragmentation buffer (Ambion) and reverse-transcribed to synthesize first strand cDNA using Superscript II reverse transcriptase (Enzymatics) and N6 random hexamers (IDT). Subsequently, second strand cDNA was synthesized using RNase H (Enzymatics) and DNA polymerase (Enzymatics). These cDNA fragments were further processed by an end repair using T4 DNA polymerase, Klenow DNA polymerase, and T4 polynucleotide kinase (Enzymatics), and ligation of adaptors with Illumina's adaptor oligo mix and T4 DNA ligase (Invitrogen). The products were purified for the section of approximate 200 bp long using Qiaquick Gel Extraction Kit (Qiagen) and enriched with PCR for preparing the sequencing library. The cDNA library was detected by Agilent 2100 Bioanalyzer and quantified by ABI StepOnePlus Real-Time PCR System.

Illumina sequencing

The cDNA library was sequenced from both of 5' and 3' ends on the Illumina HiSeq2000 platform according to the manufacturer's instructions. The fluorescent images process to sequences, base-calling and quality value calculation were performed by the Illumina data processing pipeline (version 1.4), in which 90 bp paired-end reads were generated.

De novo assembly of sequencing reads and sequence clustering

Before assembly, the raw reads were filtered to obtain the high-quality clean reads by removing adaptor sequences, duplication sequences, the reads containing more than 10% "N" rate (the "N" character representing ambiguous bases in reads), and low-quality reads containing more than 50% bases with Q-value ≤ 5. The Q-value is the quality score assigned to each base by the Illumina's base-caller Bustard from the Illumina pipeline software suite (version 1.4), similar to the Phred score of the base call. De novo assembly of the clean reads was performed using Trinity program, which implements a de Bruijn graph algorithm and a stepwise strategy (Grabherr et al. 2011). Trinity combines three independent software modules: Inchworm, Chrysalis, and Butterfly, applied sequentially to process large volumes of RNA-Seq reads. Briefly, Inchworm assembles the RNA-Seq reads into the unique sequences of transcripts, often generating full-length transcripts for a dominant isoform, but then reports just the unique portions of alternatively spliced transcripts according to the greedy strategy. Next, Chrysalis clusters the contigs generated from Inchworm into clusters and constructs complete de Bruijn graphs for each cluster. Each cluster represents the full transcriptional complexity for a given gene (or sets of genes that share sequences in common). Chrysalis then partitions the full read set among these disjoint graphs. Finally, Butterfly processes the individual graphs in parallel and reports full-length transcripts. To reduce the sequence redundancy, the transcripts of M. cordata and M. microcarpa were clustered using TGICL (assembler for multiple alignments and consensus building) individually. Transcripts that cannot reach the threshold set and fall into any assembly should remain as a list of singletons. Raw sequence reads data have been deposited in the NCBI Sequence Read Archive with the following study identifiers: M.cordata transcriptome-[SRA: SRA048772] and M.microcarpa transcriptome-[SRA: SRA048780].

Functional annotation and classification

All assembled unigenes (consensuses and singletons) longer than 200 bp were annotated by the assignments of putative gene descriptions, conserved domains, Gene

Ontology terms, and putative metabolic pathways to them based on sequence similarity with previously identified genes annotated with those details. For assignments of predicted gene descriptions, the assembled unigenes were compared to Uniprot protein database respectively using BLASTALL procedure (ftp://ftp.ncbi.nih.gov/blast/executables/ release/2.2.18/) with a significant threshold of E-value$\leqslant 10^{-5}$. To parse the features of the best BLASTX hits from the alignments, putative gene names, 'CDS', and predicted proteins of corresponding assembled sequences can be produced.

Since a large portion of assembled unigenes have not yet been annotated, conserved domains/families were further identified in the assembled unigenes using the Pfam database 2011(version 24.0) (Punta et al. 2011) and Clusters of Orthologous Groups database at NCBI (Tatusov et al. 2001). Domain-based comparisons with the Pfam and COGs databases were performed using HMMER3 (http:// hmmer.janelia.org) and BLAST programs (E-value: 10^{-5}), respectively. Functional categorization by Gene Ontology terms (GO; http://www.geneontology.org) (Ashburner et al. 2000) was carried out based on two sets of best BLASTX hits from UniProt database with E-value threshold of 10^{-5}, then the annotation results were displayed using WEGO web service. The KEGG pathways annotation was performed by sequence comparisons against the KEGG database (Kanehisa et al. 2004) using BLASTX algorithm (E-value: 10^{-5}).

Analysis of differential gene expression

The differential gene expression was analyzed using DESeq, which is an R package. It provides a method to test for differential expression by use of the negative binonial distribution and a shrinkage estimator for the distribution's variance. DEseq expects count data of each unigene in the form of a matrix of integer values. To obtain such a count table, we calculate each unigene's corresponding raw reads with RSEM software(Li and Dewey 2011). DEseq was feed with RESM's results, and generate statistical information such as expression level, fold change, p value and FDR. We filter the results with following conditions: 1) fold change greater than 2; 2)

Expression level ("baseMean" in terms of DESeq) of one of the two samples greater than 50; 3) p values lower than 0.01 and FDR lower than 0.5.

We analyzed differentially expressed genes of the same tissue at different phases, and the different tissues at the same phase. We also compared the gene expression of the same tissues between two species. To analyze DEGs of different species, a double best hit approach was used, which means the best hit of A in M.cordata is B in M. microcarpa and the best hit of B is A in M.microcarpa, thus A and B is a double best hit pair. The expression was compared between these double best hit pairs.

Identification of isoquinoline alkaloid biosynthetic enzymes

The sequences of isoquinoline alkaloid biosynthetic enzymes from other plants are downloading from UniProt database. The assembled unigenes of M. cordata and M. microcarpa were compared to these sequences respectively using BLASTX with a significant threshold of E-value < 10^{-10} and identity > 50%. The most similar unigenes were considered as each enzyme's corresponding unigenes. The sum of these unigenes' RPKM values give an inspect to the expression level of these enzymes, then hierarchical clustering of log-transformed expression data was carried out using R.

Cytochrome P450 and ABC transporter analysis

We downloaded the cytochrome P450 sequences of Arabidopsis and Rice from the P450 database (http://drnelson.uthsc.edu/CytochromeP450.html). We also searched Genbank to obtain the opium poppy and *Coptis japonica* specific P450 sequences as the "addition" P450 group, which may be Papaveraceae family specific P450 proteins. Then, we used the M. cordata and M. microcarpa unigenes to BLAST the P450 database (BLASTX E-value < 10^{-10} and similarity>50%), the most similar unigenes were collected as the corresponding unigenes of each cytochrome P450. Then the unigenes of M. cordata and M. microcarpa were assigned to P450 families and hierarchical clustering of log-transformed expression data was carried out.

The analysis of ABC transporter is in similar protocol. The ABC transporter sequences of Arabidopsis was downloaded TAIR (www.arabidopsis.org) and then M.

cordata and M. microcarpa unigene database were blasted to them (BLASTX E-value $< 10^{-10}$ and similarity>50%). We also calculated each ABC transport's expression level, and analyzed the distribution of ABC transporter families.

Real-time RT-PCR

Four selected unigenes of key enzymes with potential roles in Isoquinoline alkaloid biosynthesis pathway were chosen for validation using real time qPCR with gene specific primers designed with Primer3 software (see primer list in supplemental Table S4). Total RNA was extracted from roots, leaves and fruit shells of the M. cordata plant using a modified TRIzol method (Rio et al. 2010) and purified with RNA purification kit (Tiangen, China). Reverse transcription was performed using the High Capacity cDNA Reverse Transcription Kit (Applied Biosystems) with either oligo d(T) or random primers. The standard curve for each gene was obtained by real-time PCR with several dilutions of cDNA. Real-time PCR was performed using SYBR Green PCR Master Mix (Applied Biosystems) on the ABI 7300 Real-time PCR system according to the manufacturer's instructions. Quantifying the relative to ubiquitin (UBI; GenBank accession: GQ901904)expression of the genes in three different organs was calculated by normalizing Ct values using the $2^{-\Delta\Delta Ct}$ method (Livak and Schmittgen 2001).

Protein Extraction for iTRAQ Analysis

Plant tissues from 8 different tissues of the *Macleaya* plant [both *Macleaya cordata* (Willd.) R. Br. and *Macleaya microcarpa* (Maxim)Fedde], including Phase I(leaves) and Phase II(roots, leaves and shells) which were snap-frozen and stored at -80°C until processing. These samples are from exact the same plant and the same phrase for RNA extraction, and because the use of 8-Plex iTRAQ we have to eliminate one pair of samples which we choose the roots in Phase I. The proteomics research of *Macleaya* roots in different stage will report in another research work that we have done recently with the 2D-DIGE. Samples were then washed twice with phosphate buffered saline (PBS) before being lysed in 1M triethylammonium bicarbonate (B),

pH 8.5 with 0.05% (w/v) SDS. Lysed tissues were scraped off the surface of the flask into 1.5 ml eppendorf tubes kept on ice, and then mechanically homogenized using a mini hand-held homogenizer. The supernatant was collected following centrifugation at 10,000g for 30 min at 4℃. The amount of protein in each sample was determined using the Bio-Rad RC DC protein assay (Hertfordshire, UK), and the samples stored in aliquots at -80℃ until use.

Isobaric Tag Labeling

Sample labeling with iTRAQ reagents was performed as described previously(Ye et al. 2010). Briefly, 100 mg of protein from each cell line was reduced, alkylated then digested with trypsin, prior to labeling (Applied Biosystems, Framingham, MA). Labeling was as follows: DGY1501 (113), DGG1501 (114), DGU1501 (115), XGY1502 (116), XGG1502 (117), XGU1502 (118), DGY9801 (119) and XGY9802 (121). The labeled samples were combined, vacuum-evaporated, and stored at -20℃ prior to fractionation by strong-cation exchange liquid chromatography (SCX HPLC).

Strong Cation Exchange Fractionation of Peptides

The fractionation was performed as described previously (Glen et al. 2008). Briefly, SCX was carried out using a PolySULFOETHYLTM A Column (PolyLC, Columbia, MD) 5 mm particle size of 200mm length_2.1mm id, 200Å pore size, on a BioLC HPLC unit (Dionex, Surrey, UK). The 60-min gradient was generated between BufferA(10mM KH_2PO_4 and 25% acetonitrile,pH3.0), and Buffer B (10mM KH_2PO_4, 25% acetonitrile and 500mM KCl, pH 3.0), and consisted of 100% A for 5 min, 5–30% B for 40 min, 30–100% B for 5 min, 100% B for 5 min and finally 100% A for 5min. The chromatogram was monitored through a UV Detector (Dionex/ LC Packings, Amsterdam, the Netherlands), at a wavelength of 280, 254, and 214 nm. Fractions were collected every minute and were later pooled together according to variations in peak intensity. Twenty SCX fractions were pooled for subsequent nano-LC-MS/MS analysis. Pooled fractions were dried in a vacuum concentrator, and stored at-20℃ prior to mass spectrometric analysis.

Mass Spectrometric Analysis

All LC-MS MS experiments were performed on an LTQ Orbitrap Velos (Thermo Fischer Scientific) equipped with a Famos autosampler (LC Packings) and an Agilent 1100 binary high-pressure liquid chromatography (HPLC) pump (Agilent Technologies). The peptide mixture was separated on a PepMap C-18 RP capillary column (LC Packings), with a constant flow rate of 0.3 ml/min. The LC gradient started with 3% Buffer B (0.1% formic acid in 97% acetonitrile) and 97% Buffer A (0.1% formic acid in 3% acetonitrile) for 3 min, followed by 3–30% Buffer B for 90 min, then 90%Buffer B for 7min, and finally 3% Buffer B for 8 min. The mass spectrometer was set to perform data acquisition in the positive ion mode, with a selected mass range of 300– 2,000m/z. Peptides with t2 to t4 charge states were selected for tandem mass spectrometry, and the time of summation of MS/MS events was set to 3 sec. The two most abundantly charged peptides above a five count threshold were selected for MS/MS, and dynamically excluded for 60 sec with a≥50mmu mass tolerance.

Protein Identification and Relative Quantification

Protein identification and quantification for iTRAQ was carried out using the Scaffold Q+ (version Scaffold_3.1.2, Proteome Software Inc., Portland, OR) as reported previously (Glen et al. 2008). The search was performed against three databases (one from the NCBInr whole plant database，and two are of the species to transcribe the coding area protein database that the origin(gene) gets behind with net database BLAST). A concatenated target-decoy database search strategy was also employed to estimate the rate of false positives (Elias and Gygi 2007).

Bias normalization for whole proteome analyses were also performed by correcting the bias median ratio of each comparison toward unity. Only proteins identified with at least 95% confidence or ProtScore of ≥1.3 were reported. A measure for false-positive (FP) discovery rate was also calculated using the total identification spectra selected from a concatenated database composed the ‘‘forward’’ and

‘‘reverse’’ of our synthesize database. Systematic sequence reversal was performed with a Perl script, while the search was performed using Scaffold with identical parameters. Protein quantitative ratios were calculated as the median of all peptide ratios. Standard deviations were calculated as the interquartile range around the median. Quantitative ratios were Log2 ratio normalized for final quantitative testing.

HPLC-UV analysis

A Waters 5125 HPLC system (Waters Corporation, USA) coupled with UV detector was used for quantitative determination of four alkaloids. The UV detector was employed at the wavelength of 284 nm. Peak area was used for quantification. Chromatographic separation was carried out on a XB-C18 analytical column (4.6×250 mm，5 μm，Welch Material, USA) at 35 ℃. A linear gradient elution of A (100% acetonitrile) and B (0.1% phosphoric acid aqueous solution) was used. The time program for the multi-step gradient was shown as follows: initial 25% (A), 0−14 min keeping 25% (A), 14−27 min linear gradient to 60% (A), 27−29 min linear gradient to 25% (A), keeping 25% (A) at 29−34 min. The flow rate was 0.8 mL/min, and the injection volume was 5 μL.

Scanning electron microscopic (SEM) and optical microscope analysis

For SEM sample preparation: leaves and roots samples (about 1 mm^2) were fixed with 2.5% glutaraldehyde solution overnight at 4°C, then washed with 0.1 M phosphate buffer (pH 7.0) three times. The samples were refixed in 1% (v/v) OsO4 solution for 2 h and dehydrated in a graded acetone series. The samples were embedded in Spurr’s resin then stained with saturated uranyl acetate in 50% ethanol and 0.2% (w/v) aqueous lead citrate for 15 min each. An ultrathin coating of electrically-conducting material was deposited by high vacuum evaporation of platinum to improve the contrast. The sections were examined and photographed with a JSM-6380LV microscope (JEOL, Akishima, and Tokyo, Japan).

For optical microscope sample preparation: cut sample materials into 0.5-1 cm small

pieces and then put into 6% sodium hydroxide with 1: 30 physical volume ratios , 40 ℃ for 36 hours. Then wash three times with the distilled water and add 1-2 drops of bismuth potassium iodide. Add 2-3 drops of 70% sulfuric acid after the 1-2 mins, and then check under microscope after 3 minutes.

Reference

Ashburner, M., C.A. Ball, J.A. Blake, D. Botstein, H. Butler, J.M. Cherry, A.P. Davis, K. Dolinski, S.S. Dwight, J.T. Eppig, M.A. Harris, D.P. Hill, L. Issel-Tarver, A. Kasarskis, S. Lewis, J.C. Matese, J.E. Richardson, M. Ringwald, G.M. Rubin, and G. Sherlock. 2000. Gene ontology: tool for the unification of biology. The Gene Ontology Consortium. *Nat Genet* **25:** 25-29.

Elias, J.E. and S.P. Gygi. 2007. Target-decoy search strategy for increased confidence in large-scale protein identifications by mass spectrometry. *Nat Methods* **4:** 207-214.

Glen, A., C.S. Gan, F.C. Hamdy, C.L. Eaton, S.S. Cross, J.W. Catto, P.C. Wright, and I. Rehman. 2008. iTRAQ-facilitated proteomic analysis of human prostate cancer cells identifies proteins associated with progression. *J Proteome Res* **7:** 897-907.

Grabherr, M.G., B.J. Haas, M. Yassour, J.Z. Levin, D.A. Thompson, I. Amit, X. Adiconis, L. Fan, R. Raychowdhury, Q. Zeng, Z. Chen, E. Mauceli, N. Hacohen, A. Gnirke, N. Rhind, F. di Palma, B.W. Birren, C. Nusbaum, K. Lindblad-Toh, N. Friedman, and A. Regev. 2011. Full-length transcriptome assembly from RNA-Seq data without a reference genome. *Nat Biotechnol* **29:** 644-652.

Kanehisa, M., S. Goto, S. Kawashima, Y. Okuno, and M. Hattori. 2004. The KEGG resource for deciphering the genome. *Nucleic Acids Res* **32:** D277-280.

Li, B. and C.N. Dewey. 2011. RSEM: accurate transcript quantification from RNA-Seq data with or without a reference genome. *BMC Bioinformatics* **12:** 323.

Livak, K.J. and T.D. Schmittgen. 2001. Analysis of relative gene expression data using real-time quantitative PCR and the 2(-Delta Delta C(T)) Method. *Methods* **25:** 402-408.

Punta, M., P.C. Coggill, R.Y. Eberhardt, J. Mistry, J. Tate, C. Boursnell, N. Pang, K. Forslund, G. Ceric, J. Clements, A. Heger, L. Holm, E.L. Sonnhammer, S.R. Eddy, A. Bateman, and R.D. Finn. 2011. The Pfam protein families database. *Nucleic Acids Res*.

Rio, D.C., M. Ares, Jr., G.J. Hannon, and T.W. Nilsen. 2010. Purification of RNA using TRIzol (TRI reagent). *Cold Spring Harb Protoc* **2010:** pdb prot5439.

Tatusov, R.L., D.A. Natale, I.V. Garkavtsev, T.A. Tatusova, U.T. Shankavaram, B.S. Rao, B. Kiryutin, M.Y. Galperin, N.D. Fedorova, and E.V. Koonin. 2001. The COG database: new developments in phylogenetic classification of proteins from complete genomes. *Nucleic Acids Res* **29:** 22-28.

Ye, H., L. Sun, X. Huang, P. Zhang, and X. Zhao. 2010. A proteomic approach for plasma biomarker discovery with 8-plex iTRAQ labeling and SCX-LC-MS/MS. *Mol Cell Biochem* **343:** 91-99.

Zhao, S.Y., Y.R. Wu, and G.M. Xia. 2002. [Introduction of a simple and effective method for plant total RNA isolation]. *Yi Chuan* **24:** 337-338.
